# Aquatic Invertebrate Antimicrobial Peptides in the Fight Against Aquaculture Pathogens

**DOI:** 10.3390/microorganisms13010156

**Published:** 2025-01-14

**Authors:** Tomás Rodrigues, Francisco Antonio Guardiola, Daniela Almeida, Agostinho Antunes

**Affiliations:** 1 CIIMAR—Interdisciplinary Centre of Marine and Environmental Research, University of Porto, Terminal de Cruzeiros do Porto de Leixões, Av. General Norton de Matos, s/n, 4450-208 Porto, Portugal; tomasfcr.porto@gmail.com; 2 Department of Biology, Faculty of Sciences, University of Porto, Rua do Campo Alegre 687, 4169-007 Porto, Portugal; 3 Immunobiology for Aquaculture Group, Department of Cell Biology and Histology, Faculty of Biology, Regional Campus of International Excellence “Campus Mare Nostrum”, University of Murcia, 30100 Murcia, Spain; faguardiola@um.es; 4 Department of Zoology and Physical Anthropology, Faculty of Biology, Regional Campus of International Excellence “Campus Mare Nostrum”, University of Murcia, 30100 Murcia, Spain; daniela.martins@um.es

**Keywords:** antimicrobial peptides, aquatic invertebrates, aquaculture, bioactive compounds, cryptides, pathogens, antimicrobial resistance, antibacterial, antifungal, antiviral

## Abstract

The intensification of aquaculture has escalated disease outbreaks and overuse of antibiotics, driving the global antimicrobial resistance (AMR) crisis. Antimicrobial peptides (AMPs) provide a promising alternative due to their rapid, broad-spectrum activity, low AMR risk, and additional bioactivities, including immunomodulatory, anticancer, and antifouling properties. AMPs derived from aquatic invertebrates, particularly marine-derived, are well-suited for aquaculture, offering enhanced stability in high-salinity environments. This study compiles and analyzes data from AMP databases and over 200 scientific sources, identifying approximately 350 AMPs derived from aquatic invertebrates, mostly cationic and α-helical, across 65 protein families. While in vitro assays highlight their potential, limited in vivo studies hinder practical application. These AMPs could serve as feed additives, therapeutic agents, or in genetic engineering approaches like CRISPR/Cas9-mediated transgenesis to enhance resilience of farmed species. Despite challenges such as stability, ecological impacts, and regulatory hurdles, advancements in peptidomimetics and genetic engineering hold significant promise. Future research should emphasize refining AMP enhancement techniques, expanding their diversity and bioactivity profiles, and prioritizing comprehensive in vivo evaluations. Harnessing the potential of AMPs represents a significant step forward on the path to aquaculture sustainability, reducing antibiotic dependency, and combating AMR, ultimately safeguarding public health and ecosystem resilience.

## 1. Introduction

The transition to intensive aquaculture has increased population densities, resulting in stress and weakened immune systems in farmed species, which increases their susceptibility to infectious diseases. These diseases contribute to high mortality rates, reduced productivity, and significant economic losses [1]. To address these issues, antibiotics were introduced, initially reducing disease-related mortality and supporting industrial growth [2]. However, antibiotic overuse promoted the resurgence of bacterial diseases, with the emergence of new pathogens and the reoccurrence of older infections, driven by the rapid spread of AMR [3,4]. Bacteria develop resistance through various mechanisms, such as the use of efflux pumps to actively expel antibiotics, the production of enzymes that degrade or modify antibiotics, and horizontal gene transfer, which allows the exchange of resistance genes between bacterial populations, including from nonpathogenic to pathogenic strains [5,6,7]. Furthermore, bacterial mutations change antibiotic targets, resulting in the development of treatment-resistant strains that cause more severe and prolonged infections, leading to increased healthcare costs [8]. Nevertheless, there is currently no comprehensive global system to regulate and monitor the use of antimicrobial agents in aquaculture. Despite the approval of only a limited number of antibiotics for use, they are still widely and indiscriminately applied, especially in major aquaculture-producing countries in Asia [9,10]. The unrestricted use of antibiotics in aquaculture poses environmental risks, including accumulation in culture systems, disruption of microbial communities, and harm to non-target organisms. It promotes the development of antibiotic-resistant microbial strains, reducing antibiotic efficacy, threatening aquaculture sustainability, and posing human health risks through the consumption of seafood contaminated with resistant bacteria [11]. Therefore, it is crucial to explore alternative strategies to address pathogens and AMR in aquaculture, such as the use of AMPs.

AMPs are evolutionarily conserved, gene-encoded natural molecules with diverse functional and structural properties. They have a wide range of biological activities, including antibacterial, antifungal, antiviral, antiparasitic, anticancer, antibiofilm, immunomodulatory, wound healing, and anti-inflammatory [12,13,14,15,16,17,18,19]. These molecules provide distinct advantages, including rapid and direct antimicrobial action, broad-spectrum activity against bacteria, viruses, fungi, and parasites, and a reduced risk of contributing to AMR, making them highly attractive for clinical applications [20,21]. In metazoans, AMPs evolved through recurrent gene duplications, leading to the emergence of paralogs, and balancing/positive selection in response to bacterial pathogens [22,23,24]. The first AMP, gramicidin, was isolated in 1939 from a soil *Bacillus* strain [25]. Since then, numerous AMPs have been isolated, many of which are specific to certain taxa or species [26]. Their common features include being composed of 10–100 amino acids, having a molecular weight below 25–30 kDa, and a positive net charge [27,28,29]. Despite their common features, AMPs vary widely in structural motifs and secondary structures—such as α-helical, β-sheet, αβ motifs, and random coils—with similar sequences often adopting different conformations, leading to diverse classifications [30,31]. AMPs can be natural (NAMPs), directly isolated from natural sources, or produced synthetically (SAMPs) [32]. While NAMPs may have issues like instability, low solubility, toxicity, and salt sensitivity [33], SAMPs are designed based on NAMPs with modifications to enhance bioactivity. These modifications include the addition of specific amino acid residues, sequence truncation to retain only active regions, cyclization, and peptidomimetics for improved stability [26,34,35,36].

AMPs exhibit diverse modes of action to target and disrupt microbial cells. Most AMPs primarily target cell membranes through several proposed mechanisms, including the toroidal pore, barrel-stave, carpet, and agglutination models. In the toroidal pore model, peptides integrate into the membrane, forming pores that remain associated with the membrane’s polar groups [37]. In the barrel-stave model, peptides create transmembrane pores that form a central cavity and detach after formation [38]. The carpet model involves peptides interacting with the membrane surface, disrupting its structure and causing the formation of multiple pores [39]. In the agglutination model, peptides aggregate bacterial cells into a micellar complex, preventing membrane penetration [40]. Alternatively, some AMPs interfere with intracellular processes, such as protein synthesis, nucleic acid functions, and enzyme activity, impairing cell wall formation. Other AMPs disrupt cellular energy metabolism by inhibiting ATP synthase or interfering with the electron transport chain, ultimately leading to cell death [41]. New AMPs are discovered through several approaches, including direct extraction from cells or tissues followed by identification via mass spectrometry, cDNA cloning of AMP genes using primers targeting conserved regions, and omics-based methods that integrate genomic, proteomic, or transcriptomic data with bioinformatics analysis [42]. Additionally, the development of advanced AMP prediction tools like amPEPpy [43] and AMPlify [44], which use artificial intelligence and machine learning tailored to predicting and characterizing AMPs, alongside AlphaFold2 [45], which utilizes deep learning to predict protein structures from amino acid sequences, has greatly improved the discovery and characterization of new AMPs. While amPEPpy and AMPlify focus specifically on AMP prediction, AlphaFold2 provides a more generalized solution for predicting the structures of proteins, offering faster and more accurate predictions than traditional methods like X-ray crystallography, Nuclear Magnetic Resonance (NMR), and cryogenic electron microscopy (cryo-EM), which are accurate but time-consuming and labor-intensive. While advances in DNA sequencing have vastly increased the number of known protein sequences, the structures of many remain undetermined. AlphaFold has revolutionized the field by providing a faster and more accurate way to predict protein structures, helping bridge the gap between the substantial number of known sequences and the limited number of experimentally determined structures. Several AMP databases, including APD3 [46], CAMPR4 [47], DBAASP [48], dbAMP 2.0 [49], DRAMP 3.0 [50], and Inverpep [51], serve as useful repositories for cataloging known AMPs. However, these resources face limitations such as data redundancy, lack of standardization, and inadequate curation. The StarPep graph database, which integrates over 45,000 peptide sequences and metadata from 42 databases, including more than 22,600 AMPs, represents a notable effort to address these issues [52]. Nonetheless, a significant data gap for AMPs derived from aquatic invertebrates still persists, given the sparsity of relevant information, which is dispersed across numerous studies, and often lacks structural information.

Our study provides a comprehensive review of AMPs derived from aquatic invertebrates. We focus on the AMPs’ activities against pathogens relevant to both aquaculture and human health, including antibacterial, antifungal, antiviral, antiparasitic, antibiofilm, and anticancer properties. Furthermore, we address gaps in knowledge about the structures of these AMPs and explore their potential applications for the aquaculture industry.

## 2. Data Collection and Analysis

A comprehensive data review was conducted to compile AMPs derived from aquatic invertebrates, classified within the Marine, Brackish, and Freshwater categories as defined by the World Register of Marine Species (WoRMS) database [53].

Data on the AMPs were sourced from over 200 scientific articles and several databases, including the National Center for Biotechnology Information (NCBI) GenBank [54], UniProt [55], RCSB Protein Data Bank (RCSB PDB) [56], and StarPep [52]. The selection of articles was based on specific inclusion criteria, prioritizing peer-reviewed studies with clear methodologies, and peptides with documented experimental evidence of antimicrobial activity, either through antimicrobial assays or gene expression analyses following antimicrobial challenges. Articles lacking sufficient data or relevance to the review’s focus were excluded. Duplicate records and inconsistencies were systematically checked and removed.

Given the focus on AMP activity against pathogens relevant to aquaculture and human health, the pathogenic status of the reported microbial agents was verified by consulting over 180 articles and books, e.g., [57,58,59] (Table S1). The study adhered to the accepted nomenclature from the List of Prokaryotic names with Standing in Nomenclature (LPSN) [60] for bacteria, MycoBank [61] for fungi and fungi-like organisms, and the NCBI Taxonomy browser [62] for viruses and parasites.

The physical and chemical properties of AMPs, including net charge, molecular weight (MW), isoelectric point (pI), and the grand average of hydropathy (GRAVY), were calculated using a custom Biopython script based on the ProtParam tool from the Expasy Proteomic Server [63]. Signal peptides were predicted using SignalP 6.0 [64], and propeptides were predicted using ProP 1.0 with minimum probability confidence of 70% [65]. Mature AMP sequences were analyzed against the Pfam database [66] using InterProScan [67] to identify protein domains and functional annotations. Structural predictions were made using ColabFold [68] with the AlphaFold2 model [45], generating for each peptide, five independent models with 10 recycles to enhance prediction accuracy. Model relaxation ensured physical plausibility and energetic stability, and the highest-ranked model was selected for structure visualization using ChimeraX [69]. ColabFold and AlphaFold2 provide significant benefits in terms of speed, accuracy, and cost-effectiveness for predicting AMP structures. However, they may face challenges with highly flexible or disordered peptides, which are common in AMPs and may not fully capture the dynamic nature of AMP-pathogen interactions essential to their antimicrobial activity. Despite these limitations, these tools accelerate the understanding of AMP structures in the absence of experimental data, thereby supporting further experimental validation.

## 3. Aquaculture Pathogens

Pathogen control is a major challenge in aquaculture, where densely populated, intensive farming systems facilitate the rapid spread of infections, emphasizing the need for effective disease management strategies [70]. A comprehensive approach includes proper husbandry practices, the development of disease-resistant strains, and the implementation of robust biosecurity protocols, such as quarantine procedures, water treatment methods, and regular health monitoring. Additionally, preventive strategies, including vaccination, the use of immunostimulants, and the application of probiotics, as well as advances in pathogen biology and epidemiology, are essential for improving treatment strategies and reducing disease outbreaks [71,72,73].

Pathogens, including bacteria, fungi, viruses, and parasites, cause significant diseases in farmed aquatic organisms in both marine and freshwater systems. These diseases result in high mortality rates, reduced productivity, and substantial economic losses [74]. Among these, bacterial pathogens are the most prevalent, causing diseases such as columnaris disease, edwardsiellosis, furunculosis, septicemia, streptococcosis, ulcerative diseases, and vibriosis. Genera such as *Aeromonas*, *Edwardsiella*, *Enterobacter*, *Flavobacterium*, *Pseudomonas*, *Staphylococcus*, *Streptococcus*, and *Vibrio* have developed multidrug-resistant strains, which complicate treatment efforts [75,76]. These bacteria use mechanisms like biofilm formation, iron acquisition, extracellular polysaccharide production, and lytic enzyme production to enhance their virulence and persistence in aquaculture systems [77].

Fungal and fungi-like pathogens also pose significant threats. Water molds, like *Saprolegnia* and *Aphanomyces*, were initially classified as fungi due to their morphology and feeding behavior but are now recognized as oomycetes [78]. Differentiating between true fungi (e.g., members of *Ascomycota*, *Basidiomycota*, and *Mucoromycota*) and oomycetes is crucial for developing effective treatments [79]. While true fungi typically infect external fish tissues, oomycetes can invade internal organs and are commonly found in aquaculture systems [73,80].

Viral diseases represent an increasing concern in aquaculture, with significant pathogens including Infectious Salmon Anaemia Virus (ISAV), viral hemorrhagic septicemia virus (VHSV), infectious hematopoietic necrosis virus (IHNV), Shrimp hemocyte iridescent virus (SHIV), Abalone Herpesvirus (AbHV), and White Spot Syndrome Virus (WSSV) [81]. Several factors contribute to the growing concern, including the high mortality rates associated with viral infections and the lack of effective therapeutic options compared to bacterial infections, which can often be treated with antibiotics. In contrast, viral diseases primarily rely on preventive measures, such as vaccination, which is not available for all pathogens [82]. Moreover, viral infections can spread rapidly in densely populated aquaculture systems and may remain asymptomatic in the early stages, complicating early detection and containment. The rapid mutation rates and ability of viruses to evolve quickly further challenge control efforts, as new strains may emerge that evade existing measures [82]. Additionally, parasitic infestations caused by sea lice and protozoans further reduce production yields and increase treatment costs [83,84].

**Figure 1:**
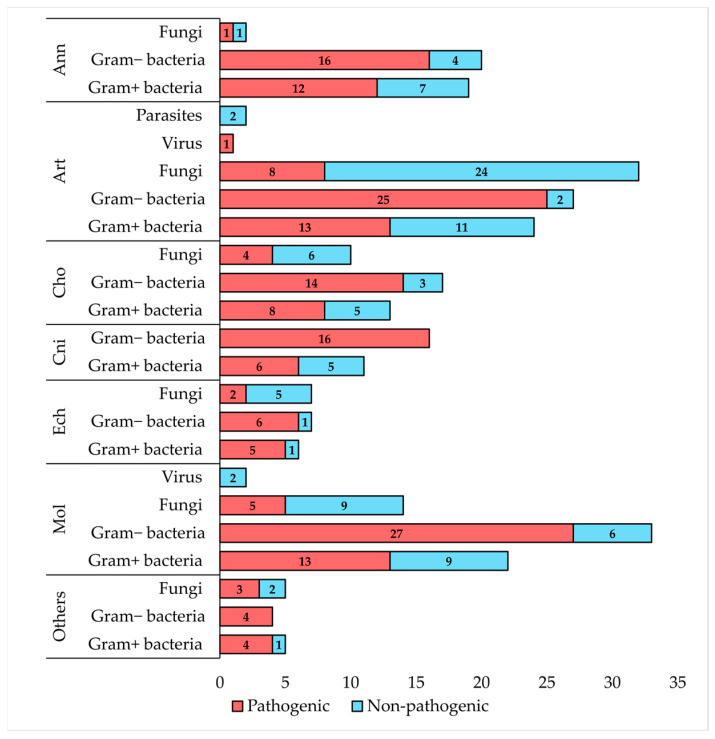
Distribution of antimicrobial activities of aquatic invertebrate AMPs against various pathogens. The chart categorizes microorganisms into Gram-positive bacteria, Gram-negative bacteria, fungi, viruses, and parasites, showing their pathogenic (red) and non-pathogenic (blue) statuses. Each taxonomic group (*Ann*: *Annelida*, *Art*: *Arthropoda*, *Cho*: *Chordata*, *Cni*: *Cnidaria*, *Ech*: *Echinodermata*, *Mol*: *Mollusca*, *Others*: *Nematoda*, *Placozoa*, *Platyhelminthes*, *Porifera*) highlights the diversity and potential antimicrobial targets of AMPs, emphasizing their relevance to aquaculture pathogens.

In this study, the antimicrobial activities of aquatic invertebrate-derived AMPs were compiled, with a focus on pathogens relevant to aquaculture (Figure 1). A detailed list of microorganisms, along with their pathogenic significance for aquaculture and human health, is provided in Table S1.

Notably, aquatic invertebrate-derived AMPs demonstrated activity against a wide range of pathogens, including 47 Gram-negative bacteria, 25 Gram-positive bacteria, 11 fungi and fungi-like organisms, and the virus WSSV. Among these, the Gram-negative bacterium *E. coli*, the Gram-positive bacterium *S. aureus*, and the fungus *C. albicans* were identified as the primary targets of these AMPs.

## 4. AMPs and Other Antimicrobial Agents Isolated from Aquatic Invertebrates

**Figure 2:**
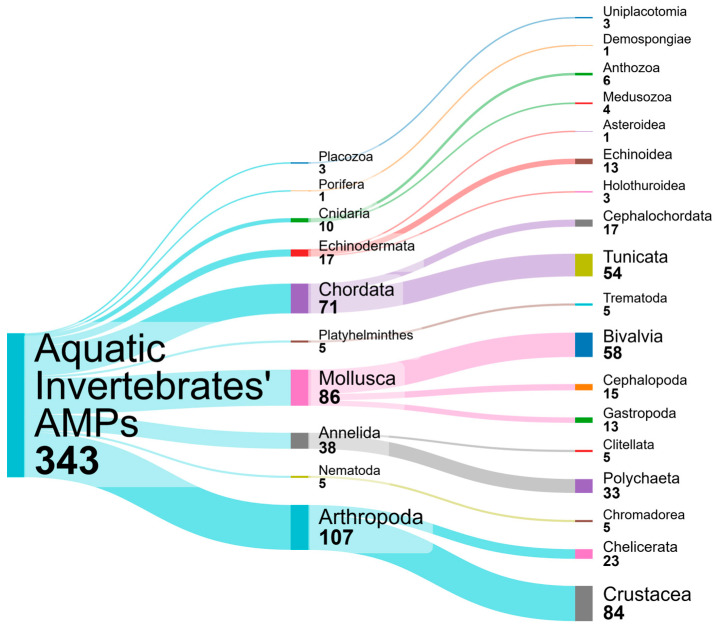
Phylogenetic distribution of currently known AMPs from aquatic invertebrates by phylum and subphylum/class.

Aquatic invertebrates are globally distributed and remarkably diverse, representing ancient animal lineages with diversified adaptations to overcome microbial threats in environments increasingly impacted by anthropogenic factors [85]. Traditionally, invertebrates, unlike vertebrates, were thought to lack an adaptive immune system, relying solely on an innate immune system that includes both cellular and humoral responses mediated by pattern recognition receptors [86]. However, recent evidence suggests that some invertebrates exhibit immunological priming leading to enhanced long-term immune responses, although the underlying mechanisms remain largely unexplored [87]. The cellular immune response in invertebrates involves defense mechanisms such as encapsulation, nodule formation, and phagocytosis, mediated by motile cells known as hemocytes found in the hemolymph. The humoral immune response, on the other hand, is characterized by the presence of antimicrobial substances in the blood cells and plasma, together with responses such as hemolymph coagulation [88]. AMPs are an integral part of the humoral defense system of invertebrates, protecting against infection primarily by disrupting microbial plasma membranes and compromising microbial integrity [83]. AMPs from aquatic invertebrates typically possess cationic and hydrophobic properties, which are critical for effectively targeting key components of microbial cell walls and membranes [89]. Specifically, marine-derived AMPs have evolved to thrive in the unique conditions of the seawater, characterized by its high salinity. This adaptation has led to enhanced electrostatic interactions, increased stability, and a broader antimicrobial spectrum, ultimately making them more potent antimicrobial agents [90]. Invertebrates are the major source of AMPs among aquatic organisms, with approximately 40 families of AMPs characterized to date [20]. This study focuses on invertebrates and the content is organized into sub-chapters based on phyla, providing a comprehensive overview of the diverse range of AMPs derived from aquatic invertebrates (Figure 2, Table S2).

### 4.1. Non-Phylum-Specific AMPs

#### 4.1.1. Defensins

Defensins are small, cysteine-rich cationic AMPs involved in innate immunity across a wide range of organisms, including invertebrates. These proteins are classified into α-, β-, and θ-defensins, with invertebrates producing β-defensins and big defensins. β-defensins feature a conserved β-sheet structure stabilized by three disulfide bridges and show antimicrobial activity against bacteria, fungi, and viruses [91]. Big defensins, considered ancestral to vertebrate β-defensins, feature a bipartite structure with an N-terminal hydrophobic domain and a C-terminal β-defensin-like domain, making them particularly effective against Gram-negative bacteria by disrupting their outer membranes. This disruption occurs as the hydrophobic N-terminal domain inserts into the membrane, forming pores that cause leakage of the cellular content, leading to bacterial death [92]. Defensins have been identified in various invertebrate phyla, including Arthropoda [93,94,95,96,97], Chordata [98], Cnidaria [99,100,101], Mollusca [102,103,104,105,106,107,108,109,110,111,112,113,114,115,116,117], and Porifera [118].

#### 4.1.2. Macins

Macins are a family of cationic, cysteine-rich AMPs with a disulfide-stabilized αβ structural motif. The first macin, theromacin, was isolated from the shallow-water leech *Theromyzon tessulatum* [119]. Since then, macins have been identified in various aquatic invertebrates, including other annelids [120,121,122], cnidarians [123], and mollusks [124,125,126,127,128,129,130,131,132]. Despite their high sequence identity, macins exhibit distinct biological activities, such as membrane-aggregating and permeabilizing effects against Gram-positive bacteria and Gram-negative bacteria [119,120,121,122,130,132,133,134]. Some macins also promote central nervous system regeneration [120,134] and display antibiofilm properties [130,132].

### 4.2. Annelida

The phylum Annelida, comprising approximately 20,200 species, is categorized into three groups: Clitellata, Polychaeta, and Sipuncula [135]. These aquatic and semi-aquatic organisms include clam worms, sand worms, tube worms, and leeches [136]. Extremophile worms are adapted to inhabit harsh environments like abyssal depths, hydrothermal vents, polar regions, and polluted areas, making them valuable for studying novel bioactive compounds [137]. AMPs derived from these organisms often exhibit exceptional properties, such as acid resistance, thermostability, salt tolerance, and broad-spectrum antibacterial activity [138]. In this study, 38 AMPs distributed into six AMP families were identified from 14 aquatic annelids (three Clitellata, 11 Polychaeta). These AMPs have antimicrobial activity against 41 microbial species, including 29 pathogens relevant to aquaculture: 12 Gram-positive bacteria, 16 Gram-negative bacteria, and the fungus *Candida albicans* (Table S3).

#### 4.2.1. Clitellata AMPs

**Figure 3:**
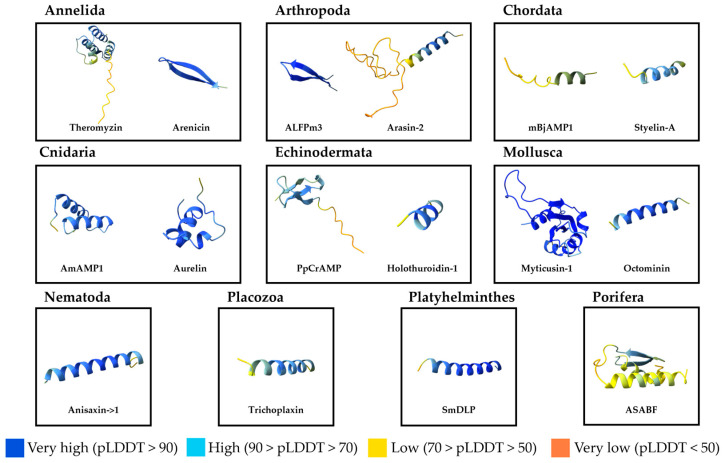
Examples of AMPs from aquatic invertebrate phyla.

Lumbricins are proline-rich AMPs first identified in the earthworm *Lumbricus rubellus* [139] and later found in other earthworms [140,141,142]. Hm-lumbricin was discovered in *H. medicinalis* where its expression is upregulated upon exposure to *D. nishinomiyaensis*, indicating a role in innate immunity. Additionally, Hm-lumbricin has been shown to promote central nervous system regeneration, linking immune defense with tissue repair [120]. Theromyzin is a linear anionic α-helical peptide extracted from the coelomic fluid of *T. tessulatum* (Figure 3). It is the first anionic AMP identified in invertebrates [119].

#### 4.2.2. Polychaeta AMPs

Polychaete AMPs are often found in species from fluctuating thermal habitats, where factors like temperature, salinity, and pressure may influence their evolution and function.

BRICHOS-related AMPs are characterized by a conserved structure comprising a hydrophobic region (signal peptide or transmembrane domain), a proregion containing the BRICHOS domain, and a carboxy-terminal (C-terminal) double-stranded β-sheet [143]. The BRICHOS domain acts as an intramolecular chaperone, facilitating protein targeting to the secretory pathway and protease-mediated processing [144]. Highly conserved across species, this domain is associated with diseases such as Alzheimer’s, Parkinson’s, cancer, diabetes, dementia, and respiratory distress [145,146]. In annelids, BRICHOS-related peptides demonstrate diverse precursor peptides, reflecting adaptation to fluctuating thermal habitats [147,148]. The first BRICHOS-related AMP, arenicin, was found in the extremophile lugworm *A. marina*. This β-hairpin peptide, stabilized by a disulfide bond, inspired synthetic derivatives with enhanced biological properties [149,150,151,152,153,154,155,156]. Subsequent discoveries include abarenicin from AbA. pacifica and UuBRI-21 from *Urechis unicinctus* [157]. Other BRICHOS-related AMPs include α-helical nicomicin from the Arctic polychaeta *Nicomache minor*, which displays anticancer properties [42], and alvinellacin, the first AMP from a deep-sea organism, found in the thermotolerant Pompeii worm *Alvinella pompejana*. Alvinellacin and its homolog capitellacin, from *Capitella teleta*, form a double-stranded β-sheet stabilized by two disulfide bonds [158,159,160]. Recent discoveries of polaricin from the Antarctic worm *Amphitritides* sp., the α-helical HfBRI-28 and β-hairpin HfBRI-25 from *Heteromastus filiformis*, and AmBRI-44a, a defensin-like peptide stabilized by four disulfide bonds from *A. marina*, expand the structural repertoire of BRICHOS-related AMPs [148,161,162].

Hedistin is a cationic α-helical peptide extracted from the coelomic fluid of the sandworm *Hediste diversicolor*. It contains two brominated tryptophan residues and a C-terminal amide [163]. Bromination, a rare post-translational modification, occurs at the 6-position of the tryptophan indole ring, typically found in marine organisms due to the high bromide content and (halo)peroxidases in seawater [164]. Perinerin, a cationic α-helical peptide, was isolated from the Asian clam worm *P. aibuhitensis*. Its full structure remains partially unknown, but it likely contains two intramolecular disulfide bridges [165].

### 4.3. Arthropoda

Arthropods represent the most abundant and diverse group of animals, comprising four subphyla: Chelicerata, Crustacea, Hexapoda, and Myriapoda. Despite the continuous discovery of new species, only a small fraction is aquatic. It is estimated that there are between 100,000 and 110,000 arthropod species, with nearly 100,000 species of aquatic insects from 12 orders, representing 60% of all aquatic animal species [166,167]. However, the assessment of aquatic arthropod diversity is biased due to several factors. There are more taxonomic studies on terrestrial arthropods than on aquatic ones, geographic data is limited, identification efforts primarily target insects and large crustaceans, and there is a global decline in taxonomists and scientific expeditions [168]. Arthropods represent one of the most prominent taxa for the discovery of AMPs with several already identified. In this study, 107 AMPs distributed into 19 AMP families were identified from 22 aquatic arthropods (four Chelicerata, 18 Crustacea). These AMPs were tested and showed antimicrobial activity against 86 microbial species, including 47 pathogens relevant to aquaculture: 13 Gram-positive bacteria, 25 Gram-negative bacteria, eight fungi, and the WSSV (Table S4).

#### 4.3.1. Anti-Lipopolysaccharide Factor

Anti-lipopolysaccharide factors (ALFs) are essential immune peptides in arthropods (both Chelicerata and Crustacea) that bind and neutralize bacterial lipopolysaccharides (LPS), the primary component of Gram-negative bacterial outer membranes, thereby regulating immune responses [169]. They primarily target the lipid A component of LPS, disrupting membrane integrity and neutralizing LPS-induced toxicity through electrostatic and hydrophobic interactions, with some ALFs sequestering LPS to prevent host receptor binding [90,170]. Their main structural feature is the lipopolysaccharide-binding domain (LBD), a conserved region with a disulfide loop formed by two cysteines, crucial for antimicrobial activity [170]. First identified in horseshoe crabs *Limulus polyphemus* and *Tachypleus tridentatus* [171], ALFs have since been found in crabs [172,173,174,175,176] as well as shrimp and prawns [177,178,179,180].

#### 4.3.2. Chelicerata AMPs

Horseshoe crabs, the closest living relatives of trilobites, have thrived for up to 500 million years, originating in Mesozoic European waters before migrating as seas receded. Today, *Carcinoscorpius rotundicauda*, *T. tridentatus*, and *Tachypleus gigas* inhabit Asia’s coasts, while *Limulus polyphemus* is found along North America’s Atlantic coast. These ancient arthropods produce unique AMPs with diverse bioactivities [181,182,183]. Tachyplesin, first discovered in *T. tridentatus* and later in *C. rotundicauda* and *T. gigas*, is a short cationic peptide with two disulfide bonds that exhibits antibacterial, antifungal, anticancer, and antibiofilm activities. Cyclized forms show similar bioactivities with enhanced stability and therapeutic potential [184,185,186,187,188]. Polyphemusin, isolated from *L. polyphemus*, is structurally similar to Tachyplesin, and includes variants with optimized amphipathic properties and comparable bioactivities [185,187,189]. Tachycitin, discovered from *T. tridentatus*, features a stabilized αβ-motif structure with a chitin-binding domain and a C-terminal region with 10 cysteine residues. Its antimicrobial activity is synergistically enhanced with big defensin [190,191]. Tachystatin is a β-sheet chitin-binding peptide from *T. tridentatus* stabilized by three disulfide bonds. Isoforms A and B are homologous, while isoform C has amphiphilic and hemolytic properties [192,193,194]. A thermally stable, non-cytotoxic variant, tachystatin-A2, was identified in *T. gigas* [192,195]. Tatritin is a chitin-binding peptide from *T. tridentatus* with an N-terminal α-helix and a C-terminal β-sheet stabilized by six disulfide bonds, which showed interesting antimicrobial effects [196,197].

#### 4.3.3. Crustacea AMPs

Arasins are proline-rich, cationic AMPs with two distinct domains: a proline/arginine-rich N-terminal region and a cysteine-rich C-terminal region with four cysteines [198]. The first arasin identified, callinectin, was found in the blue crab *Callinectes sapidus* [199,200]. Arasins were then found in the hemocytes of various crustaceans, including crabs [198,201,202], crayfish [203], and prawns [204]. Notably, the proline/arginine-rich N-terminal region of Ha-arasin1 exhibits in vitro chitin-binding activity, indicating its potential role in targeting pathogens with chitin-containing structures [205]. Crustins are cationic AMPs primarily found in crustaceans, first identified in the hemocytes of the shore crab *Carcinus maenas* as a cysteine-rich 11.5-kDa peptide [206]. The term “crustins” was introduced when similar peptides were discovered in penaeid shrimp [207]. They typically feature one or two whey acidic protein (WAP) domains at the C-terminal, each with eight cysteines linked by four disulfide bonds, and a signal sequence at the N-terminal with a variable region between the signal sequence and WAP domain [208]. Crustins are classified into several types based on structural variations. Type I crustins have a cysteine-rich region between the signal peptide and WAP domain, further divided into Ia, Ib, and Ic, with Type Ib having a longer C-terminal region and Type Ic featuring two linked cysteine-rich domains [209]. Type II crustins have both glycine-rich and cysteine-rich regions, with subgroups IIa and IIb differing in the glycine-rich domain length [210]. Type III crustins have a short proline/arginine-rich N-terminal region [208]. Additional types include Type IV, which has two WAP domains [211,212], Type V, which is exclusive to insects and has a cysteine-rich domain and an aromatic amino acid-rich region preceding the WAP domain [213], Type VI, consisting of a glycine-rich region and a WAP domain, and Type VII, with a serine/leucine-rich region followed by a cysteine-rich region and a WAP domain [209]. Crustins are known for their antibacterial and antifungal activities, which vary by type. Type I crustins are particularly effective against Gram-positive bacteria, while Types II and III target both Gram-positive and Gram-negative bacteria [214,215]. Type IV crustins are antiproteolytic, with some exhibiting antibacterial and antifungal properties [216]. Certain crustins have demonstrated antiviral activity [217], antibiofilm properties [218], and wound-healing potential [219], highlighting their multifunctional roles in immune defense and tissue repair. Many AMPs from crabs are rich in specific amino acids that contribute to their antimicrobial properties. Proline-rich AMPs, for instance, exhibit membrane permeability and non-lytic mechanisms that inhibit protein synthesis and induce bacterial death [220,221]. Notable examples include the 6.5-kDa proline-rich peptide from *C. maenas*, the first AMP discovered in crustaceans [222], and SpPR-AMP1 from *S. paramamosain* [223]. Glycine-rich AMPs include GRPSp featuring glycine-rich motifs flanked by two C-terminal cysteine residues, and the thermally stable Spgly-AMP26-62, both from *S. paramamosain* [224,225]. Hyastatins are multi-domain chitin-binding cationic peptides with a conserved six-cysteine C-terminal region. The first hyastatin, discovered in the spider crab *Hyas araneus*, contains a glycine-rich N-terminal region followed by a proline/arginine-rich short region. Their antibacterial and chitin-binding functions are mainly attributed to the cysteine-rich domain, but both the cysteine-rich and proline/arginine-rich regions contribute to their antimicrobial activity [226,227]. Recent transcriptomic studies in *P. trituberculatus* have identified 14 distinct hyastatin variants, indicating significant peptide diversity [228]. Paralithocins, identified in the red king crab *Paralithodes camtschaticus*, are cysteine-rich cationic peptides with eight cysteine residues forming four disulfide bridges [229]. Other AMPs from crabs include scygonadin, an anionic peptide from the seminal plasma of *S. serrata*. It is expressed in the ejaculatory duct and plays a role in maintaining a sterile environment essential for successful fertilization [230,231,232,233]. Sparanegtin, an anionic peptide from *S. paramamosain* containing three α-helices, binds to pathogen-associated molecular patterns (PAMPs) like LPS and peptidoglycan. It plays a crucial role in immune defense during spermatogenesis and modulates immune gene expression in response to bacterial infections. By reducing bacterial loads in critical tissues like the gills and hepatopancreas, sparanegtin enhances the survival of infected crabs [234]. Astacidins are small, cationic, proline/arginine-rich AMPs found exclusively in crayfish, where they bind to PAMPs and play a crucial role in host defense. Initially identified in the invasive *Pacifastacus leniusculus* and later in *P. clarkii*, astacidins are classified into two groups: astacidin 1, derived from the C-terminal region of hemocyanin, and astacidin 2, which consists of proline/arginine-rich peptides with an amidated C-terminus [235,236,237]. Recent research also identified four proline-rich, non-cytotoxic, astacidin 2-related peptides in the transcriptome of *P. clarkii* [238]. Prawns and shrimp are aquatic crustaceans that differ in taxonomy and morphology. Prawns belong to Dendrobranchiata, and have larger, straighter bodies, longer legs, and branching gills, inhabiting both freshwater and saltwater environments. Shrimp, under Pleocyemata, are smaller, with curled bodies, shorter legs, and lamellar gills, primarily found in marine habitats. Misclassifications exist, such as “penaeid shrimp” from the Penaeidae family, which are actually prawns. Penaeidins, cationic AMPs unique to *Penaeus*, have a proline-rich N-terminal and cysteine-rich C-terminal [239]. First identified in *P. vannamei*, they are classified into four subgroups: PEN2, PEN3, and PEN4, which primarily target Gram-positive bacteria and fungi, and PEN5, which also targets Gram-negative bacteria and viruses [240,241,242,243,244,245]. Some penaeidins exhibit chitin-binding properties [246], while structural variants like MjPen- II in *P. japonicus* with a serine-rich N-terminal and BigPEN in *P. vannamei* with an extra repeat region at its N-terminus further highlight their diversity [245,247,248]. Stylicins are anionic AMPs exclusive to *Penaeus*, featuring a proline-rich N-terminal and a cysteine-rich C-terminal with 13 conserved cysteines [249,250,251,252,253]. Stylicins target filamentous fungi, exhibit strong LPS, prevent biofilm formation, and aid immunity against WSSV [250,251,252,253,254].

### 4.4. Chordata

The phylum Chordata includes a diverse array of animals primarily defined by the presence of a notochord and a dorsal hollow nerve chord. It is divided into three subphyla: Cephalochordata (amphioxus), Tunicata (ascidians, larvaceans, and thaliaceans), and Vertebrata (amphibians, birds, fishes, reptiles, and mammals) [165]. The limited number of known AMPs derived from invertebrate chordates is restricted to one amphioxus species and five ascidians. In this study, 71 AMPs distributed into 11 AMP families were identified from seven invertebrate chordates (one Cephalochordata, six Tunicata). These AMPs were tested and showed antimicrobial activity against 40 microbial species, including 26 pathogens relevant to aquaculture: eight Gram-positive bacteria, 14 Gram-negative bacteria, and four fungi (Table S5).

#### 4.4.1. Cephalochordata AMPs

A single AMP, BjAMP1, has been identified in the amphioxus *Branchiostoma japonicum*, exhibiting a unique mode of action. Its structure comprises two α-helices connected by a reverse turn, forming an amphipathic arrangement of polar and hydrophobic residues. This configuration enables BjAMP1 to penetrate bacterial cell membranes without causing structural disruption. Instead, it employs a membranolytic mechanism that induces membrane depolarization and permeabilization. BjAMP1 also binds to LPS and LTA within bacterial membranes and may further inhibit biological functions by interacting with DNA or RNA after membrane penetration, ultimately inducing cell death. It shows broad-spectrum antibacterial activity while remaining non-toxic to mammalian cells [255,256]. Synthetic analogs of mBjAMP1, developed through amino acid modifications, have shown enhanced antimicrobial and antibiofilm properties [257].

#### 4.4.2. Tunicata AMPs

The majority of invertebrate AMPs derived from the phylum Chordata have been identified in ascidians [258]. In *Styela clava*, a variety of AMPs have been identified, including phenylalanine-rich styelins, α-helical clavanins, and histidine-rich clavaspirin. These AMPs have been shown to exhibit antibacterial activity against both Gram-positive and Gram-negative bacteria, as well as fungi [259,260,261,262]. A unique group of amphipathic α -helical AMPs isolated from the hemocytes of *Halocynthia aurantium* have been described which includes halocidin and dicynthaurin. Both peptides have an unusual structural motif consisting of two amphipathic helices that are covalently linked through a single disulfide bond formed between adjacent single cysteine residues. Dicynthaurin is composed of two 30 amino acid monomers bounded together by a single cysteine disulfide bond. Both the peptide monomers and the dimer have an α-helical conformation. Dicynthaurin has a broad-spectrum antibacterial activity [263,264]. Halocidin is composed of an 18 amino acid residues monomer and a 15 residues monomer covalently linked by a single cysteine disulfide bond. Antimicrobial assays verified that the 18-residue monomer is more active than the 15-residue monomer. Moreover, several synthetic Halocidin analogs have been developed with enhanced antimicrobial activities including di-K19Hc [265]. In *Halocynthia papillosa*, the AMPs halocyntin and papillosin demonstrated broad-spectrum antibacterial activity [266]. The identification of AMPs in *Ciona intestinalis* commenced with a search of its expressed sequence tag (EST) database, which yielded the identification of Ci-MAM-A24 and Ci-PAP-A22 [267,268]. Another study predicted over 180 potential AMPs from the small open reading frames (sORF) of *C. instestinalis*. The researchers synthesized the ten most promising candidates, of which five (P-02, P-03, P-04, P-05, and P-10) demonstrated antibacterial activity [269]. Recently, two novel cysteine-rich AMPs, designated as turgencins, along with their oxidized derivatives, were isolated from the Arctic ascidian *Synoicum turgens*. The peptides are post-translationally modified, containing six cysteines with unusual disulfide connectivity of C1-C6, C2-C5, and C3-C4, as well as an amidated C-terminus. Furthermore, the peptides contain methionine residues, resulting in the isolation of peptides with different degrees of oxidation. The most potent peptide, turgencin AMox1, which contains one oxidized methionine, displayed antimicrobial activity against both Gram-negative and Gram-positive bacteria. Additionally, the peptide inhibited the growth of the melanoma cancer cell line A2058 and the human fibroblast cell line MRC-5 [270]. Moreover, 11 synthetic peptides based on turgencin were designed and designated StAMP-1 to StAMP-11. The most potent overall was StAMP-9, which demonstrated potent antimicrobial activity against Gram-positive and Gram-negative bacteria and fungi, as well as non-hemolytic activity against sheep red blood cells. It also exhibited non-cytotoxic effects against the human melanoma cell line A2058 and the non-malignant human lung fibroblast cell line MRC-5, indicating high selectivity [271].

### 4.5. Cnidaria

Cnidaria is a diverse and ancient phylum of predominantly marine organisms, comprising approximately 12,000 extant species [272,273]. These organisms are characterized by the presence of cnidae—organelle-like capsules containing venomous eversible tubules used for defense and prey capture [274,275]. Cnidarians are categorized into three main clades: Anthozoa (anemones, corals, sea fans, sea pens, zoanthids), Medusozoa (hydroids, jellyfish, siphonophores), and Myxozoa (obligate endoparasites) [276,277]. Their defense mechanisms primarily rely on innate immunity, although there is evidence suggesting the presence of immune memory in some species [278]. The cnidarian immune system encompasses immune recognition, intracellular signaling cascades, effector responses, and tissue repair mechanisms [279]. In this study, 10 AMPs distributed into 10 AMP families were identified from eight cnidarians (six Anthozoa, two Medusozoa). These AMPs were tested and showed antimicrobial activity against 27 microbial species, including 22 pathogens relevant to aquaculture: six Gram-positive bacteria and 16 Gram-negative bacteria (Table S6).

#### 4.5.1. Anthozoa AMPs

A few AMPs have been extracted from coral species, including damicornin from the stony coral *Pocillopora damicornis*. This peptide, containing six cysteine residues, exhibits activity against Gram-positive bacteria and the fungus *Fusarium oxysporum*. Notably, the coral pathogen *Vibrio coralliilyticus* represses the expression of the damicornin gene, providing the first evidence of AMP gene repression mediated by *Vibrio* [280]. Another six-cysteine peptide, AmAMP1, was identified in the stony coral *Acropora millepora*. It is expressed in ectodermal cells during late coral development and shows antibacterial activity against a broad range of Gram-positive and Gram-negative bacteria [281]. Additionally, Pd-AMP1, a peptide with a β-hairpin structure isolated from the soft coral *Phyllogorgia dilatata*, showed activity against Gram-positive bacteria [282]. Many AMPs have been identified in sea anemones, such as crassicorin isolated from *Urticina crassicornis*. This peptide adopts a double β-hairpin structure, contains six cysteines, and exhibits antibacterial activity [283]. Recently, equinins were discovered in *Actinia equina*, demonstrating low antibacterial activity and no hemolytic effects on human cells [284].

#### 4.5.2. Medusozoa AMPs

The first AMP identified in a cnidarian, aurelin, was isolated from the mesoglea of the moon jellyfish *Aurelia aurita*. This six-cysteine peptide, composed of two helices linked by a random coil, shows antibacterial activity [285,286]. In *Hydra*, the arminin family of α-helical peptides was discovered, with arminin 1a-C (the C-terminal region of arminin 1a) showing strong antibacterial activity and selective anticancer effects, suppressing leukemia cell viability without causing hemolysis in human erythrocytes [287,288]. Another *Hydra*-derived AMP, periculin-1, features an anionic N-terminal region and a cationic C-terminal region with eight cysteine residues. Expressed in the germline of female *Hydra*, periculin-1 plays a crucial role in selectively targeting bacterial colonization during embryogenesis, demonstrating potent antimicrobial activity [122,289].

### 4.6. Echinodermata

The phylum Echinodermata (from the Greek echinos, “spiny”, and derma, “skin”) includes over 7500 species [53]. These marine organisms, with few species found in brackish waters [290,291], are divided into five classes: Asteroidea (sea stars), Crinoidea (feather stars and sea lilies), Echinoidea (sea urchins, sand dollars, and sea biscuits), Holothuroidea (sea cucumbers), and Ophiuroidea (brittle stars) [135]. Echinoderms possess an innate immune system mediated by coelomic fluid, containing coelomocytes that defend against pathogens and injuries, along with antimicrobial components such as lysozymes and AMPs [292]. Although genetic sequencing has revealed a complex repertoire of immune genes, the echinoderm immune system remains poorly understood, though recent studies have led to the discovery of new AMPs [293]. In this study, 17 AMPs distributed into five AMP families were identified from six echinoderms (one Asteroidea, four Echinoidea, and one Holothuroidea). These AMPs were tested and showed antimicrobial activity against 20 microbial species, including 13 pathogens relevant to aquaculture: five Gram-positive bacteria, six Gram-negative bacteria, and two fungi (Table S7).

#### 4.6.1. Asteroidea AMPs

The only known AMP derived from a sea star, *Patiria pectinifera*, was named *P. pectinifera* cysteine-rich antimicrobial peptide (PpCrAMP). It is predicted to adopt a β-hairpin structure with two extended β-strands linked by a random coil region and exhibit antibacterial activity [294].

#### 4.6.2. Echinoidea AMPs

Sea urchins produce interesting AMPs with notable properties. Strongylocins, first identified in *Strongylocentrotus droebachiensis*, are cationic peptides featuring a distinct cysteine pattern of six residues and a brominated tryptophan. These peptides exhibit strong antibacterial activity and have been also identified in *Strongylocentrotus purpuratus* and *Echinus esculentus* [295,296,297]. Centrocins, also from *S. droebachiensis*, are heterodimeric peptides composed of a 30-residue antimicrobial heavy chain linked by a disulfide bond to a 12-residue inactive light chain. Similar to strongylocins, native centrocins contain a post-translational brominated tryptophan. These peptides display strong antibacterial activities, some antifungal activity, and have also been found in *E. esculentus* [297,298].

#### 4.6.3. Holothuroidea AMPs

Holothuroidins isolated from *Holothuria tubulosa* are the only characterized AMPs derived from a sea cucumber. While these small cationic α-helical peptides exhibit weak antibacterial potency, they also display interesting antibiofilm activity [299,300].

### 4.7. Mollusca

Mollusca, the second largest phylum of invertebrates, includes over 50,000 species [53]. Of its eight classes, Bivalvia (clams, cockles, mussels, oysters, and scallops), Cephalopoda (octopuses, squids, cuttlefishes, and nautilus), and Gastropoda (snails and slugs) account for more than 95% of its diversity [165]. Mollusks are extensively studied for AMPs, with several AMPs reported, mostly from bivalves [258]. In this study, 86 AMPs distributed into 17 AMP families were identified from 28 mollusks (17 Bivalvia, three Cephalopoda, and eight Gastropoda). These AMPs were tested and showed antimicrobial activity against 71 microbial species, including 45 pathogens relevant to aquaculture: 13 Gram-positive bacteria, 27 Gram-negative bacteria, and five fungi (Table S8).

#### 4.7.1. Molluscidin

Molluscidins are AMPs with repeated dibasic residues, identified in the gills of mollusks such as the bivalves *Crassostrea gigas* [301] and *Atrina pectinata* [302], and the gastropod *Haliotis discus* [303]. Their predicted non-amphipathic structures alternate between α-helical and random coil conformations. Molluscidins exhibit antimicrobial activity against Gram-positive and Gram-negative bacteria while maintaining low cytotoxicity.

#### 4.7.2. Bivalvia AMPs

Bivalves, particularly mussels, produce diverse cysteine-rich AMPs essential for their immune defense. These include mytilins [101,104,304], mytimycins [101,305,306], myticins [307,308,309,310,311], and myticusins [130,312,313], all derived from precursor peptides comprising a signal peptide, a mature cysteine-rich region, and a C-terminal extension. Mytilins, mytimycins, and myticins show activity against bacteria, fungi, and viruses, while myticusins also target parasites. Mussels also produce other AMPs, such as myticalins, cationic peptides with α-helical and random coil structures active against Gram-positive and Gram-negative bacteria [314,315,316], and mytichitins, which contain chitin-binding domains that enhance antimicrobial activity against bacteria, fungi, and parasites [317,318]. In non-mussel bivalves, AMPs include Ap from the shellfish *Argopecten purpuratus*, a cationic proline-rich peptide with strong antifungal properties [319], and Cg-Prp from *C. gigas*, a proline-rich peptide with limited direct antimicrobial effects but significant synergy with defensin Cg-Def. This synergistic activity may compensate for the low concentrations of antimicrobials in the oyster tissues [320,321]. Additionally, in *C. hongkongensis*, the cationic α-helical peptide URP20 exhibits potent antimicrobial activity against bacteria and fungi without cytotoxicity to mammalian cells [322].

#### 4.7.3. Cephalopoda AMPs

Despite extensive research on cephalopod extracts, the isolation and characterization of peptides with antimicrobial properties remain limited [323,324,325]. A notable discovery is octopartenopin, a random-coiled pentapeptide from the suckers of *Octopus vulgaris* [326]. Cationic α-helical AMPs have been identified in *Octopus minor*, including octominins [327,328], octopromycin [329], and octoprohibitin [330]. Additionally, screening of the cuttlefish *Sepia officinalis* revealed peptides with antimicrobial potential: three (KT19, VA20, and GR21) identified by Houyvet et al. [331] and three others (NF19, AV19, and GK28) by Benoist et al. [332].

#### 4.7.4. Gastropoda AMPs

Several AMPs have been identified in aquatic snails. In marine snails, the α-helical peptide Cm-p1 and its derivatives obtained from *Cenchritis muricatus* exhibited potent antifungal activity without toxicity to mammalian cells [333,334]. Two proline-rich peptides from the invasive *Rapana venosa*, the random-coiled peptide 4 and the α-helical peptide 7, showed antibacterial activity against Gram-negative bacteria [335]. In freshwater snails, Bb-AMP4 from *Filopaludina bengalensis* displayed antibacterial effects against Gram-positive bacteria [336]. Additionally, two fragments of α-helical peptides, Pom-1 (Closticin 574) and Pom-2 (Cecropin D-like), identified in *Pomacea poeyana*, exhibited antibacterial activity, with Pom-1 also showing antiviral effects against ZIKV [337]. Moreover, the α-helical peptide Dolabellanin B2, isolated from the sea hare *Dolabella auricularia* and the sea slug *Peronia peronii*, displayed antifungal and antibacterial properties [338,339].

### 4.8. Other Phyla

Despite the extensive research on AMPs across many aquatic invertebrate phyla, certain groups remain significantly underrepresented in scientific literature, despite their ecological and evolutionary importance. Determining whether this limitation arises from a lack of studies or rather a genuine scarcity of AMPs is crucial. Four phyla are explored (Nematoda, Placozoa, Platyhelminthes, and Porifera) highlighting their known AMPs (Table S9) and addressing the challenges involved in studying these organisms.

#### 4.8.1. Nematoda

The phylum Nematoda comprises over 28,000 species of roundworms divided into two classes, Chromadorea and Enoplea [53,165]. While AMPs in the terrestrial model organism *Caenorhabditis elegans* are well-studied, few works have explored AMPs in aquatic nematodes [340]. A notable exception is the discovery of anisaxins, cecropin-like helical AMPs from the marine parasite *Anisakis*, the etiological agent of anisakiasis. These AMPs show potent bactericidal activity against Gram-negative bacteria with minimal cytotoxicity to human cells [341]. Recently, an integrated homology computational methodology was applied for the discovery of new helminth (both Nematoda and Platyhelminthes) AMPs. This approach identified thousands of putative AMPs and out of the selected few synthetized, four (nAMP-LP-18, nAMP-LP-104, nAMP-LP-249, and nAMP-LP-298) revealed antibacterial activity (<100 μg/mL) against at least one bacterial species [342].

#### 4.8.2. Placozoa

Placozoa is an ancient phylum of highly simplified multicellular organisms, considered among the most basal metazoans [343]. Although only four species are currently described, phylogenomic and gene content analyses suggest much greater diversity within the phylum [344,345]. Trichoplaxins, α-helical peptides from *Trichoplax adhaerens*, are the sole known AMP family in Placozoa, showing strong antimicrobial activity against bacteria and fungi. Additionally, the synthetic analog Trichoplaxin-2A shows minimal cytotoxicity to mammalian cells and high anticancer activity and selectivity [346,347].

#### 4.8.3. Platyhelminthes

Platyhelminthes, or flatworms, are a diverse phylum that includes parasitic species like *Schistosoma mansoni*, the causative agent of schistosomiasis, a disease affecting millions globally [165,348]. A peptide from *S. mansoni*, SmDLP, shares high homology with dermaseptin 3.1 from the frog *Agalychnis annae*. SmDLP demonstrates strong antimicrobial and hemolytic activities, which may help the parasite survive and evade the host immune system [349]. Additionally, an in silico study using an innovative algorithm and homology searches found that flatworms lack traditional AMPs, instead possessing a repertoire of unique, mostly cysteine-rich AMP-like peptides. Two novel AMP-like peptides, fAMP-LP-5 and fAMP-LP-17, showed antibacterial activity (<100 μg mL^−1^), highlighting their potential as novel antimicrobials [342].

#### 4.8.4. Porifera

The phylum Porifera, the oldest living metazoan group, comprises over 9300 species across four classes, with Demospongiae representing over 80% of sponge diversity [53]. Sponges form symbiotic relationships with microorganisms that produce chemical compounds previously thought synthesized by the sponges themselves [84]. Nearly half of all marine natural products discovered from invertebrates between 1990 and 2019 originated from sponges [350]. However, only one known AMP has been extracted from sponges: ASABF_SUBDO, a defensin-like cysteine-stabilized αβ motif from *Suberites domuncula*. Identified through EST database screening, ASABF_SUBDO shares high similarity with ASABF, a cysteine-rich AMP from terrestrial nematodes [117]. The ancient lineage of sponges may explain their advanced chemical defense mechanisms, with their broad peptide arsenal likely reducing the need for traditional short, linear AMPs. This suggests that sponges have evolved multiple defense strategies, including antimicrobial cyclic peptides [351,352,353].

### 4.9. “Non-Classical AMPs” Derived from Aquatic Invertebrates

**Figure 4:**
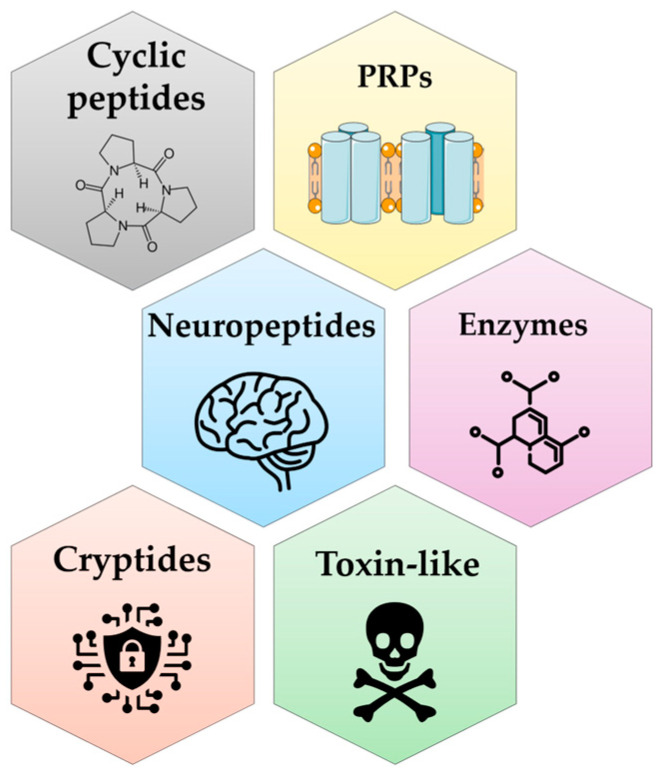
Key categories of “non-classical” AMPs.

While most AMPs derived from aquatic invertebrates are traditionally described as short, linear peptides composed of basic amino acids and directly involved in host defense, a broader category of “non-classical AMPs” is also found within these organisms. These molecules diverge significantly from the classical definition and demonstrate remarkable diversity in structure, origin, and function. This group encompasses a wide array of bioactive compounds, including cyclic peptides, antimicrobial enzymes, neuropeptides, pattern recognition proteins, and various cryptides with distinct origins (Figure 4, Table S10).

#### 4.9.1. Cyclic Peptides

Among several chemical modifications used to enhance peptide-based therapeutics, including N-methylation, PEGylation, glycosylation, lipidation, peptide stapling, and the incorporation of unnatural amino acids and disulfide bonds, cyclization of linear peptides is particularly promising [354,355,356,357,358]. Cyclization restricts peptide flexibility, improving bioavailability, stability, and specificity, while enhancing resistance to exopeptidase hydrolysis due to the absence of terminal ends, and protecting against endopeptidase cleavage with its rigid ring structure. These structural changes also improve membrane interactions, which may contribute to the enhanced antimicrobial properties by increasing the peptide’s ability to penetrate microbial membranes and maintain its functional conformation in the presence of environmental stressors such as changes in pH, temperature fluctuations, high salt concentrations, and the presence of proteases [359,360,361]. Cyclized peptides can be homodetic (with only peptide bonds) or heterodetic (containing non-peptide bonds like isopeptides, disulfides, or esters), with cyclic depsipeptides being a subtype that incorporates ester bonds by replacing amino acids with hydroxy acids [362,363]. Over 50 cyclic peptide drugs have been FDA-approved, making up nearly half of all approved peptide drugs, with many more in clinical trials [361,364]. However, only one originates from a marine organism: ziconotide, a synthetic version of ω-conotoxin MVIIA from the marine snail *Conus magus* [365]. Most antimicrobial cyclic peptides and depsipeptides from aquatic invertebrates have been isolated from marine sponges, with some exceptions from ascidians and sea slugs. These compounds primarily exhibit antibacterial activity, with some showing antifungal, antiviral, and antiparasitic properties. Although none have been approved, several, including Celebesides, Dolastatins, Homophymine A, Mirabamides, Theonellamides, and Theopapuamides, are being evaluated by pharmaceutical and nutraceutical industries [366].

#### 4.9.2. Antimicrobial Enzymes

Enzymes, primarily proteins or RNA molecules, act as biological catalysts to accelerate chemical reactions. In aquatic invertebrates, lysozymes and chitinases are key enzymes involved in innate immunity. Lysozymes, first identified by Alexander Fleming when he observed nasal mucus dissolving bacteria [367], hydrolyze glycosidic bonds between N-acetylglucosamine and N-acetylmuramic acid in bacterial peptidoglycans, resulting in cell lysis [368]. Beyond bacteriolysis, they enhance immune defenses by activating the complement pathway, promoting phagocytosis, and modulating immune responses, while also exhibiting anti-inflammatory, antitumor, and digestive functions [369]. Although they display antimicrobial activity, lysozymes are not classified as classical AMPs due to their enzymatic action. Based on their sequence, structure, and properties, lysozymes are categorized into three types: chicken-type (c-type), goose-type (g-type), and invertebrate-type (i-type) [370]. C-type lysozymes are common in both vertebrates and invertebrates, g-type lysozymes, initially discovered in goose egg whites, are also present in some invertebrates, and i-type lysozymes are primarily found in invertebrates. In aquatic invertebrates, i-type lysozymes are reported in Annelida, Arthropoda, Echinodermata, Mollusca, and Porifera [371,372,373,374,375], c-type lysozymes in Arthropoda and Mollusca [376,377], and g-type lysozymes in Arthropoda, Chordata, Mollusca, and Placozoa [378,379,380,381]. Chitinases, another class of immune-related enzymes, are glycosyl hydrolases that catalyze the degradation of chitin, a structural polysaccharide found in arthropod exoskeletons, fungal cell walls, and crustacean shells [382]. These enzymes are classified into two main families—GH18 and GH19—which differ significantly in their catalytic mechanisms and structural features. GH18 chitinases typically require two catalytic residues: a glutamic acid to break down the chitin substrate and an acidic residue that stabilizes the reaction. In contrast, GH19 chitinases rely on a single catalytic residue, usually glutamate, for their activity. The active site and overall structure of GH19 chitinases, which are characterized by a higher content in α-helices, contrast with the triose-phosphate isomerase (TIM) barrel fold seen in GH18 enzymes. Additionally, GH19 chitinases exhibit higher specificity for chitin and enhanced stability under acidic conditions, while GH18 chitinases are more versatile, and capable of degrading both chitin and peptidoglycan. Beyond their direct antimicrobial activity, chitinases play important roles in immune regulation, tissue remodeling, and wound healing [383]. In aquatic invertebrates, the antimicrobial activity of chitinases has been reported only in arthropods, particularly prawns and crabs [384,385,386].

#### 4.9.3. Neuropeptides

Neuropeptides, highly conserved peptide-based neuromodulators released by neurons, regulate physiological processes, neural signaling, and behavioral plasticity, with some also displaying antimicrobial activity. Among these, tachykinin-related peptides (TRPs) are a prominent neuropeptide family characterized by a conserved C-terminal region. They have been identified in various aquatic invertebrates, including arthropods [387], mollusks [44], and annelids [388]. Notably, urechistachykinins, TRPs isolated from the echiuroid worm *Urechis unicinctus*, exhibit broad-spectrum antimicrobial activity against bacteria and fungi without cytotoxicity to human erythrocytes [388]. In addition, other neuropeptides with antimicrobial properties include peptide B, derived from proenkephalin A in the leech *T. tessulatum*, and nda-1, hym-357, hym-370, and rfamide III, secreted by sensory and ganglion neurons in the ectodermal epithelium of *Hydra* [389].

#### 4.9.4. Pattern Recognition Proteins

The innate immune response relies on the recognition of pathogen-associated molecular patterns (PAMPs) by pattern recognition proteins (PRPs). PAMPs are conserved pathogen structures, such as LPS, β-1,3-glucans, and peptidoglycans, which trigger immune responses including phagocytosis, encapsulation, proteinase cascades, and AMP synthesis [390,391,392]. PRPs include Toll-like receptors (TLRs), lectins, peptidoglycan recognition proteins (PGRPs), lipopolysaccharide-binding proteins (LBPs), bactericidal permeability-increasing proteins (BPIs), β-glucan binding proteins (βGBPs), and lipopolysaccharide- and β-1,3-glucan-binding proteins (LGBPs). TLRs are the most extensively studied in aquatic invertebrates. They play a crucial role in both innate and adaptive immunity by recognizing diverse PAMPs, including bacterial DNA, LPS, mannose, and peptidoglycans [393,394]. TLRs have been functionally characterized in numerous aquatic invertebrate phyla, such as Annelida [395], Arthropoda [396], Chordata [397], Echinodermata [398], and Mollusca [399]. Although the functional roles of LBPs and BPIs in invertebrates are unclear, these LBP/BPI proteins have been identified in Echinodermata [400], Mollusca [401], and Nematoda [402]. Similarly, LGBPs, which bind both LPS and β-1,3-glucans, have been characterized in Arthropoda [403] and Mollusca [404]. PRPs have also inspired AMPs’ development, such as HDH-LGBP-A1 and HDH-LGBP-A2, derived from the polysaccharide-binding motif of *Haliotis discus hannai* LGBP. These AMPs show antibacterial, antifungal, and anticancer activities with minimal toxicity to human cells [405].

#### 4.9.5. Cryptides from Regulatory Proteins

Cryptides are bioactive peptides derived from larger parent proteins or precursor peptides that serve a primary function. These cryptides are “hidden” within the parent protein, become functional once cleaved, and often exhibit activities distinct from their parent protein’s primary role, such as immunomodulatory, hormone-like, or antimicrobial [406]. Histones, essential proteins for DNA packaging in eukaryotic cells and regulating gene expression, also hide cryptides with antimicrobial properties such as Buforin I derived from histone H2A in *Bufo gargarizans* [407]. In aquatic invertebrates, several histone-derived cryptides with antibacterial and antifungal effects have been reported in Arthropoda [408] and Mollusca [409]. Ubiquitin, a small, highly conserved protein found in all eukaryotes is classified into two groups: type-I and type-II ubiquitin-like proteins. Type-I ubiquitin contains a conserved Gly-Gly sequence at its C-terminus, enabling cleavage and attachment to target proteins through a post-translational modification (PTM) known as ubiquitination, which commonly leads to protein degradation, protein activity regulation, or removal of damaged proteins [410]. In contrast, type-II ubiquitin-like proteins lack the glycine motif and do not undergo cleavage; instead, they regulate protein interactions and cellular pathways [411]. Ubiquitin also plays roles in DNA repair, cell signaling, and immune response [412]. In aquatic invertebrates, ubiquitin-derived cryptides with antimicrobial activities have been found in Mollusca, including cgUbiquitin from *C. gigas* [413], and RpUbi from *R. philippinarum* [414]. Additionally, paracentrin, a cationic cryptide derived from a β-thymosin of *Paracentrotus lividus* displays antibacterial and antibiofilm properties [415,416]. There are also synthetic peptides designed based on portions of proteins, such as schistocins. Their design was inspired by the C-terminal of *S. mansoni* Kunitz Inhibitor SmKI-1. Synthetized schistocins undergo a membrane-induced conformational change from random coil to α-helix and their antimicrobial activities are variable with some having interesting effects against bacteria and fungi [417].

#### 4.9.6. Cryptides from Respiratory Proteins

Respiratory proteins primarily function to bind and transport oxygen, but some cryptides derived from them also contribute to immune responses. Hemerythrin, an ancient non-heme oxygen-binding protein family, is found across all three domains of life and is most diverse in Annelida [418,419,420,421]. In aquatic invertebrates, the cryptide MsHemerycin, derived from the N-terminus of hemerythrin, was isolated from the lugworm *Marphysa sanguinea*. This peptide, characterized by N-terminal acetylation and an unordered structure, demonstrates unique mechanisms of action and shows antimicrobial activity against *B. subtilis* [422]. Hemocyanin, a binuclear type 3 copper protein responsible for oxygen transport in the hemolymph of many arthropods and mollusks, is also a source of cryptides with antimicrobial properties [423]. Cryptides derived from C-terminal fragments of shrimp hemocyanin, including PsHCt1, PsHCt2, PvHCt, FCHc-C1, and FCHc-C2, exhibit diverse antimicrobial activities [424,425]. Additionally, haliotisin, a cryptide from the functional unit E of abalone *Haliotis tuberculata* hemocyanin, has been used as a template for synthetic peptides with demonstrated antibacterial properties [426]. Furthermore, five synthetic peptides (L1, L2, L8, L10, and L12) predicted from the large subunit of *P. vannamei* hemocyanin also show antibacterial effects [427].

#### 4.9.7. Cryptides from Ribosomal Proteins

Ribosomal proteins, traditionally known for their role in protein synthesis, can also generate cryptides involved in host defense. In aquatic invertebrates, CgRPL29, a cryptide derived from the *C. gigas* 60S ribosomal protein L29, has a predicted unordered, non-amphipathic structure with two partial α-helical regions and demonstrates antibacterial activity [428]. Additionally, BjRPS23, a cryptide from the *B. japonicum* ribosomal protein RPS23, functions as both a pattern recognition receptor and an antimicrobial effector. It interacts with bacterial membranes and promotes the generation of reactive oxygen species within bacterial cells, leading to their death [429].

#### 4.9.8. Toxin-Like Proteins

Toxin-like proteins play a vital role in prey capture and defense in various organisms, especially in cnidarians. These proteins, released through specialized cells called nematocysts, immobilize or kill prey by disrupting cellular membranes, interfering with signaling pathways, or causing tissue damage, facilitating prey capture. In defense, they deter predators by inducing pain, inflammation, or paralysis, thus reducing predation risk. Evolutionarily, these proteins are optimized to balance prey acquisition and protection against a variety of ecological threats, ensuring survival in competitive marine environments [430].

Beyond their role in prey capture and defense, toxin-like proteins have evolved to target and neutralize microbial pathogens [431]. Cytolysins from the sea anemone *Stichodactyla helianthus*, sticolisins, demonstrate antiparasitic activity against *Giardia duodenalis* with minimal toxicity to human erythrocytes [432]. Neurotoxin 2 from *Anemonia sulcata* demonstrates antibacterial effects against Gram-positive *M. luteus* [433], while the cysteine-rich Ueq 12-1 from *Urticina eques* displays moderate activity against Gram-positive bacteria and enhances transient receptor potential ankyrin 1 channel activity [99]. Additionally, transcriptomic analysis of *Epiactis japonicus* tentacles identified toxin-like AMPs, some of which display weak antibacterial activity [434].

## 5. Applications of Aquatic Invertebrate AMPs in Aquaculture

The intensification of aquaculture has led to increased stress on farmed species, weakening their immune systems and leading to the overuse of antibiotics. This overuse has exacerbated the rise of AMR, posing significant risks to both aquaculture and public health [4]. Effective strategies to mitigate AMR include adopting good aquaculture and biosecurity practices, implementing disease prevention measures, and minimizing antibiotic use by exploring alternative solutions [435,436]. Research has identified multiple antibiotic-resistance genes in aquaculture, including those related to β-lactams, tetracyclines, macrolides, quinolones, and sulfonamides [437]. To combat AMR, global regulations should enforce stricter controls on antibiotic use and ban commonly misused antibiotics, following the good examples of FDA-banned nitrofurans and chloramphenicol [435]. Currently, only four antibiotics are FDA-approved for aquaculture use, namely florfenicol, oxytetracycline, sulfadimethoxine/ormetoprim, and sulfamerazine [438]. Several alternatives to antibiotics have been explored, including vaccines, probiotics, prebiotics, synbiotics, bacteriophages, chicken egg yolk immunoglobulin (IgY), medicinal plants, bacteriocins, and AMPs [437]. AMPs are particularly promising due to their immediate and direct antimicrobial action, broad-spectrum activity against diverse pathogens (Gram-positive and Gram-negative bacteria, viruses, fungi, and parasites), and low risk of AMR development. Additionally, some AMPs possess immunomodulatory properties that enhance the host’s innate immunity. AMPs offer practical advantages, including relatively low production costs, easy and long storage in bulk, and rapid availability after an infection [439]. In aquaculture, AMPs have been studied as feed additives and therapeutic drugs and through genetic engineering, provide a potential alternative to antibiotics and support the health and productivity of farmed species. However, their application faces several challenges, including the high cost and difficulty of scaling up production, as natural extraction often yields low quantities. AMPs are susceptible to degradation by proteases, and their stability can be affected by environmental factors such as pH, salinity, and temperature. Moreover, some AMPs may exhibit cytotoxicity at higher concentrations and may potentially impact non-target organisms. Delivering AMPs effectively in aquatic environments is challenging due to dilution and dispersion, necessitating optimized delivery systems. Furthermore, stringent regulatory requirements for safety and efficacy must be met before AMPs can be approved for use in aquaculture [440].

### 5.1. AMPs as Aquaculture Feed Additives and Therapeutic Drugs

Several studies have explored the use of AMPs as dietary supplements in aquaculture, focusing primarily on fish, but also extending to shrimp and shellfish [441,442,443]. Wang et al. [444] analyzed the literature results on the application of AMPs as dietary supplements and concluded that AMPs can significantly enhance growth performance, enzymatic activity, and disease resistance of cultured species. Furthermore, although research on this topic is still limited, AMPs have also demonstrated a positive impact on the gut microbiomes of fish. However, none of the AMPs reviewed were derived from aquatic invertebrates. Studies on the application of aquatic invertebrates’ AMPs as feed additives are indeed very scarce. The AMP octopromycin derived from the proline-rich protein 5 of *O. minor* was in vivo tested in zebrafish infected with *A. baumannii* and showed to cause a significant survival increase in fish [329]. Additionally, 6His-tatritin, derived from the AMP tatritin with six added histidines, revealed an improvement in immune responses and intestinal microbiota in the grass carp *Ctenopharyngodon idellus* challenged with *A. hydrophila* [196].

### 5.2. Genetic Engineering and Transgenesis of AMP Genes in Fish

CRISPR/Cas9 gene editing is a powerful tool for enhancing fish immunity by introducing vector-engineered antimicrobial peptide genes and other immune-related genes. Studies reviewed by Wang and Cheng [445] show that AMP gene transgenesis in aquaculture fish reduces bacterial loads, improves survival rates after infection, and modulates the expression of immune and AMP genes. AMP genes often work synergistically with other immune-related genes to strengthen defenses, though their effectiveness varies by fish species, pathogen targeted, and the AMP gene used. While highly effective against bacterial infections, immune defense by AMP gene transgenesis against viral and parasitic infections is limited. Additionally, CRISPR/Cas9-based transgenesis can also combine immune improvements with trait enhancements like rapid growth, sterility, and improved fatty acid profiles. While CRISPR/Cas9 technology holds great promise for introducing AMP genes into farmed species, its practical application faces several challenges. These include the high costs of gene editing, difficulties in scaling these methods for commercial use, limited genomic knowledge of many farmed species, challenges in achieving stable AMP gene expression across generations, and significant regulatory and ethical concerns. Currently, there are no works on the application of aquatic invertebrates AMPs gene transgenesis, and further work should be done to explore the significant potential CRISPR/Cas9 gene editing has to offer for sustainable and efficient aquaculture production.

## 6. Conclusions

The rapid intensification of aquaculture has resulted in higher population densities of farmed species, increasing their vulnerability to disease outbreaks, while the overreliance on traditional antibiotics has exacerbated the global AMR crisis. This underscores the urgent need for alternative strategies to manage pathogens and ensure the sustainability of aquaculture systems, with AMPs emerging as a promising solution. As natural components of the humoral defense systems of many organisms, AMPs play a critical role in protecting hosts from pathogenic threats. These molecules possess several unique advantages, including immediate and direct antimicrobial action, broad-spectrum activity against bacteria, viruses, fungi, and parasites, and a low risk of promoting AMR. Additionally, AMPs exhibit additional bioactivities such as immunomodulatory, anticancer, and antifouling properties, making them versatile tools in biotechnology. Practical benefits of AMPs include cost-effective production, long-term bulk storage, and rapid availability for deployment during infections.

Marine-derived AMPs are especially promising due to evolutionary adaptations to high-salinity environments, resulting in improved stability, enhanced electrostatic interactions, and a broader antimicrobial spectrum—traits that are particularly suited for aquaculture applications, where salinity fluctuations are common. Despite their potential, research on AMPs from aquatic invertebrates, particularly from phyla like Cnidaria, Platyhelminthes, and Porifera, remains underdeveloped. Existing knowledge is fragmented, with limited information in AMP databases, and dispersed in literature. Around 350 AMPs from 10 invertebrate phyla have been identified, with some demonstrating promising in vitro results, including activity against 85 pathogens relevant to aquaculture. However, the lack of in vivo studies hinders the application of these peptides in aquaculture. Addressing this gap could open new opportunities for using AMPs from aquatic invertebrates in aquaculture, including their applications as feed additives, therapeutic agents, and in genetic engineering approaches, such as the transgenesis of AMP genes, in line with recent successful efforts involving AMPs from other sources.

The application of AMPs in aquaculture faces several technical challenges, including efficacy, availability, and stability in aquatic environments, as well as ecological concerns, such as potential impacts on non-target organisms. To overcome these hurdles, the development of peptidomimetics—synthetically designed molecules inspired by natural AMPs—presents a promising alternative [446,447]. However, challenges such as production scalability, regulatory compliance, and other ecological impacts remain. To fully exploit the potential of AMPs, future research should focus on expanding the understanding of their natural diversity, particularly regarding their structures, mechanisms of action, and bioactivities. Comprehensive in vivo assessments are essential to evaluate their efficacy and safety as feed additives and therapeutic agents in relevant aquaculture species. Furthermore, advances in genetic engineering, particularly CRISPR/Cas9-mediated transgenesis of AMP genes into fish genomes, hold significant promise for developing enhanced aquaculture species. These advancements can strengthen the immune system of farmed species while also improving key physical and compositional traits, making them more resilient and ultimately a better aquaculture product. Exploring the biotechnological potential of AMPs can revolutionize aquaculture, fostering sustainability and resilience, safeguarding ecosystem health and public well-being, and curbing antibiotic dependency while tackling the escalating threat of AMR.

## Data Availability

The original contributions presented in this study are included in the Supplementary Material. Further inquiries can be directed to the author(s).

## Supplementary Materials

The following supporting information can be downloaded at: https://www.mdpi.com/article/10.3390/microorganisms13010156/s1: Table S1: Pathogenicity profiles of the microorganisms targeted by AMPs derived from aquatic invertebrates; Table S2: Database of the AMPs derived from aquatic invertebrates; Table S3: Antimicrobial activities of AMPs from aquatic invertebrate annelids against pathogens relevant to aquaculture and human health; Table S4: Antimicrobial activities of AMPs from aquatic invertebrate arthropods against pathogens relevant to aquaculture and human health; Table S5: Antimicrobial activities of AMPs from aquatic invertebrate chordates against pathogens relevant to aquaculture and human health; Table S6: Antimicrobial activities of AMPs from cnidaria against pathogens relevant to aquaculture and human health; Table S7: Antimicrobial activities of AMPs from echinoderms against pathogens relevant to aquaculture and human health; Table S8: Antimicrobial activity of AMPs from mollusks against pathogens relevant to aquaculture and human health; Table S9: Antimicrobial activity of AMPs from other invertebrates against microorganisms including pathogens relevant to aquaculture and human health; Table S10: Activity of “non-classical AMPs” derived from aquatic invertebrates against pathogens relevant to aquaculture and human health.
