# Supplementary material for: Aquatic Invertebrate Antimicrobial Peptides in the Fight Against Aquaculture Pathogens
Source: Microorganisms. 2025 Jan 14;13(1):156. doi: 10.3390/microorganisms13010156 (PMC11767717; doi:10.3390/microorganisms13010156)
Supplement: Supplementary file 1 [file microorganisms-13-00156-s001.zip › Supplementary_Material.pdf]

Supplementary Material

Aquatic Invertebrate Antimicrobial Peptides in the Fight Against Aquaculture Pathogens

Tomás Rodrigues [1,2], Francisco Antonio Guardiola [3], Daniela Almeida [4] and Agostinho Antunes [1,2,*]

Index

Table S1. Pathogenicity profiles of the microorganisms targeted by AMPs derived from aquatic invertebrates. 2

Table S2. Database of the AMPs obtained from aquatic invertebrates. (Please check excel file "TableS2") 4

Table S3. Antimicrobial activities of AMPs from aquatic invertebrate annelids against pathogens relevant to aquaculture and human health (Pathogens specific to aquaculture species are shown in bold; those specific to humans are in italic; pathogens affecting both are in bold italic. Non-pathogenic bacteria are shown in regular text). 4

Table S4. Antimicrobial activities of AMPs from aquatic invertebrate arthropods against pathogens relevant to aquaculture and human health (Pathogens specific to aquaculture species are shown in bold; those specific to humans are in italic; pathogens affecting both are in bold italic. Non-pathogenic bacteria are shown in regular text). 7

Table S5. Antimicrobial activities of AMPs from aquatic invertebrate chordates against pathogens relevant to aquaculture and human health (Pathogens specific to aquaculture species are shown in bold; those specific to humans are in italic; pathogens affecting both are in bold italic. Non-pathogenic bacteria are shown in regular text). 15

Table S6. Antimicrobial activities of AMPs from cnidaria against pathogens relevant to aquaculture and human health (Pathogens specific to aquaculture species are shown in bold; those specific to humans are in italic; pathogens affecting both are in bold italic. Non-pathogenic bacteria are shown in regular text). 20

Table S7. Antimicrobial activities of AMPs from echinoderms against pathogens relevant to aquaculture and human health (Pathogens specific to aquaculture species are shown in bold; those specific to humans are in italic; pathogens affecting both are in bold italic. Non-pathogenic bacteria are shown in regular text). 21

Table S8. Antimicrobial activity of AMPs from mollusks against pathogens relevant to aquaculture and human health (Pathogens specific to aquaculture species are shown in bold; those specific to humans are in italic; pathogens affecting both are in bold italic. Non-pathogenic bacteria are shown in regular text). 22

Table S9. Antimicrobial activity of AMPs from other invertebrates against microorganisms including pathogens relevant to aquaculture and human health (Pathogens specific to aquaculture species are shown in bold; those specific to humans are in italic; pathogens affecting both are in bold italic. Non-pathogenic bacteria are shown in regular text). 28

Table S10. Activity of "non classical AMPs" derived from aquatic invertebrates against pathogens relevant to aquaculture and human health (Pathogens specific to aquaculture species are shown in bold; those specific to humans are in italic; pathogens affecting both are in bold italic. Non-pathogenic bacteria are shown in regular text). 29

Supplementary Material References 30

Table S1. Pathogenicity profiles of the microorganisms targeted by AMPs derived from aquatic invertebrates.

Type	Genus	Species	Aquaculture pathogen	Human pathogen	References
Fungi	Aspergillus	fumigatus	Yes	Yes	[1,2]
Fungi	Aspergillus	niger	Yes	Yes	[3,4]
Fungi	Aspergillus	ochraceus	Yes	Yes	[5,6]
Fungi	Aspergillus	terreus	Yes	Yes	[7,8]
Fungi	Candida	albicans	Yes	Yes	[9,10]
Fungi	Candida	parapsilosis	Yes	Yes	[5,11]
Fungi	Candida	tropicalis	Yes	Yes	[12,11]
Fungi	Cladosporium	sp.	Yes	Yes	[13,14]
Fungi	Fusarium	oxysporum	Yes	Yes	[15,16]
Fungi	Fusarium	solani	Yes	Yes	[17,18]
Gram-	Acinetobacter	baumannii	Yes	Yes	[19,20]
Gram-	Aeromonas	hydrophila	Yes	Yes	[21,22]
Gram-	Citrobacter	freundii	Yes	Yes	[23,24]
Gram-	Edwardsiella	tarda	Yes	Yes	[25,26]
Gram-	Enterobacter	cloacae	Yes	Yes	[27,28]
Gram-	Escherichia	coli	Yes	Yes	[29,30]
Gram-	Klebsiella	aerogenes	Yes	Yes	[31,32]
Gram-	Klebsiella	oxytoca	Yes	Yes	[33,34]
Gram-	Klebsiella	pneumoniae	Yes	Yes	[35,36]
Gram-	Photobacterium	damselae	Yes	Yes	[37]
Gram-	Proteus	mirabilis	Yes	Yes	[38,39]
Gram-	Proteus	vulgaris	Yes	Yes	[40,41]
Gram-	Pseudomonas	aeruginosa	Yes	Yes	[42,43]
Gram-	Pseudomonas	putida	Yes	Yes	[44,45]
Gram-	Pseudomonas	stutzeri	Yes	Yes	[46,47]
Gram-	Salmonella	choleraesuis	Yes	Yes	[48,49]
Gram-	Salmonella	enterica	Yes	Yes	[50,51]
Gram-	Salmonella	typhimurium	Yes	Yes	[52,53]
Gram-	Serratia	marcescens	Yes	Yes	[54,55]
Gram-	Shewanella	algae	Yes	Yes	[56,57]
Gram-	Shigella	flexneri	Yes	Yes	[58,59]
Gram-	Vibrio	alginolyticus	Yes	Yes	[60,61]
Gram-	Vibrio	anguillarum	Yes	Yes	[62,63]
Gram-	Vibrio	cholerae	Yes	Yes	[64,65]
Gram-	Vibrio	fluvialis	Yes	Yes	[66,67]
Gram-	Vibrio	harveyi	Yes	Yes	[68,69]
Gram-	Vibrio	parahaemolyticus	Yes	Yes	[70,71]
Gram-	Vibrio	vulnificus	Yes	Yes	[72,73]
Gram-	Yersinia	enterocolitica	Yes	Yes	[74,75]
Gram+	Aerococcus	viridans	Yes	Yes	[76,77]
Gram+	Bacillus	cereus	Yes	Yes	[78,79]
Gram+	Bacillus	mycoides	Yes	Yes	[80,81]
Gram+	Bacillus	subtilis	Yes	Yes	[82,83]
Gram+	Clostridium	perfringens	Yes	Yes	[84,85]
Gram+	Enterococcus	faecalis	Yes	Yes	[86,87]
Gram+	Enterococcus	faecium	Yes	Yes	[88,89]
Gram+	Kocuria	rhizophila	Yes	Yes	[90,91]
Gram+	Lactococcus	garvieae	Yes	Yes	[92,93]
Gram+	Lactococcus	lactis	Yes	Yes	[94,95]
Gram+	Micrococcus	luteus	Yes	Yes	[90,96]
Gram+	Mycobacterium	smegmatis	Yes	Yes	[97,98]
Gram+	Rhodococcus	sp.	Yes	Yes	[99,100]
Gram+	Staphylococcus	aureus	Yes	Yes	[101,102]
Gram+	Staphylococcus	epidermidis	Yes	Yes	[103,104]
Gram+	Staphylococcus	haemolyticus	Yes	Yes	[105,106]
Gram+	Staphylococcus	saprophyticus	Yes	Yes	[107,108]
Gram+	Streptococcus	agalactiae	Yes	Yes	[109,110]
Gram+	Streptococcus	iniae	Yes	Yes	[109,111]
Gram+	Streptococcus	parauberis	Yes	Yes	[112,113]
Gram+	Streptococcus	suis	Yes	Yes	[114,115]
Fungi	Saprolegnia	parasitica	Yes	No	[116]
Gram-	Aeromonas	salmonicida	Yes	No	[117]
Gram-	Aliivibrio	fischeri	Yes	No	[118]
Gram-	Aliivibrio	logei	Yes	No	[119]
Gram-	Edwardsiella	piscicida	Yes	No	[25]
Gram-	Pseudomonas	fluorescens	Yes	No	[120]
Gram-	Vibrio	aestuarianus	Yes	No	[121]
Gram-	Vibrio	bivalvicida	Yes	No	[122]
Gram-	Vibrio	campbellii	Yes	No	[123]
Gram-	Vibrio	coralliilyticus	Yes	No	[124]
Gram-	Vibrio	ichthyoenteri	Yes	No	[125]
Gram-	Vibrio	nigripulchritudo	Yes	No	[126]
Gram-	Vibrio	ordalii	Yes	No	[127]
Gram-	Vibrio	owensii	Yes	No	[128]
Gram-	Vibrio	penaeicida	Yes	No	[129]
Gram-	Vibrio	splendidus	Yes	No	[130]
Gram-	Vibrio	tapetis	Yes	No	[131]

Gram-	Vibrio	tasmaniensis	Yes	No	[132]
Gram-	Yersinia	ruckeri	Yes	No	[133]
Gram+	Carnobacterium	maltaromaticum	Yes	No	[134]
Gram+	Kurthia	gibsonii	Yes	No	[135]
Gram+	Planococcus	citreus	Yes	No	[52]
Parasite	Scuticociliatia		Yes	No	[136]
Virus	OsHV-1		Yes	No	[137]
Virus	WSSV		Yes	No	[138]
Fungi	Aureobasidium	pullulans	No	Yes	[139]
Fungi	Candida	auris	No	Yes	[140]
Fungi	Clavispora	lusitaniae	No	Yes	[141]
Fungi	Cryptococcus	neoformans	No	Yes	[142]
Fungi	Geotrichum	candidum	No	Yes	[143]
Fungi	Nakaseomyces	glabratus	No	Yes	[144]
Fungi	Pichia	inconspicua	No	Yes	[145]
Fungi	Pichia	kudriavzevii	No	Yes	[146]
Fungi	Rhizopus	arrhizus	No	Yes	[147]
Fungi	Rhodotorula	sp.	No	Yes	[148]
Fungi	Saccharomyces	cerevisiae	No	Yes	[149]
Fungi	Trichoderma	viride	No	Yes	[150]
Fungi	Trichophyton	mentagrophytes	No	Yes	[151]
Fungi	Trichophyton	rubrum	No	Yes	[152]
Gram-	Haemophilus	influenzae	No	Yes	[153]
Gram-	Neisseria	gonorrhoeae	No	Yes	[154]
Gram-	Providencia	stuartii	No	Yes	[155]
Gram-	Salmonella	enteritidis	No	Yes	[156]
Gram-	Salmonella	newport	No	Yes	[157]
Gram-	Shigella	sonnei	No	Yes	[158]
Gram+	Arthrobacter	sp.	No	Yes	[159]
Gram+	Bacillus	licheniformis	No	Yes	[160]
Gram+	Bacillus	megaterium	No	Yes	[161]
Gram+	Bacillus	thuringiensis	No	Yes	[162]
Gram+	Corynebacterium	stationis	No	Yes	[163]
Gram+	Cutibacterium	acnes	No	Yes	[164]
Gram+	Dermacoccus	nishinomiyaensis	No	Yes	[165]
Gram+	Exiguobacterium	sp.	No	Yes	[166]
Gram+	Listeria	ivanovii	No	Yes	[167]
Gram+	Listeria	monocytogenes	No	Yes	[168]
Gram+	Microbacterium	maritypicum	No	Yes	[169]
Gram+	Micrococcus	tetragenus	No	Yes	[170]
Gram+	Mycobacterium	phlei	No	Yes	[171]
Gram+	Streptococcus	mutans	No	Yes	[172]
Gram+	Streptococcus	pyogenes	No	Yes	[173]
Gram+	Streptococcus	vestibularis	No	Yes	[174]
Parasite	Leishmania	donovani	No	Yes	[175]
Parasite	Nippostrongylus	brasiliensis	No	Yes	[176]
Parasite	Trichomonas	vaginalis	No	Yes	[177]
Parasite	Trypanosoma	cruzi	No	Yes	[178]
Virus	HIV-1		No	Yes	[179]
Virus	HSV-1		No	Yes	[180]
Virus	HSV-2		No	Yes	[181]
Virus	SARS-CoV-2		No	Yes	[182]
Virus	ZIKV		No	Yes	[183]
Fungi	Malassezia	furfur	No	Yes	[184,185]
Fungi	Alternaria	brassicicola	No	No	
Fungi	Botrytis	cinerea	No	No	
Fungi	Colletotrichum	orbiculare	No	No	
Fungi	Cordyceps	tenuipes	No	No	
Fungi	Fusarium	culmorum	No	No	
Fungi	Fusarium	graminearum	No	No	
Fungi	Komagataella	pastoris	No	No	
Fungi	Neocosmospora	haematococca	No	No	
Fungi	Neofabraea	vagabunda	No	No	
Fungi	Neurospora	crassa	No	No	
Fungi	Parastagonospora	nodorum	No	No	
Fungi	Penicillium	crustosum	No	No	
Fungi	Penicillium	roqueforti	No	No	
Fungi	Pyricularia	grisea	No	No	
Fungi	Rhizopus	stolonifer	No	No	
Fungi	Trichoderma	beigelii	No	No	
Fungi	Verticillium	dahliae	No	No	
Fungi	Yarrowia	lipolytica	No	No	
Gram-	Oceanisphaera	donghaensis	No	No	
Gram-	Pectobacterium	carotovorum	No	No	
Gram-	Pseudoalteromonas	carrageenovora	No	No	
Gram-	Salmonella	minnesota	No	No	
Gram-	Salmonella	pullorum	No	No	
Gram-	Vibrio	diabolicus	No	No	
Gram+	Bifidobacterium	bifidum	No	No	
Gram+	Carnobacterium	divergens	No	No	

Gram+	Carnobacterium	funditum	No	No
Gram+	Carnobacterium	mobile	No	No
Gram+	Corynebacterium	glutamicum	No	No
Gram+	Peribacillus	simplex	No	No

Table S2. Database of the AMPs obtained from aquatic invertebrates. (Please check excel file "TableS2")

Table S3. Antimicrobial activities of AMPs from aquatic invertebrate annelids against pathogens relevant to aquaculture and human health (Pathogens specific to aquaculture species are shown in bold; those specific to humans are in italic; pathogens affecting both are in bold italic. Non-pathogenic bacteria are shown in regular text).

Protein Family	Compound	Species	Class	Microorganism	MIC	MLC	Ref.
Class Clitellata							
Lumbricin	Hm-lumbricin	*H. medicinalis*	Gram+	*D. nishinomiyaensis*	NV		[186]
Macin	Hirudomacin	*H. nipponica*	Gram+	***B. subtilis***		0.39 μg mL^{-1}	[187]
				L. monocytogenes		6.25 μg mL^{-1}	
				S. aureus		1.56 μg mL^{-1}	
			Gram-	***E. coli***		6.25 μg mL^{-1}	
	Neuromacin	*H. medicinalis*	Gram+	*D. nishinomiyaensis*	1.95–3.8 μM	7.8–15.6 μM	[186]
			Gram+	*B. megaterium*		0.2 μg mL^{-1}	[188]
				S. aureus		6.25 μg mL^{-1}	
			Gram-	***E. coli***		25 μg mL^{-1}	
	TtTheromacin	*T. tessulatum*	Gram+	***M. luteus***	0.0165–0.033 μM	0.26–0.52 μM	[189]
			Gram+	*B. megaterium*		0.39 μg mL^{-1}	[188]
				S. aureus		100 μg mL^{-1}	
			Gram-	***E. coli***		25 μg mL^{-1}	
Theromyzin	Theromyzin	*T. tessulatum*	Gram+	***M. luteus***	0.25–0.5 μM		[189]
Class Polychaeta							
BRICHOS-AMPs	AA139	*A. marina*	Gram-	***E. coli***	0.125 μg mL^{-1}		[190]
				K. pneumoniae	1 μg mL^{-1}		
			Gram+	*B. licheniformis*	8 μM		[191]
				B. subtilis	8 μM		
				S. aureus	1 μM		
			Gram-	***A. baumannii***	0.125 μM		
				E. cloacae	1 μM		
				E. coli	0.25 μM		
				K. pneumoniae	2 μM		
				P. aeruginosa	0.25-1 μM		
				P. mirabilis	1 μM		
	Abarenicin-1	*A. pacifica*	Gram+	*B. licheniformis*	4 μM		[191]
				B. subtilis	2 μM		
				S. aureus	2 μM		
			Gram-	***A. baumannii***	0.125 μM		
				E. cloacae	2 μM		
				E. coli	0.25-0.5 μM		
				K. pneumoniae	2 μM		
				P. aeruginosa	0.25/0.5 μM		
				P. mirabilis	0.5 μM		
	Alvinellacin	*A. pompejana*	Gram+	*B. megaterium*	0.012–0.024 μM	0.024 μM	[192]
				S. aureus	0.048–0.096 μM	>0.0.19 μM	
			Gram-	***E. coli***	0.012–0.024 μM	0.048 μM	[192]
				Pseudomonas sp.	0.001–0.003 μM	0.012 μM	
				V. diabolicus	0.048–0.096 μM	>0.0.19 μM	
			Gram-	O. donghaensis	0.625 μM	1.25 μM	[193]
				Pseudomonas sp.	0.07 μM	0.31 μM	
				V. alginolyticus	0.31 μM	0.625 μM	
				V. diabolicus	2.5 μM	20 μM	
				V. fluvialis	0.625 μM	10 μM	
	AmBRI-44a	*A. marina*	Gram+	*B. licheniformis*	0.25-1 μM		[194]
				B. mycoides	8 μM		
				B. subtilis	0.25-2 μM		
				M. phlei	0.5-4 μM		
				M. luteus	4-32 μM		
			Fungi	***C. albicans***	16 μM		
	Arenicin-1	*A. marina*	Gram+	*L. monocytogenes*	0.6 μg mL^{-1}		[195]
				S. aureus	2 μM		
				S. epidermidis	4 μM		
			Gram-	***E. coli***	4 μg mL^{-1}		
			Fungi	***C. albicans***	4.5 μg mL^{-1}		
			Gram-	***E. coli***	8 μM		[196]
				P. aeruginosa	2 μM		
			Gram-	O. donghaensis	0.15 μM	0.625 μM	[193]
				Pseudomonas sp.	0.15 μM	1.25 μM	
				S. algae	40 μM	>40 μM	
				V. alginolyticus	0.15 μM	0.31 μM	
				V. diabolicus	5 μM	20 μM	
				V. fluvialis	0.31 μM	1.25 μM	
	Arenicin-1-S	*A. marina*	Gram+	***S. aureus***	4 μM		[196]
				S. epidermidis	8 μM		
			Gram-	***E. coli***	16 μM		
				P. aeruginosa	8 μM		

Arenicin-2	*A. marina*	Gram+	*B. megaterium*	2.6 μg mL^{-1}	[195]
			L. monocytogenes	0.6 μg mL^{-1}	
			M. luteus	2.6 μg mL^{-1}	
		Gram-	***E. coli***	4 μg mL^{-1}	
Arenicin-3	*A. marina*	Gram+	***B. subtilis***	8 μg mL^{-1}	[197]
			S. aureus	64 μg mL^{-1}	
		Gram-	***A. baumannii***	0.5 μg mL^{-1}	
			E. coli	0.125-0.5 μg mL^{-1}	
			K. pneumoniae	1-4 μg mL^{-1}	
Capitellacin	*C. teleta*	Gram+	***B. subtilis***	16 μM	[191]
			M. luteus	4 μM	
			S. aureus	8 μM	
		Gram-	***A. baumannii***	0.25 μM	
			E. cloacae	4 μM	
			E. coli	0.5-1 μM	
			K. pneumoniae	4 μM	
			P. aeruginosa	2 μM	
HfBRI-25	*H. filiformis*	Gram+	*B. licheniformis*	1 μM	[198]
			B. mycoides	0.5 μM	
			B. subtilis	1 μM	
			M. phlei	2 μM	
			M. smegmatis	1 μM	
			S. aureus	2 μM	
		Gram-	***A. baumannii***	0.125 μM	
			E. cloacae	1 μM	
			E. coli	0.25-0.5 μM	
			K. pneumoniae	2 μM	
			P. aeruginosa	2 μM	
			V. harveyi	1 μM	
HfBRI-28	*H. filiformis*	Gram+	*B. licheniformis*	8 μM	[198]
			B. mycoides	0.5 μM	
			B. subtilis	1 μM	
			M. phlei	2 μM	
			M. smegmatis	1 μM	
			S. aureus	8 μM	
		Gram-	***A. baumannii***	2 μM	
			E. cloacae	0.5 μM	
			E. coli	2 μM	
			K. pneumoniae	16 μM	
			P. aeruginosa	32 μM	
			V. harveyi	0.06 μM	
N1 (NZ17074)	*A. marina*	Gram+	***B. subtilis***	0.25 μg mL^{-1}	[199]
			L. ivanovii	128 μg mL^{-1}	
			S. aureus	0.5-16 μg mL^{-1}	
			S. suis	16 μg mL^{-1}	
		Gram-	***E. coli***	0.25-1 μg mL^{-1}	
			P. aeruginosa	2-4 μg mL^{-1}	
			S. choleraesuis	0.5 μg mL^{-1}	
			S. enteritidis	0.25 μg mL^{-1}	
			S. pullorum	0.25 μg mL^{-1}	
			S. typhimurium	0.5 μg mL^{-1}	
		Fungi	***C. albicans***	16 μg mL^{-1}	
N2	*A. marina*	Gram+	***B. subtilis***	0.5 μg mL^{-1}	[199]
			L. ivanovii	4 μg mL^{-1}	
			S. aureus	0.25-16 μg mL^{-1}	
			S. suis	16 μg mL^{-1}	
		Gram-	***E. coli***	0.25-0.5 μg mL^{-1}	
			P. aeruginosa	2 μg mL^{-1}	
			S. choleraesuis	0.5 μg mL^{-1}	
			S. enteritidis	0.125 μg mL^{-1}	
			S. pullorum	0.25-0.5 μg mL^{-1}	
			S. typhimurium	1 μg mL^{-1}	
		Fungi	***C. albicans***	32 μg mL^{-1}	
N3	*A. marina*	Gram+	***B. subtilis***	2 μg mL^{-1}	[199]
			L. ivanovii	8 μg mL^{-1}	
			S. aureus	0.25 μg mL^{-1}	
			S. suis	16 μg mL^{-1}	
		Gram-	***E. coli***	1 μg mL^{-1}	
			P. aeruginosa	16 μg mL^{-1}	
			S. choleraesuis	4 μg mL^{-1}	
			S. enteritidis	0.5 μg mL^{-1}	
			S. pullorum	1-2 μg mL^{-1}	
			S. typhimurium	4 μg mL^{-1}	
N4	*A. marina*	Gram+	***B. subtilis***	1 μg mL^{-1}	[199]
			S. aureus	1-8 μg mL^{-1}	
		Gram-	***E. coli***	1 μg mL^{-1}	
			P. aeruginosa	4 μg mL^{-1}	
			S. enteritidis	0.25 μg mL^{-1}	
			S. pullorum	1-2 μg mL^{-1}	
			S. typhimurium	2 μg mL^{-1}	
N5	*A. marina*	Gram+	***B. subtilis***	64 μg mL^{-1}	[199]
			L. ivanovii	128 μg mL^{-1}	
			S. aureus	2 μg mL^{-1}	

			Gram-	***E. coli***	64-128 μg mL^{-1}		
				S. enteritidis	4 μg mL^{-1}		
				S. typhimurium	128 μg mL^{-1}		
	N6	*A. marina*	Gram+	***B. subtilis***	0.5 μg mL^{-1}		[199]
				S. aureus	0.25-16 μg mL^{-1}		
				S. suis	16 μg mL^{-1}		
			Gram-	***E. coli***	0.5-1 μg mL^{-1}		
				P. aeruginosa	4-8 μg mL^{-1}		
				S. choleraesuis	2 μg mL^{-1}		
				S. enteritidis	0.25 μg mL^{-1}		
				S. pullorum	0.5 μg mL^{-1}		
				S. typhimurium	2 μg mL^{-1}		
			Fungi	***C. albicans***	64 μg mL^{-1}		
	N7	*A. marina*	Gram+	***B. subtilis***	2 μg mL^{-1}		[199]
				L. ivanovii	2 μg mL^{-1}		
				S. aureus	0.5 μg mL^{-1}		
				S. suis	8 μg mL^{-1}		
			Gram-	***E. coli***	2 μg mL^{-1}		
				S. choleraesuis	16 μg mL^{-1}		
				S. enteritidis	0.5 μg mL^{-1}		
				S. pullorum	4 μg mL^{-1}		
				S. typhimurium	8 μg mL^{-1}		
	N8	*A. marina*	Gram+	***B. subtilis***	4 μg mL^{-1}		[199]
				S. aureus	2 μg mL^{-1}		
			Gram-	***E. coli***	16-32 μg mL^{-1}		
				S. enteritidis	1 μg mL^{-1}		
				S. pullorum	32 μg mL^{-1}		
	Nicomicin-1	*N. minor*	Gram+	*B. licheniformis*	0.125 μM		[200]
				B. subtilis	0.062 μM		
				M. luteus	0.125 μM		
				Rhodococcus sp.	0.125 μM		
				S. aureus	2 μM		
			Gram-	***A. baumannii***	32 μM		
				E. coli	2-32 μM		
				P. aeruginosa	32 μM		
	NZ17125	*A. marina*	Gram-	***E. coli***	0.125 μg mL^{-1}		[190]
				K. pneumoniae	2 μg mL^{-1}		
	NZ17126	*A. marina*	Gram-	***E. coli***	0.125 μg mL^{-1}		[190]
				K. pneumoniae	1 μg mL^{-1}		
	NZ17143	*A. marina*	Gram-	***E. coli***	0.25 μg mL^{-1}		[190]
				K. pneumoniae	4 μg mL^{-1}		
	NZ17160	*A. marina*	Gram-	***E. coli***	0.125 μg mL^{-1}		[190]
				K. pneumoniae	2 μg mL^{-1}		
	NZ17211	*A. marina*	Gram-	***E. coli***	0.5 μg mL^{-1}		[190]
				K. pneumoniae	8 μg mL^{-1}		
	NZ17224	*A. marina*	Gram-	***E. coli***	0.06 μg mL^{-1}		[190]
				K. pneumoniae	0.5 μg mL^{-1}		
	NZ17228	*A. marina*	Gram-	***E. coli***	0.125 μg mL^{-1}		[190]
				K. pneumoniae	0.5 μg mL^{-1}		
	NZ17230	*A. marina*	Gram-	***E. coli***	0.25 μg mL^{-1}		[190]
				K. pneumoniae	1 μg mL^{-1}		
	Polaricin	*Amphitritides sp.*	Gram-	O. donghaensis	5 μM	20 μM	[193]
				Pseudomonas sp.	0.31 μM	5 μM	
				V. alginolyticus	0.625 μM	5 μM	
				V. fluvialis	40 μM	>40 μM	
	UuBRI-21	*U. unicinctus*	Gram+	*B. licheniformis*	2 μM		[191]
				B. subtilis	2 μM		
				S. aureus	2 μM		
			Gram-	***A. baumannii***	0.5 μM		
				E. cloacae	2 μM		
				E. coli	0.25-0.5 μM		
				K. pneumoniae	4 μM		
				P. aeruginosa	0.5-1 μM		
				P. mirabilis	1 μM		
Hedistin	Hedistin	*H. diversicolor*	Gram+	*D. nishinomiyaensis*	0.4-0.8 μM	1.6 μM	[201]
				M. luteus	0.4-0.8 μM	1.6 μM	
				S. aureus	3-6 μM	12.5 μM	
				S. epidermidis	3-6 μM	12.5 μM	
				S. haemolyticus	3-6 μM	12.5 μM	
				S. saprophyticus	3-6 μM	12.5 μM	
			Gram-	***V. alginolyticus***	0.8-1.6 μM		
Macin	PlTM	*P. linea*	Gram+	***S. iniae***	31.3 μM		[202]
			Gram-	**E. piscicida**	62.5 μM		
				E. coli	31.3 μM		
				V. alginolyticus	15.6 μM		
				**V. campbelii**	7.8 μM		
				V. harveyi	62.5 μM		
				**V. ordalii**	1.0 μM		
Perinerin	Perinerin	*P. aibuhitensis*	Gram+	***A. viridans***	6.2-17 μg mL^{-1}		[203]
				Arthrobacter sp.	50-100 μg mL^{-1}		
				B. megaterium	1.5-5 μg mL^{-1}		
				M. luteus	25-50 μg mL^{-1}		
				S. aureus	25-50 μg mL^{-1}		

Gram-	***E. coli***	12.5-25 µg mL^{-1}
	P. vulgaris	50-100 µg mL^{-1}
	P. aeruginosa	3.1-9.2 µg mL^{-1}
Fungi	C. tenuipes	12.5-25 µg mL^{-1}

Concentration ranges may reflect differences in strain susceptibility, peptide origin (native or synthetic), or variations in pH and salinity. Refer to the original source for more details. NV (No value) indicates that MIC or related metrics are not available; antimicrobial activity was confirmed through disk diffusion assay or gene expression analysis following an antimicrobial challenge.

Table S4. Antimicrobial activities of AMPs from aquatic invertebrate arthropods against pathogens relevant to aquaculture and human health (Pathogens specific to aquaculture species are shown in bold; those specific to humans are in italic; pathogens affecting both are in bold italic. Non-pathogenic bacteria are shown in regular text).

Protein Family	Compound	Species	Class	Microorganism	MIC	MLC	Reference
Class Chelicerata							
ALF	ALFSp	*S. paramamosain*	Gram+	***A. viridans***	1.56-3.125 µM		[204]
				B. megaterium	6.25-12.5 µM		
				M. luteus	0.195-0.93 µM	1.56 µM	
				S. aureus	25-50 µM		
				S. haemolyticus	1.56-3.12 µM		
			Gram-	***E. coli***	12.5-25 µM		
				V. harveyi	0.195-0.93 µM		
	ALFSp2	*S. paramamosain*	Gram+	***A. viridans***	3.13-6.25 µM		[205]
				M. luteus	3.13-6.25 µM		
			Gram-	***V. anguillarum***	12.5-25 µM		
				V. harveyi	12.5-25 µM		
	Anti-LPS factor	*L. polyphemus*	Gram-	S. minnesota	0.12-7.45 µg mL^{-1}		[206]
				S. typhimurium	1.3-10 µg mL-1		
	PtALF1	*P. trituberculatus*	Gram-	***P. aeruginosa***	1.01-2.01 µM		[207]
				V. alginolyticus	0.50-1.01 µM		
	PtALF3	*P. trituberculatus*	Gram+	***M. luteus***	9.26-18.52 µM		[207]
				S. aureus	37.04-74.09 µM		
			Gram-	***P. aeruginosa***	0.29-0.58 µM		
				V. alginolyticus	0.29-0.58 µM		
	PtALF4	*P. trituberculatus*	Gram-	***P. aeruginosa***	26.57-53.14 µM		[207]
				V. alginolyticus	1.66-3.32 µM		
	PtALF5	*P. trituberculatus*	Gram-	***E. tarda***	7.78-15.54 µM		[208]
				P. aeruginosa	15.54-31.08 µM		
				V. alginolyticus	3.89-7.78 µM		
	PtALF6	*P. trituberculatus*	Gram+	***M. luteus***	9.32-18.64 µM		[209]
				S. aureus	9.32-18.64 µM		
			Gram-	***P. aeruginosa***	9.32-18.64 µM		
				V. alginolyticus	1.17-2.33 µM		
	PtALF7	*P. trituberculatus*	Gram+	***M. luteus***	32.59-65.19 µM		[210]
				S. aureus	8.15-16.3 µM		
			Gram-	***E. tarda***	2.04-4.07 µM		
				P. aeruginosa	1.02-2.04 µM		
				V. alginolyticus	0.51-1.02 µM		
	Sp-ALF1	*S. paramamosain*	Gram+	***B. subtilis***	<1.6 µM	<3.1 µM	[211]
				C. glutamicum	<6.2 µM	<12.5 µM	
				M. luteus	<1.6 µM	<1.6 µM	
			Gram-	***E. coli***	<1.6 µM	<3.1 µM	
				P. aeruginosa	<6.2 µM	<6.2 µM	
				**P. fluorescens**	<1.6 µM	<3.1 µM	
				P. stutzeri	<1.6 µM	<3.1 µM	
				S. flexneri	<3.1 µM	<1.6 µM	
				V. alginolyticus	<1.6 µM	<3.1 µM	
				V. harveyi	<1.6 µM	<1.6 µM	
				V. parahaemolyticus	<1.6 µM	<3.1 µM	
	Sp-ALF2	*S. paramamosain*	Gram+	***B. subtilis***	<3.1 µM	<6.2 µM	[211]
				C. glutamicum	<3.1 µM	<12.5 µM	
				M. luteus	<1.6 µM	<3.1 µM	
			Gram-	***E. coli***	<1.6 µM	<12.5 µM	
				P. aeruginosa	<6.2 µM	<12.5 µM	
				**P. fluorescens**	<3.1 µM	<3.1 µM	
				P. stutzeri	<3.1 µM	<3.1 µM	
				S. flexneri	<3.1 µM	<3.1 µM	
				V. alginolyticus	<3.1 µM	<3.1 µM	
				V. harveyi	<3.1 µM	<6.2 µM	
				V. parahaemolyticus	<3.1 µM	<3.1 µM	
	SsALF	*S. serrata*	Gram+	*S. pyogenes*	100-200 µg mL^{-1}		[212]
			Gram-	***E. coli***	50–100 µg mL^{-1}		
				P. aeruginosa	25–50 µg mL^{-1}		
Defensin	Big defensin	*Limulus sp.*	Gram+	***S. aureus***	<2.5 µg mL^{-1}		[213]
			Gram-	***E. coli***	2.5 µg mL^{-1}		
				K. pneumoniae	1.3 µg mL^{-1}		
				S. minnesota	<0.6 µg mL^{-1}		
				S. typhimurium	<1.3 µg mL^{-1}		
			Fungi	***C. albicans***	10-20 µg mL^{-1}		
			Gram+	***S. aureus***	<2.5 µg mL^{-1*}		[214]
			Gram-	***E. coli***	2.5 µg mL^{-1*}		
			Fungi	***C. albicans***	20 µg mL^{-1*}		

				K. pastoris	42 μg mL^{-1*}		
Polyphemusin	Polyphemusin I	*L. polyphemus*	Gram+	***S. aureus***	6.3 μg mL^{-1}		[215]
			Gram-	***E. coli***	6.3 μg mL^{-1}		
				S. minnesota	3.1-6.3 μg mL^{-1}		
				S. typhimurium	3.1 μg mL^{-1}		
			Fungi	***C. albicans***	6.3 μg mL^{-1}		
			Gram+	***E. faecalis***	0.25 μg mL^{-1}		[216]
				S. aureus	0.5 μg mL^{-1}		
				S. epidermidis	0.25 μg mL^{-1}		
			Gram-	***E. coli***	0.125 μg mL^{-1}		
				P. aeruginosa	0.25 μg mL^{-1}		
				S. typhimurium	0.25 μg mL^{-1}		
			Fungi	***C. albicans***	1 μg mL^{-1}		
			Gram+	***B. subtilis***	0.25 μM		[217]
				M. luteus	0.5 μM		
				S. aureus	0.5-4 μM		
			Gram-	***E. coli***	0.062 μM		
				K. pneumoniae	0.5 μM		
				P. aeruginosa	0.5 μM		
	Polyphemusin II	*L. polyphemus*	Gram+	***S. aureus***	6.3-12.5 μg mL^{-1}		[215]
			Gram-	***E. coli***	12.5 μg mL^{-1}		
				S. minnesota	3.1-12.5 μg mL^{-1}		
				S. typhimurium	3.1-6.3 μg mL^{-1}		
			Fungi	***C. albicans***	6.3 μg mL^{-1}		
			Gram+	***B. subtilis***	0.5 μM		[217]
				M. luteus	1 μM		
				S. aureus	0.5-4 μM		
			Gram-	***E. coli***	0.031 μM		
				K. pneumoniae	0.5 μM		
				P. aeruginosa	0.5 μM		
	Polyphemusin III	*L. polyphemus*	Gram+	***B. subtilis***	0.5 μM		[217]
				M. luteus	0.5 μM		
				S. aureus	2-16 μM		
			Gram-	***E. coli***	0.25 μM		
				K. pneumoniae	2 μM		
				P. aeruginosa	0.5 μM		
	PV5	*L. polyphemus*	Gram+	***E. faecalis***	0.5 μg mL^{-1}		[216]
				S. aureus	1 μg mL^{-1}		
				S. epidermidis	0.25 μg mL^{-1}		
			Gram-	***E. coli***	0.25 μg mL^{-1}		
				P. aeruginosa	0.5-1 μg mL^{-1}		
				S. typhimurium	0.5 μg mL^{-1}		
			Fungi	***C. albicans***	2 μg mL^{-1}		
	PV7	*L. polyphemus*	Gram+	***E. faecalis***	0.25 μg mL^{-1}		[216]
				S. aureus	1 μg mL^{-1}		
				S. epidermidis	0.5 μg mL^{-1}		
			Gram-	***E. coli***	0.125-0.25 μg mL^{-1}		
				P. aeruginosa	0.5-1 μg mL^{-1}		
				S. typhimurium	0.5 μg mL^{-1}		
			Fungi	***C. albicans***	4 μg mL^{-1}		
	PV8	*L. polyphemus*	Gram+	***E. faecalis***	0.5 μg mL^{-1}		[216]
				S. aureus	1 μg mL^{-1}		
				S. epidermidis	0.5 μg mL^{-1}		
			Gram-	***E. coli***	0.25 μg mL^{-1}		
				P. aeruginosa	2 μg mL^{-1}		
				S. typhimurium	0.5 μg mL^{-1}		
			Fungi	***C. albicans***	16 μg mL^{-1}		
Tachycitin	Tachycitin	*T. tridentatus*	Gram+	***S. aureus***	56 μg mL^{-1*}		[218]
			Gram-	***E. coli***	2-33 μg mL^{-1*}		
				K. pneumoniae	32 μg mL^{-1*}		
				S. minnesota	41 μg mL^{-1*}		
				S. typhimurium	44 μg mL^{-1*}		
			Fungi	***C. albicans***	52 μg mL^{-1*}		
			Fungi	***C. albicans***	52 μg mL^{-1*}		[214]
				K. pastoris	41 μg mL^{-1*}		
Tachyplesin	Tachyplesin I	*T. tridentatus*	Gram+	***B. subtilis***	3.13 μg mL^{-1}		[215]
				S. aureus	3.1-6.3 μg mL^{-1}		
			Gram-	***E. coli***	1.6-3.1 μg mL^{-1}		
				P. aeruginosa	12.5 μg mL^{-1}		
				S. minnesota	1.6-3.1 μg mL^{-1}		
				S. typhimurium	0.8-3.1 μg mL^{-1}		
			Fungi	***C. albicans***	3.1 μg mL^{-1}		
				C. neoformans	1.56 μg mL^{-1}		
			Fungi	***C. albicans***	0.2 μg mL^{-1*}		[214]
				K. pastoris	0.1 μg mL^{-1*}		
			Gram+	***S. aureus***	3.1-6.3 μg mL-1		[219]
			Gram-	***E. coli***	6.3-12.5 μg mL^{-1}		
				S. minnesota	3.1-12.5 μg mL^{-1}		
				S. typhimurium	0.8-6.3 μg mL^{-1}		
			Fungi	*N. brasiliensis*		12.5 μM	[220]
				T. cruzi		12.5 μM	
			Gram+	***B. subtilis***	0.5 μM		[217]
				M. luteus	1 μM		

				S. aureus	0.5-8 μM		
			Gram-	***E. coli***	0.062 μM		
				K. pneumoniae	0.5 μM		
				P. aeruginosa	0.5 μM		
			Parasite	*L. donovani*	NV		[221]
	Tachyplesin II	*T. tridentatus*	Gram+	***S. aureus***	1.6-6.3 μg mL^{-1}		[215]
			Gram-	***E. coli***	3.1 μg mL^{-1}		
				S. minnesota	1.6-3.1 μg mL^{-1}		
				S. typhimurium	1.6-3.1 μg mL^{-1}		
			Fungi	***C. albicans***	3.1 μg mL^{-1}		
			Gram+	***B. subtilis***	0.5 μM		[217]
				M. luteus	1 μM		
				S. aureus	0.5-8 μM		
			Gram-	***E. coli***	0.062 μM		
				K. pneumoniae	1 μM		
				P. aeruginosa	0.5 μM		
	Tachyplesin III	*T. tridentatus*	Gram+	***B. subtilis***	1 μM		[217]
				M. luteus	2 μM		
				S. aureus	0.5-16 μM		
			Gram-	***E. coli***	0.062 μM		
				K. pneumoniae	0.5 μM		
				P. aeruginosa	0.5 μM		
Tachystatin	Tachystatin A1	*T. tridentatus*	Gram+	***S. aureus***	4.2 μg mL^{-1*}		[214]
			Gram-	***E. coli***	25 mL^{-1*}		
			Fungi	***C. albicans***	3 μg mL^{-1*}		
				K. pastoris	0.5 μg mL^{-1*}		
	Tachystatin A2	*T. tridentatus*	Gram+	***B. subtilis***	1.96 μg mL^{-1*}		[222]
				S. aureus	15.63 μg mL^{-1*}		
				S. epidermidis	1.96 μg mL^{-1*}		
			Gram-	***E. coli***	0.98 μg mL^{-1*}		
				S. typhimurium	1.96 μg mL^{-1*}		
	Tachystatin B	*T. tridentatus*	Gram+	***S. aureus***	7.4 μg mL^{-1*}		[214]
			Fungi	***C. albicans***	3 μg mL^{-1*}		
				K. pastoris	0.1 μg mL^{-1*}		
	Tachystatin C	*T. tridentatus*	Gram+	***S. aureus***	0.8 μg mL^{-1*}		[214]
			Gram-	***E. coli***	1.2 μg mL^{-1*}		
			Fungi	***C. albicans***	0.9 μg mL^{-1*}		
				K. pastoris	0.3 μg mL^{-1*}		
Tatritin	6His-tatritin	*T. tridentatus*	Gram+	***A. hydrophila***	31 μg mL^{-1}		[223]
				S. aureus	62 μg mL^{-1}		
				S. agalactiae	15.6 μg mL^{-1}		
			Gram-	***E. coli***	125 μg mL^{-1}		
				K. pneumoniae	62 μg mL^{-1}		
	Tatritin	*T. tridentatus*	Gram+	***A. hydrophila***	8-16 μM*		[224]
				S. aureus	2-4 μM*		
				S. agalactiae	8 μM*		
			Gram-	***E. coli***	2 μM*		
				K. pneumoniae	4-8 μM*		
Class Crustacea							
ALF	ALFFc-LBD7	*P. chinensis*	Gram+	***A. hydrophila***	2-4 μM		[225]
				M. luteus	1-2 μM		
				P. simplex	32-64 μM		
			Gram-	***E. coli***	32-64 μM		
	ALFPm3	*P. monodon*	Gram+	***A. viridans***	1.56-3.12 μM		[226]
				B. megaterium	0.19-0.39 μM		
				M. luteus	1.56-3.12 μM		
				S. aureus	50-100 μM		
			Gram-	***E. cloacae***	3.12-6.25 μM		
				E. coli	0.095-0.19 μM		
				K. pneumoniae	3.12-6.25 μM		
				P. carotovorum	1.56-3.12 μM		
				S. typhimurium	6.25-12.5 μM		
				V. alginolyticus	0.39-0.78 μM		
				V. anguillarum	0.78-1.56 μM		
				V. harveyi	0.78-1.56 μM		
				**V. penaeicida**	25-50 μM		
			Fungi	***F. oxysporum***	1.56–3.12 μM		
				B. cinerea	3.12–6.25 μM		
				P. crustosum	12.5–25 μM		
			Virus	**WSSV**			[227]
			Virus	**WSSV**			[228]
	ALFPm3-LBD	*P. monodon*	Gram+	***Bacillus sp.***	2 μM		[229]
				Exiguobacterium sp.	2 μM		
				S. aureus	64 μM		
			Gram-	***Acinetobacter sp.***	16 μM		
				Vibrio sp.	64 μM		
	ALFPm11-LBD	*P. monodon*	Gram+	***Bacillus sp.***	2 μM		[229]
				Exiguobacterium sp.	4 μM		
				S. aureus	16 μM		
			Gram-	***Acinetobacter sp.***	4 μM		
				E. coli	128 μM		
				Vibrio sp.	64 μM		
	ALFPm6#29–52	*P. monodon*	Gram+	***A. viridans***		25-50 μM	[230]

			B. megaterium		25-50 μM	
			M. luteus		25-50 μM	
		Gram-	***E. coli***		25-50 μM	
Cf-ALF2	*C. feriatus*	Gram+	***S. aureus***	5 μM	5 μM	[231]
		Gram-	***E. coli***	10 μM	20 μM	
csSALF$_{55–76}$	*P. monodon*	Parasite	*T. vaginalis*	100-200 μg mL^{-1}		[232]
EcLBD1	*P. carinicauda*	Gram+	***M. luteus***	4-8 μM		[233]
		Gram-	***V. anguillarum***	2-4 μM		
			V. harveyi	16-32 μM		
		Virus	**WSSV**			
EcLBD2	*P. carinicauda*	Gram-	***V. harveyi***	32-64 μM		[234]
EcLBD3	*P. carinicauda*	Gram+	***K. rhizophila***	32-64 μM		[234]
		Gram-	***P. damselae***	32-64 μM		
			V. harveyi	8-16 μM		
		Virus	**WSSV**			
EcLBD4	*P. carinicauda*	Gram+	***S. epidermidis***	32-64 μM		[234]
		Gram-	***V. alginolyticus***	16-32 μM		
		Virus	**WSSV**			
EcLBD5	*P. carinicauda*	Gram-	***V. alginolyticus***	16-32 μM		[234]
		Virus	**WSSV**			
EsALF-3	*E. sinensis*	Gram+	***B. subtilis***	135 μg mL^{-1}		[235]
		Gram-	***E. coli***	270 μg mL^{-1}		
			V. anguillarum	33.75 μg mL^{-1}		
FcALF1-LBD1	*P. chinensis*	Gram+	***M. luteus***	16-32 μM		[225]
		Gram-	***E. coli***	16-32 μM		
FcALF2-LBD2	*P. chinensis*	Gram+	***M. luteus***	1-4 μM		[236]
		Gram-	***V. anguillarum***	32-64 μM		
		Gram+	***A. hydrophila***	32-64 μM		[225]
			M. luteus	2-4 μM		
			P. simplex	1-2 μM		
FcALF4-LBD4	*P. chinensis*	Gram-	***E. coli***	8-16 μM		[225]
FcALF5-LBD5	*P. chinensis*	Gram+	P. simplex	16-32 μM		[225]
FcALF6-LBD6	*P. chinensis*	Gram-	***E. coli***	16-32 μM		[225]
FcALF8-LBD8	*P. chinensis*	Gram+	***M. luteus***	4-8 μM		[237]
			S. epidermidis	32-64 μM		
		Gram-	***E. coli***	32-64 μM		
			P. damselae	1-2 μM		
			V. alginolyticus	0.5-1 μM		
			V. harveyi	1-2 μM		
LitstyALF-B1	*P. stylirostris*	Gram+	***A. viridans***	2.5 μM		[238]
			B. megaterium	1.25-2.5 μM		
			M. luteus	1.25 μM		
			S. aureus	10 μM		
		Gram-	***E. coli***	2.5 μM		
			S. enterica	5 μM		
		Fungi	B. cinerea	10 μM		
			C. albicans	10 μM		
			F. oxysporum	2.5 μM		
			P. nodorum	2.5 μM		
			R. stolonifer	10 μM		
LitstyALF-D1	*P. stylirostris*	Gram+	*B. megaterium*	2.5 μM		[238]
		Gram-	***E. coli***	2.5 μM		
Litvan ALF-E	*P. vannamei*	Gram+	*C. stationis*	20-40 μM		[239]
			M. maritypicum	20-40 μM		
Litvan ALF-G	*P. vannamei*	Gram+	***B. cereus***	20-40 μM	20-40 μM	[239]
			B. subtilis	10-20 μM	>40 μM	
			C. stationis	5-10 μM	20-40 μM	
			M. maritypicum	5-10 μM	10-20 μM	
			M. luteus	10-20 μM	>40 μM	
			S. aureus	10-20 μM	>40 μM	
		Gram-	**V. nigripulchritudo**	20-40 μM	>40 μM	
lsSALF$_{55–76}$	*P. monodon*	Parasite	*T. vaginalis*	25-200 μg mL^{-1}		[232]
LvALF1	*P. vannamei*	Virus	**WSSV**			[240]
LvALF8-LBD	*P. vannamei*	Gram+	**K. gibsonii**	4-8 μM	4-8 μM	[241]
			S. aureus	4-8 μM	>64 μM	
			S. epidermidis	2-4 μM	32-64 μM	
		Gram-	***E. coli***	4-8 μM	>64 μM	
			P. damselae	2-4 μM	8-16 μM	
			V. alginolyticus	1-2 μM	4-8 μM	
			V. harveyi	1-2 μM	4-8 μM	
			**V. owensii**	1-2 μM	4-8 μM	
			V. parahaemolyticus	1-2 μM	4-8 μM	
MjALF-D2	*P. japonicus*	Virus	**WSSV**			[242]
Penmon ALF-B1	*P. monodon*	Gram+	***A. viridans***	1.25 μM		[238]
			B. megaterium	0.15-1.25 μM		
			M. luteus	10 μM		
		Gram-	***E. coli***	0.15 μM		
			S. enterica	2.5 μM		
			V. alginolyticus	0.6 μM		
			V. harveyi	10 μM		
		Fungi	B. cinerea	10 μM		
			C. albicans	10 μM		
			F. oxysporum	10 μM		

Arasin	Arasin 1	*H. araneus*	Gram+	C. glutamicum	0.8 μM	1.6-3.1 μM	[243]
				S. aureus	11.7 μM	>100 μM	
			Gram-	***E. coli***	7.5-12.5 μM	16.7-25 μM	
				V. anguillarum	4.1-6.3 μM	20.8-25 μM	
	Arasin-likeSp	*S. paramamosain*	Gram+	***A. viridans***	0.195-0.39 μM	12.5 μM	[244]
				B. subtilis	6.25-12.5 μM		
				M. luteus	0.39-0.78 μM		
				S. haemolyticus	6.25-12.5 μM		
			Gram-	***V. anguillarum***	3.125-6.25 μM	3.125 μM	
				V. harveyi	0.78-1.56 μM		
	Callinectin	*C. sapidus*	Gram-	***E. coli***		1.44 μM	[245]
	LvArasin-like	*P. vannamei*	Gram+	***A. hydrophila***	50 μM*		[246]
				B. subtilis	50 μM*		
				E. faecalis	50 μM*		
				M. luteus	12.5 μM*		
				S. aureus	25 μM*		
			Gram-	***E. coli***	25 μM*		
				P. aeruginosa	25 μM*		
				V. parahaemolyticus	6.25 μM*		
	Pc-arasin1	*P. clarkii*	Gram+	***A. hydrophila***	25 μM		[247]
				M. luteus	6.25 μM		
				S. aureus	12.5 μM		
			Gram-	***E. coli***	12.5 μM		
				V. anguillarum	6.25 μM		
	Pc-arasin2	*P. clarkii*	Gram+	***A. hydrophila***	25 μM		[247]
				B. subtilis	50 μM		
				M. luteus	25 μM		
				S. aureus	25 μM		
			Gram-	***E. coli***	12.5 μM		
				V. anguillarum	12.5 μM		
	Ss-arasin	*S. serrata*	Gram+	***S. aureus***	10 μM*		[248]
			Gram-	***E. coli***	20 μM*		
				P. aeruginosa	40 μM*		
Astacidin	Astacidin 1	*P. leniusculus*	Gram+	*B. megaterium*	1.9 μM		[249]
				B. subtilis	15 μM		
				M. luteus	12.8 μM		
			Gram-	***E. coli***	15 μM		
				S. flexneri	15 μM		
			Fungi	***C. albicans***	6.3 μM		[250]
				M. furfur	12.5 μM		
				T. beigelii	6.3 μM		
				T. rubrum	25 μM		
	Astacidin 2	*P. leniusculus*	Gram+	*B. megaterium*	1.03 μM		[251]
				B. subtilis	1.06 μM		
				M. luteus	5.44 μM		
				S. aureus	4.24 μM		
			Gram-	***E. coli***	2.12 μM		
				P. vulgaris	4.24 μM		
				P. aeruginosa	4.24 μM		
				S. flexneri	0.5 μM		
	PcAst-1a	*P. clarkii*	Gram+	***S. aureus***	32 μM	>32 μM	[252]
			Gram-	***E. coli***	32 μM	>32 μM	
	PcAst-1b/c	*P. clarkii*	Gram+	***S. aureus***	2 μM	4 μM	[252]
			Gram-	***A. baumannii***	2 μM	4 μM	
				E. coli	1-2 μM	8 μM	
	PcAst-2	*P. clarkii*	Gram+	***S. aureus***	32 μM	32 μM	[252]
			Gram-	***A. baumannii***	8 μM	8 μM	
				E. coli	32 μM	>32 μM	
Crustin	Amk1	*P. monodon*	Gram+	***E. faecalis***	35.6-230 μM		[253]
				M. luteus	11.1-1.7 μM		
				S. aureus	6.6-26 μM		
			Gram-	***V. harveyi***	6.6-53 μM		
	CruFc	*P. chinensis*	Gram+	***A. hydrophila***	16 μM		[254]
				B. cereus	4 μM		
				B. megaterium	2 μM		
				B. subtilis	8 μM		
				M. luteus	4 μM		
				S. aureus	2 μM		
			Gram-	***E. coli***	32 μM		
				K. pneumoniae	16 μM		
				V. anguillarum	32 μM		
			Fungi	***A. niger***	32 μM		
				R. arrhizus	32 μM		
	CrusEs	*E. sinensis*	Gram+	*B. thuringiensis*	0.11		[255]
				B. subtilis	0.23		
				M. luteus	0.11		
	Crus-likePm	*P. monodon*	Gram+	***A. viridans***	0.312-0.625 μM		[256]
				B. megaterium	1.25-2.5 μM		
				M. luteus	2.5-5 μM		
				S. aureus	5-10 μM		
				S. haemolyticus	2.5-5 μM		
			Gram-	***E. coli***	2.5-5 μM		
				K. pneumoniae	10-20 μM		

				V. harveyi	2.5-5 µM	
	Crustin-3	*P. monodon*	Virus	**WSSV**		[257]
	CshFc	*P. chinensis*	Gram+	***B. cereus***	16 µM	[254]
				B. megaterium	16 µM	
				B. subtilis	32 µM	
				M. luteus	32 µM	
				S. aureus	16 µM	
	SpCrus2	*S. paramamosain*	Gram+	*B. megaterium*	0.35-0.7 µM	[258]
				B. subtilis	0.7-1.4 µM	
				S. aureus	0.7-1.4 µM	
			Gram-	***E. coli***	2.8-5.6 µM	
				V. alginolyticus	1.4-2.8 µM	
				V. harveyi	0.7-1.4 µM	
				V. parahaemolyticus	0.7-1.4 µM	
			Fungi	***C. albicans***	2.8-5.6 µM	
	SpCrus3	*S. paramamosain*	Gram+	*B. megaterium*	<6.25 µM	[259]
				B. subtilis	<12.5 µM	
				S. aureus	<6.25 µM	
			Gram-	***V. parahaemolyticus***	<25 µM	
			Fungi	***C. albicans***	<25 µM	
	SpCrus4	*S. paramamosain*	Gram+	*B. megaterium*	<0.8 µM	[259]
				B. subtilis	<0.8 µM	
				S. aureus	<1.6 µM	
			Gram-	***V. alginolyticus***	<12.5 µM	
				V. harveyi	<25 µM	
				V. parahaemolyticus	<12.5 µM	
			Fungi	***C. albicans***	<12.5 µM	
	SpCrus5	*S. paramamosain*	Gram+	*B. megaterium*	<0.4 µM	[260]
				B. subtilis	<0.8 µM	
				S. aureus	<0.4 µM	
			Gram-	***E. coli***	<25 µM	
				V. harveyi	<25 µM	
				V. parahaemolyticus	<12.5 µM	
			Fungi	***C. albicans***	<1.6 µM	
	SpCrus6	*S. paramamosain*	Gram+	*B. megaterium*	<0.8 µM	[261]
				B. subtilis	<3.12 µM	
				S. aureus	<1.6 µM	
			Fungi	***C. albicans***	<6.25 µM	
	SWDPm2	*P. monodon*	Gram+	***A. viridans***	3.125-6.25 µM	[262]
				B. megaterium	12.5-25 µM	
				M. luteus	1.56-3.125 µM	
				S. aureus	25-50 µM	
Defensin	LvDBD	*P. vannamei*	Gram+	***A. hydrophila***	50 µM	[263]
				B. subtilis	25 µM	
				E. faecalis	25 µM	
				M. luteus	25 µM	
				S. aureus	25 µM	
			Gram-	***E. coli***	25 µM	
				P. aeruginosa	25 µM	
				V. parahaemolyticus	12.5 µM	
	Panusin	*P. argus*	Gram+	***B. subtilis***	2.3 µM*	[264]
				S. aureus	0.7 µM*	
			Gram-	***E. coli***	0.9 µM*	
				K. pneumoniae	1 µM*	
Glycine-rich	GRPSp	*S. paramamosain*	Gram+	***A. viridans***	6.25-12.5 µM	[244]
				M. luteus	12.5-25 µM	
	Spgly-AMP$_{26-62}$	*S. paramamosain*	Gram+	***B. subtilis***	NV	[265]
				S. aureus	NV	
			Gram-	***E. coli***	NV	
				V. parahaemolyticus	NV	
Hyastatin	Hyastatin	*H. araneus*	Gram+	C. glutamicum	0.4 µM	[266]
			Gram-	***E. coli***	12.5 µM µM	
			Fungi	***C. albicans***	6.3-12.5 µM	
				S. cerevisiae	12.5 µM	
	SpHyastatin	*S. paramamosain*	Gram+	***A. hydrophila***	1.25-2.5 µM	[267]
				C. glutamicum	1.25-2.5 µM	
				M. luteus	1.25-2.5 µM	
				S. aureus	0.63-1.25 µM	
			Gram-	**P fluorescens**	1.25-2.5 µM	
				P. stutzeri	0.63-1.25 µM	
Paralithocin	Paralithocin 1	*P. camtschaticus*	Gram+	C. divergens	200 µM	[268]
				C. funditum	200 µM	
				**C. maltaromaticum**	200 µM	
				C. mobile	100 µM	
				C. glutamicum	50 µM	
			Gram-	**A. salmonicida**	200 µM	
	Paralithocin 2	*P. camtschaticus*	Gram+	C. divergens	50 µM	[268]
				C. funditum	25 µM	
				**C. maltaromaticum**	50 µM	
				C. mobile	50 µM	
				C. glutamicum	12.5 µM	
	Paralithocin 3	*P. camtschaticus*	Gram+	C. divergens	25 µM	[268]
				C. funditum	12.5 µM	

				**C. maltaromaticum**	25 μM	
				C. mobile	12.5 μM	
				C. glutamicum	12.5 μM	
			Gram-	**A. salmonicida**	100 μM	
				V. anguillarum	50 μM	
				**Y. ruckeri**	100 μM	
Penaeidin	FenchiPEN5-1	*P. chinensis*	Gram+	***B. cereus***	1.56-12.5 μM	[269]
				B. megaterium	6.25-25 μM	
				B. subtilis	6.25-25 μM	
				B. thuringiensis	1.56-6.3 μM	
				M. luteus	0.78-6.25 μM	
				S. aureus	6.25-25 μM	
			Gram-	***E. coli***	25-50 μM	
				K. pneumoniae	3.13-12.5 μM	
			Fungi	***F. oxysporum***	3.13-6.25 μM	
				F. solani	3.13-6.25 μM	
				N. vagabunda	6.25-12.5 μM	
				V. dahliae	3.13-6.25 μM	
	FmPEN3	*P. merguiensis*	Gram+	***M. luteus***	6.25 μM	[270]
	FmPEN5	*P. merguiensis*	Gram+	***M. luteus***	12.5 μM	[270]
			Gram-	***V. parahaemolyticus***	50 μM	
	Litset Pen3-4	*P. setiferus*	Gram+	*B. megaterium*	12.5-25 μM	[271]
				B. subtilis	1.6-3.12 μM	
				M. luteus	0.4-0.8 μM	
			Gram-	***E. coli***	25-50 μM	
			Fungi	*C. neoformans*	0.8-6.25 μM	
				C. albicans	6.25-12.5 μM	
				C. lusitaniae	6.25-12.5 μM	
				Y. lipolytica	6.25-12.5 μM	
				N. glabratus	6.25-12.5 μM	
				P. inconspicua	3.12-6.25 μM	
				P. kudriavzevii	6.25-12.5 μM	
			Gram+	*B. megaterium*	12.5-25 μM	[272]
				B. subtilis	1.6-3.12 μM	
				M. luteus	0.4-0.8 μM	
			Gram-	***E. coli***	25-50 μM	
	Litset Pen4-1	*P. setiferus*	Gram+	***A. viridans***	1.9-2.92 μM	[273]
				M. luteus	1.9-2.92 μM	
			Gram-	***E. coli***	22-33 μM	
			Fungi	B. cinerea	4.38-6.57 μM	
				F. oxysporum	0.84-1.26 μM	
				P. crustosum	1.26-1.9 μM	
			Gram+	***B. subtilis***	12.5-25 μM	[271]
				M. luteus	1.9-2.2 μM	
			Gram-	***E. coli***	25-50 μM	
			Fungi	*C. neoformans*	0.8-12.5 μM	
				C. albicans	6.25-12.5 μM	
				C. lusitaniae	6.25-12.5 μM	
				Y. lipolytica	1.6-3.12 μM	
				N. glabratus	6.25-12.5 μM	
				P. inconspicua	6.25-12.5 μM	
				P. kudriavzevii	6.25-12.5 μM	
			Gram+	***B. subtilis***	12.5-25 μM	[272]
				M. luteus	1.9-2.25 μM	
			Gram-	***E. coli***	25-50 μM	
	LvBigPEN	*P. vannamei*	Virus	**WSSV**		[274]
			Gram+	***A. hydrophila***	25 μM	[275]
				B. subtilis	50 μM	
				E. faecalis	25 μM	
				M. luteus	25 μM	
				S. aureus	25 μM	
			Gram-	***E. coli***	25 μM	
				P. aeruginosa	25 μM	
				V. parahaemolyticus	12.5 μM	
	MjPen-II	*P. japonicus*	Gram+	*B. megaterium*	0.7 μM	[276]
				B. subtilis	0.7 μM	
				B. thuringiensis	0.35 μM	
				S. aureus	1.4 μM	
			Gram-	***E. coli***	0.7 μM	
				K. pneumoniae	0.35 μM	
				P. aeruginosa	0.35 μM	
				V. anguillarum	0.7 μM	
	Pen-2	*P. vannamei*	Gram+	***A. viridans***	1.25-2.5 μM	[277]
				B. megaterium	2.5-5 μM	
				M. luteus	2.5-5 μM	
			Gram-	***E. coli***	5-10 μM	
			Fungi	A. brassicicola	2.5-5 μM	
				B. cinerea	5-10 μM	
				N. haematococca	1.25-2.5 μM	
				F. oxysporum	5-10 μM	
				N. crassa	2.5-5 μM	
			Virus	**WSSV**		[274]
	Pen-3a	*P. vannamei*	Gram+	***M. luteus***	0.6–2.5 μM	[278]

			Gram+	***M. luteus***	0.6-1.25 μM		[277]
			Gram-	***E. coli***	2.5-5 μM		
			Fungi	***F. oxysporum***	5-10 μM		
			Gram+	***A. viridans***	0.8-1.6 μM		[273]
				B. megaterium	3.12-6.25 μM		
				M. luteus	0.8-1.6 μM		
			Gram-	***E. coli***	6.25-12.5 μM		
			Fungi	***F. oxysporum***	3.12-6.25 μM		
	Pen-3aQ	*P. vannamei*	Gram+	***A. viridans***	0.3-0.6 μM		[277]
				B. megaterium	2.5-5 μM		
				M. luteus	1.25-2.5 μM		
			Gram-	***E. coli***	5-10 μM		
			Fungi	A. brassicicola	2.5-5 μM		
				B. cinerea	5-10 μM		
				N. haematococca	1.25-2.5 μM		
				F. oxysporum	5-10 μM		
				N. crassa	1.25-2.5 μM		
Proline-rich	SpPR-AMP1	*S. paramamosain*	Gram+	***M. luteus***	0.78-1.56 μM	1.56 μM	[279]
			Gram-	***E. coli***	12.5 μM	50 μM	
				S. typhimurium	3.13-6.25 μM	50 μM	
				V. harveyi	0.195-0.39 μM	3.13 μM	
				V. parahaemolyticus	3.13-6.25 μM	6.25 μM	
Scygonadin	Scygonadin	*S. serrata*	Gram+	***M. luteus***	125 μg mL^{-1}		[280]
			Gram+	***A. hydrophila***	30-60 μM		[281]
				C. glutamicum	15-30 μM		
				M. luteus	7.5-15 μM		
				S. aureus	7.5-30 μM		
			Gram+	***A. hydrophila***	12.5-25 μM	25-50 μM	[282]
				B. subtilis	25-50 μM	>50 μM	
				C. glutamicum	12.5-25 μM	12.5-25 μM	
				M. luteus	6.25-12.5 μM	6.25-12.5 μM	
				S. aureus	6.25-12.5 μM	6.25-12.5 μM	
			Gram-	**P. fluorescens**	12.5-25 μM	25-50 μM	
				S. flexneri	25-50 μM	25-50 μM	
	SSAP	*S. serrata*	Gram+	***S. aureus***	50-100 μg mL^{-1}		[283]
				S. pyogenes	25-50 μg mL^{-1}		
			Gram-	***E. coli***	25-50 μg mL^{-1}		
				P. aeruginosa	12.5-25 μg mL^{-1}		
Scyreprocin	SCY2	*S. paramamosain*	Gram+	***B. subtilis***	12.5-25 μM		[284]
				C. glutamicum	25-50 μM		
				M. luteus	25-50 μM		
	Scyreprocin	*S. paramamosain*	Gram+	***B. subtilis***	1-2 μM	4-8 μM	[284]
				C. glutamicum	2-4 μM	2-4 μM	
				M. luteus	<0.5 μM	<0.5 μM	
				S. aureus	<0.5 μM	2-4 μM	
			Gram-	***E. coli***	2-4 μM	4-15 μM	
				**P. fluorescens**	<0.5 μM	1-2 μM	
				P. stutzeri	0.5-1 μM	1-2 μM	
				S. flexneri	<0.5 μM	>15 μM	
				V. alginolyticus	4-8 μM	16-32 μM	
				V. fluvialis	1-2 μM	2-4 μM	
				V. harveyi	2-4 μM	8-16 μM	
				V. parahaemolyticus	8-16 μM	>32 μM	
			Fungi	***A. niger***	4-8 μM	>32 μM	
				A. ochraceus	4-8 μM	>32 μM	
				A. fumigatus	4-8 μM	>32 μM	
				C. albicans	2-4 μM	16-32 μM	
				C. parapsilosis	16-32 μM	>32 μM	
				C. tropicalis	16-32 μM	>32 μM	
				C. neoformans	1-2 μM	8-16 μM	
				F. graminearum	8-16 μM	>32 μM	
				F. oxysporum	16-32 μM	>32 μM	
				F. solani	8-16 μM	>32 μM	
				K. pastoris	4-8 μM	>32 μM	
				N. crassa	16-32 μM	>32 μM	
				P. kudriavzevii	8-16 μM	>30 μM	
Sparanegtin	Sparanegtin	*S. paramamosain*	Gram+	***A. hydrophila***	12-24 μM	>48 μM	[285]
				B. subtilis	24-48 μM	>48 μM	
				S. aureus	12-24 μM	>48 μM	
				S. epidermidis	24-48 μM	>48 μM	
			Gram-	***E. coli***	24-48 μM	24-48 μM	
				P. aeruginosa	12-24 μM	24-48 μM	
				**P. fluorescens**	12-24 μM	>48 μM	
				S. flexneri	12-24 μM	>48 μM	
			Fungi	*C. neoformans*	24-48 μM	>48 μM	
				K. pastoris	24-48 μM	>48 μM	
Stylicin	Ls-Stylicin1	*P. stylirostris*	Gram-	**V. nigripulchritudo**	80 μM		[286]
				**V. penaeicida**	40 μM		
				**V. splendidus**	80 μM		
			Fungi	***F. oxysporum***	2.5 μM		
			Virus	**WSSV**			[287]

Concentration ranges may reflect differences in strain susceptibility, peptide origin (native or synthetic), or variations in pH and salinity. Refer to the original source for more details. NV (No value) indicates that MIC or related metrics are not available; antimicrobial activity was confirmed through disk diffusion assay or gene expression analysis following an antimicrobial challenge. *Values represent the IC50.

Table S5. Antimicrobial activities of AMPs from aquatic invertebrate chordates against pathogens relevant to aquaculture and human health (Pathogens specific to aquaculture species are shown in bold; those specific to humans are in italic; pathogens affecting both are in bold italic. Non-pathogenic bacteria are shown in regular text).

Protein Family	Compound	Species	Class	Microorganism	MIC	MLC	Reference
Subphylum Cephalochordata							
Bjamp1	IARR-Anal1	*B. japonicum*	Gram-	***A. baumannii***	16 µM		[288]
				K. pneumoniae	16 µM		
				P. aeruginosa	16 µM		
	IARR-Anal2	*B. japonicum*	Gram-	***A. baumannii***	4 µM		[288]
				K. pneumoniae	8 µM		
				P. aeruginosa	4 µM		
	IARR-Anal3	*B. japonicum*	Gram-	***A. baumannii***	4 µM		[288]
				K. pneumoniae	4 µM		
				P. aeruginosa	8 µM		
	IARR-Anal4	*B. japonicum*	Gram-	***A. baumannii***	4 µM		[288]
				K. pneumoniae	8 µM		
				P. aeruginosa	4 µM		
	IARR-Anal5	*B. japonicum*	Gram-	***A. baumannii***	4 µM		[288]
				K. pneumoniae	8 µM		
				P. aeruginosa	1 µM		
	IARR-Anal6	*B. japonicum*	Gram-	***A. baumannii***	4 µM		[288]
				K. pneumoniae	4 µM		
				P. aeruginosa	2 µM		
	IARR-Anal7	*B. japonicum*	Gram-	***A. baumannii***	32 µM		[288]
				K. pneumoniae	8 µM		
				P. aeruginosa	2 µM		
	IARR-Anal8	*B. japonicum*	Gram-	***A. baumannii***	16 µM		[288]
				K. pneumoniae	8 µM		
				P. aeruginosa	2 µM		
	IARR-Anal9	*B. japonicum*	Gram-	***A. baumannii***	8 µM		[288]
				K. pneumoniae	2 µM		
				P. aeruginosa	16 µM		
	IARR-Anal10	*B. japonicum*	Gram-	***A. baumannii***	4 µM		[288]
				K. pneumoniae	2 µM		
				P. aeruginosa	8 µM		
	IARR-mBjAMP1	*B. japonicum*	Gram-	***A. baumannii***	16 µM		[288]
				K. pneumoniae	8 µM		
				P. aeruginosa	8 µM		
	mBjAMP1	*B. japonicum*	Gram+	***M. luteus***	6.3 µg mL^{-1}		[287]
				S. aureus	6.3 µg mL^{-1}		
			Gram-	***E. coli***	6.3 µg mL^{-1}		
				V. anguillarum	12.5 µg mL^{-1}		
			Gram-	***A. baumannii***	16 µM		[288]
				K. pneumoniae	16 µM		
				P. aeruginosa	32 µM		
	mBjAMP-re	*B. japonicum*	Gram+	***B. subtilis***	12.5 µM		[289]
				S. aureus	12.5 µM		
				S. epidermidis	12.5 µM		
			Gram-	***E. coli***	12.5 µM		
				P. aeruginosa	12.5 µM		
				S. typhimurium	12.5 µM		
	mBjAMP-ox	*B. japonicum*	Gram+	***B. subtilis***	12.5 µM		[289]
				S. aureus	12.5 µM		
				S. epidermidis	12.5 µM		
			Gram-	***E. coli***	12.5 µM		
				P. aeruginosa	12.5 µM		
				S. typhimurium	12.5 µM		
	mBjAMP-ser	*B. japonicum*	Gram+	***B. subtilis***	12.5 µM		[289]
				S. aureus	12.5 µM		
				S. epidermidis	6.3 µM		
			Gram-	***E. coli***	6.3 µM		
				P. aeruginosa	6.3 µM		
				S. typhimurium	6.3 µM		
	mBjAMP-del	*B. japonicum*	Gram+	***B. subtilis***	6.3 µM		[289]
				S. aureus	12.5 µM		
				S. epidermidis	6.3 µM		
			Gram-	***E. coli***	6.3 µM		
				P. aeruginosa	6.3 µM		
				S. typhimurium	6.3 µM		
Defensin	BjBD	*B. japonicum*	Gram+	***S. aureus***	62.5 µg mL^{-1}		[290]
			Gram-	***A. hydrophila***	125 µg mL^{-1}		
				E. coli	125 µg mL^{-1}		
Subphylum Tunicata							
Clavanin	Clavanin-A	*S. clava*	Gram+	***E. faecalis***	0.8-1.4 µg mL^{-1}		[291]
				E. faecium	0.2-0.6 µg mL^{-1}		
				S. aureus	0.8-5.3 µg mL^{-1}		

			Gram-	***E. coli***	0.6-1.7 μg mL^{-1}	
				K. pneumoniae	1.5-4.1 μg mL^{-1}	
				L. monocytogenes	0.1-0.3 μg mL^{-1}	
				P. aeruginosa	0.5-1.3 μg mL^{-1}	
				S. typhimurium	0.9-1.9 μg mL^{-1}	
			Gram+	***S. aureus***	45 μM	[292]
			Gram-	***E. coli***	24 μM	
			Gram+	***B. subtilis***	6 μM	[293]
				E. faecalis	6 μM	
				S. aureus	12-48 μM	
				S. pyogenes	12 μM	
			Gram-	***E. coli***	12-48 μM	
				K. pneumoniae	6 μM	
				P. mirabilis	12 μM	
				P. aeruginosa	12 μM	
				S. enterica	6 μM	
			Gram+	***E. faecalis***	256 μg mL^{-1}	[294]
			Fungi	***C. albicans***	128 μg mL^{-1}	
			Gram+	***S. aureus***	10 μM	[295]
			Gram-	***A. hydrophila***	10 μM	
				E. coli	44 μM	
				S. pullorum	60 μM	
	Clavanin AK	*S. clava*	Gram+	***E. faecalis***	0.7-4.5 μg mL^{-1}	[291]
				E. faecium	0.3-0.6 μg mL^{-1}	
				S. aureus	2.4-6 μg mL^{-1}	
			Gram-	***E. coli***	0.6-1.7 μg mL^{-1}	
				K. pneumoniae	1.5-4.1 μg mL^{-1}	
				L. monocytogenes	0.4 μg mL^{-1}	
				P. aeruginosa	0.5-1.3 μg mL^{-1}	
				S. typhimurium	0.9-1.9 μg mL^{-1}	
	Clavanin-B	*S. clava*	Gram+	***E. faecalis***	256 μg mL^{-1}	[294]
			Gram-	***E. coli***	256 μg mL^{-1}	
			Fungi	***C. albicans***	64 μg mL^{-1}	
	Clavanin-C	*S. clava*	Gram+	***E. faecalis***	128 μg mL^{-1}	[294]
				S. aureus	128 μg mL^{-1}	
			Gram-	***E. coli***	128 μg mL^{-1}	
			Fungi	***C. albicans***	64 μg mL^{-1}	
	Clavanin-D	*S. clava*	Gram+	***E. faecalis***	128 μg mL^{-1}	[294]
				S. aureus	128 μg mL^{-1}	
			Gram-	***E. coli***	64 μg mL^{-1}	
			Fungi	***C. albicans***	16 μg mL^{-1}	
	Clavanin-E	*S. clava*	Gram+	***S. aureus***	4 μM	[295]
			Gram-	***A. hydrophila***	4 μM	
				E. coli	38 μM	
				S. pullorum	35 μM	
			Fungi	***C. albicans***	32 μg mL^{-1}	
	Clavanin MO	*S. clava*	Gram+	***B. subtilis***	1.5 μM	[293]
				E. faecalis	1.5 μM	
				S. aureus	6-24 μM	
				S. pyogenes	6 μM	
			Gram-	***E. coli***	6-12 μM	
				K. pneumoniae	3 μM	
				P. mirabilis	6 μM	
				P. aeruginosa	3 μM	
				S. enterica	3 μM	
	ClavF	*S. clava*	Gram+	***S. aureus***	4 μM	[295]
			Gram-	***A. hydrophila***	4 μM	
				E. coli	2 μM	
				S. pullorum	2 μM	
Clavaspirin	Clavaspirin	*S. clava*	Gram+	***S. aureus***	1-3 μg mL^{-1}	[296]
			Gram-	***E. coli***	1-3 μg mL^{-1}	
				L. monocytogenes	1-3 μg mL^{-1}	
				P. aeruginosa	1-3 μg mL^{-1}	
			Fungi	***C. albicans***	1-3 μg mL^{-1}	
			Gram+	***B. subtilis***	16-64 μM	[297]
				S. aureus	16-64 μM	
				S. epidermidis	4-32 μM	
			Gram-	***E. coli***	2-64 μM	
				L. monocytogenes	16-64 μM	
				P. vulgaris	8-64 μM	
				P. aeruginosa	32-64 μM	
				S. typhimurium	32-64 μM	
	CSP-1	*S. clava*	Gram+	***B. subtilis***	16-64 μM	[297]
				S. aureus	16-64 μM	
				S. epidermidis	4-16 μM	
			Gram-	***E. coli***	4-8 μM	
				L. monocytogenes	16-64 μM	
				P. vulgaris	8-64 μM	
				P. aeruginosa	32 μM	
				S. typhimurium	32-64 μM	
	CSP-2	*S. clava*	Gram+	***B. subtilis***	4 μM	[297]
				S. aureus	8-16 μM	
				S. epidermidis	4-32 μM	

			Gram-	***E. coli***	2-4 µM	
				L. monocytogenes	4-8 µM	
				P. vulgaris	4-8 µM	
				P. aeruginosa	16-32 µM	
				S. typhimurium	4-64 µM	
	CSP-3	*S. clava*	Gram+	***B. subtilis***	8-16 µM	[297]
				S. aureus	8 µM	
				S. epidermidis	4-8 µM	
			Gram-	***E. coli***	2-4 µM	
				L. monocytogenes	8-16 µM	
				P. vulgaris	4-8 µM	
				P. aeruginosa	32 µM	
				S. typhimurium	4-64 µM	
	CSP-4	*S. clava*	Gram+	***B. subtilis***	4 µM	[297]
				S. aureus	8 µM	
				S. epidermidis	4-8 µM	
			Gram-	***E. coli***	2-4 µM	
				L. monocytogenes	4 µM	
				P. vulgaris	4-8 µM	
				P. aeruginosa	32 µM	
				S. typhimurium	4-64 µM	
Dicynthaurin	Dicynthaurin	*H. aurantium*	Gram+	***S. aureus***	100 µg mL^{-1*}	[298]
			Gram-	***E. coli***	10-100 µg mL^{-1*}	
				L. monocytogenes	100 µg mL^{-1*}	
Halocidin	17Hc	*H. aurantium*	Gram+	***B. subtilis***	32-64 µg mL^{-1}	[299]
			Gram-	***E. coli***	16-32 µg mL^{-1}	
	18Hc	*H. aurantium*	Gram+	***B. subtilis***	8-16 µg mL^{-1}	[299]
			Gram-	***E. coli***	8-16 µg mL^{-1}	
	18HcKK	*H. aurantium*	Gram+	***B. subtilis***	8-16 µg mL^{-1}	[299]
			Gram-	***E. coli***	8-16 µg mL^{-1}	
	Di-16Hc	*H. aurantium*	Gram+	***B. subtilis***	32-64 µg mL^{-1}	[299]
			Gram-	***E. coli***	8-16 µg mL^{-1}	
	Di-17Hc	*H. aurantium*	Gram+	***B. subtilis***	2-4 µg mL^{-1}	[299]
			Gram-	***E. coli***	<1 µg mL^{-1}	
			Fungi	***C. albicans***	2-4 µg mL^{-1}	[299]
	Di-18Hc	*H. aurantium*	Gram+	***B. subtilis***	2-4 µg mL^{-1}	[299]
				Enterococcus sp.	2-4 µg mL^{-1}	
				S. aureus	2-8 µg mL^{-1}	
			Gram-	***E. coli***	<1 µg mL^{-1}	
				P. aeruginosa	2-16 µg mL^{-1}	
	Di-18HcKK	*H. aurantium*	Gram+	***B. subtilis***	2-4 µg mL^{-1}	[299]
				Enterococcus sp.	1-2 µg mL^{-1}	
				S. aureus	4-16 µg mL^{-1}	
			Gram-	***E. coli***	4-8 µg mL^{-1}	
				P. aeruginosa	2-8 µg mL^{-1}	
	Di-K19Hc	*H. aurantium*	Gram+	***B. subtilis***	2-4 µg mL^{-1}	[299]
				Enterococcus sp.	1-2 µg mL^{-1}	
				S. aureus	2-8 µg mL^{-1}	
			Gram-	***E. coli***	<1 µg mL^{-1}	
				P. aeruginosa	1-8 µg mL^{-1}	
			Fungi	***A. niger***	8-16 µg mL^{-1}	[300]
				A. terreus	8-16 µg mL^{-1}	
				C. albicans	1-4 µg mL^{-1}	
				C. neoformans	16-32 µg mL^{-1}	
				F. oxysporum	4-8 µg mL^{-1}	
				G. candidum	8-16 µg mL^{-1}	
				P. grisea	4-8 µg mL^{-1}	
				T. beigelii	2-4 µg mL^{-1}	
			Gram+	***Enterococcus sp.***	4 µg mL^{-1}	[301]
				S. aureus	4 µg mL^{-1}	
			Gram-	*L. monocytogenes*	4 µg mL^{-1}	
	Di-K19HcKK	*H. aurantium*	Gram+	***B. subtilis***	2-4 µg mL^{-1}	[299]
				Enterococcus sp.	1-2 µg mL^{-1}	
				S. aureus	2-16 µg mL^{-1}	
			Gram-	***E. coli***	<1 µg mL^{-1}	
				P. aeruginosa	2-8 µg mL^{-1}	
	Di-PH1	*H. aurantium*	Fungi	***C. albicans***	4-32 µg mL^{-1}	[302]
	Di-PH2	*H. aurantium*	Fungi	***C. albicans***	1-4 µg mL^{-1}	[302]
	Di-WP2	*H. aurantium*	Fungi	***C. albicans***	2-8 µg mL^{-1}	[302]
	Halocidin	*H. aurantium*	Gram+	***S. aureus***	NV	[303]
			Gram-	***P. aeruginosa***	NV	
			Gram+	***Enterococcus sp.***	64 µg mL^{-1}	[301]
				S. aureus	16 µg mL^{-1}	
			Gram-	*L. monocytogenes*	64 µg mL^{-1}	
	HHP1	*H. aurantium*	Fungi	***C. albicans***	2-8 µg mL^{-1}	[302]
	HHP2	*H. aurantium*	Fungi	***C. albicans***	8-32 µg mL^{-1}	[302]
	K19Hc	*H. aurantium*	Gram+	***B. subtilis***	4-8 µg mL^{-1}	[299]
			Gram-	***E. coli***	8-16 µg mL^{-1}	
			Fungi	***C. albicans***	8-32 µg mL^{-1}	[300]
				C. neoformans	16-32 µg mL^{-1}	
				F. oxysporum	8-16 µg mL^{-1}	
				G. candidum	16-32 µg mL^{-1}	
				P. grisea	4-8 µg mL^{-1}	

				T. beigelii	4-8 μg mL^{-1}		
	K19HcKK	*H. aurantium*	Gram+	***B. subtilis***	4-8 μg mL^{-1}		[299]
			Gram-	***E. coli***	8-16 μg mL^{-1}		
	Khal	*H. aurantium*	Gram+	***Enterococcus sp.***	8 μg mL^{-1}		[301]
				S. aureus	8 μg mL^{-1}		
			Gram-	*L. monocytogenes*	4 μg mL^{-1}		
	P113	*H. aurantium*	Fungi	***C. albicans***	4-16 μg mL^{-1}		
	PH1	*H. aurantium*	Fungi	***C. albicans***	8-32 μg mL^{-1}		[302]
	PH2	*H. aurantium*	Fungi	***C. albicans***	1-16 μg mL^{-1}		[302]
	WP1	*H. aurantium*	Fungi	***C. albicans***	4-32 μg mL^{-1}		
Halocyntin	Halocyntin	*H. papillosa*	Gram+	***A. viridans***	3.13-6.25 μM		[304]
				B. megaterium	0.39-1.56 μM		
				E. faecalis	6.25-12.5 μM		
				M. luteus	1.56-3.13 μM		
				S. aureus	1.56-12.5 μM		
			Gram-	***E. coli***	6.25-12.5 μM		
				K. aerogenes	6.25-50 μM		
				K. pneumoniae	1.56-25 μM		
				N. gonorrhoeae	25-50 μM		
				P. aeruginosa	25-50 μM		
				S. typhimurium	12.5-100 μM		
No Family	Ci-MAM-A24	*C. intestinalis*	Gram+	*B. megaterium*	0.02-0.2 μM	0.02-0.2 μM	[305]
				B. subtilis	0.02-0.2 μM	0.02-0.2 μM	
				**P. citreus**	0.1 μM	0.1 μM	
				S. aureus	0.4-1.6 μM	0.4-3.1 μM	
				S. epidermidis	0.2-0.4 μM	0.2-0.4 μM	
			Gram-	***E. coli***	0.05-0.4 μM	0.05-0.4 μM	
				K. pneumoniae	0.8-1.6 μM	0.8-1.6 μM	
				P. aeruginosa	0.1-0.8 μM	3.1 μM	
				S. marcescens	1.6-3.1 μM	0.1-0.8 μM	
				V. alginolyticus	1.6 μM	1.6-3.1 μM	
				V. anguillarum	3.1 μM	1.6 μM	
				Y. enterocolitica	0.4-0.8 μM	0.4-0.8 μM	
			Fungi	***C. albicans***	3.1-6.3 μM	3.1-6.3 μM	
	Ci-PAP-A22	*C. intestinalis*	Gram+	*B. megaterium*	0.1-0.2 μM	0.1-0.2 μM	[306]
				B. subtilis	0.8-3.1 μM	0.8-3.1 μM	
				S. aureus	6.3 μM	6.3-12.5 μM	
				S. epidermidis	6.3-25 μM	12.5 μM	
			Gram-	***E. coli***	0.8 μM	1.6 μM	
				K. pneumoniae	12.5 μM	12.5 μM	
				P. aeruginosa	6.3-12.5 μM	6.3-12.5 μM	
				Y. enterocolitica	1.6-3.1 μM	3.1-6.3 μM	
			Fungi	***C. albicans***	3.1-6.3 μM	3.1-12.5 μM	
	P-02	*C. intestinalis*	Gram+	*M. tetragenus*	NV		[307]
			Gram-	***E. coli***	NV		
				P. vulgaris			
	P-03	*C. intestinalis*	Gram+	*S. mutans*	NV		[307]
			Gram-	***E. coli***	NV		
	P-04	*C. intestinalis*	Gram+	*M. tetragenus*	NV		[307]
				S. aureus			
				S. mutans			
			Gram-	***E. coli***	NV		
				P. vulgaris			
				P. aeruginosa			
	P-05	*C. intestinalis*	Gram+	*M. tetragenus*	NV		[307]
			Gram-	***E. coli***	NV		
	P-10	*C. intestinalis*	Gram+	*M. tetragenus*	NV		[307]
				S. aureus			
				S. mutans			
			Gram-	***E. coli***	NV		
				P. vulgaris			
Papillosin	Papillosin	*H. papillosa*	Gram+	***A. viridans***	0.19-0.39 μM		[304]
				B. megaterium	0.5-3.13 μM		
				E. faecalis	0.78-1.56 μM		
				M. luteus	0.13-0.39 μM		
				S. aureus	0.05-0.10 μM		
			Gram-	***K. aerogenes***	0.78-1.56 μM		
				K. pneumoniae	0.39-0.78 μM		
				P. aeruginosa	3.13-6.25 μM		
				S. typhimurium	0.78-1.56 μM		
Styelin	Styelin A	*S. clava*	Gram+	***E. faecium***	1 μg mL^{-1}		[308]
				S. aureus	3 μg mL^{-1}		
			Gram-	***E. coli***	1-2 μg mL^{-1}		
				L. monocytogenes	1 μg mL^{-1}		
				P. aeruginosa	2 μg mL^{-1}		
				S. typhimurium	2 μg mL^{-1}		
			Fungi	***C. albicans***	36 μg mL^{-1}		
	Styelin B	*S. clava*	Gram+	***E. faecium***	1 μg mL^{-1}		[308]
				S. aureus	3 μg mL^{-1}		
			Gram-	***E. coli***	1-2 μg mL^{-1}		
				L. monocytogenes	1 μg mL^{-1}		
				P. aeruginosa	2 μg mL^{-1}		
				S. typhimurium	2 μg mL^{-1}		

			Fungi	***C. albicans***	36 μg mL^{-1}	
	Styelin D	*S. clava*	Gram+	***S. aureus***	5-30 μg mL^{-1}	[309]
			Gram-	***P. aeruginosa***	4.2-5.9 μg mL^{-1}	
Turgencin	StAMP-1	*Synoicum turgens*	Gram+	*B. megaterium*	250 μg mL^{-1}	[310]
				C. glutamicum	250 μg mL^{-1}	
	StAMP-2	*Synoicum turgens*	Gram+	*B. megaterium*	3.9 μg mL^{-1}	[310]
				B. subtilis	125 μg mL^{-1}	
				C. glutamicum	31.3 μg mL^{-1}	
				M. luteus	250 μg mL^{-1}	
			Fungi	*A. pullulans*	62.5 μg mL^{-1}	
				C. albicans	125 μg mL^{-1}	
				Rhodotorula sp.	62.5 μg mL^{-1}	
	StAMP-3	*Synoicum turgens*	Gram+	*B. megaterium*	3.9 μg mL^{-1}	[310]
				C. glutamicum	15.6 μg mL^{-1}	
				M. luteus	250 μg mL^{-1}	
			Fungi	*A. pullulans*	62.5 μg mL^{-1}	
				C. albicans	125 μg mL^{-1}	
				Rhodotorula sp.	62.5 μg mL^{-1}	
	StAMP-4	*Synoicum turgens*	Gram+	*B. megaterium*	3.9 μg mL^{-1}	[310]
				B. subtilis	125 μg mL^{-1}	
				C. glutamicum	3.9 μg mL^{-1}	
				M. luteus	125 μg mL^{-1}	
			Fungi	*A. pullulans*	62.5 μg mL^{-1}	
				C. albicans	62.5 μg mL^{-1}	
				Rhodotorula sp.	31.3 μg mL^{-1}	
	StAMP-5	*Synoicum turgens*	Gram+	*B. megaterium*	1 μg mL^{-1}	[310]
				B. subtilis	15.6 μg mL^{-1}	
				C. glutamicum	2 μg mL^{-1}	
				M. luteus	15.6 μg mL^{-1}	
			Gram-	***E. coli***	31.3 μg mL^{-1}	
				P. aeruginosa	250 μg mL^{-1}	
			Fungi	*A. pullulans*	31.3 μg mL^{-1}	
				C. albicans	31.3 μg mL^{-1}	
				Rhodotorula sp.	15.6 μg mL^{-1}	
	StAMP-6	*Synoicum turgens*	Gram+	*B. megaterium*	1 μg mL^{-1}	[310]
				B. subtilis	3.9 μg mL^{-1}	
				C. glutamicum	3.9 μg mL^{-1}	
				M. luteus	62.5 μg mL^{-1}	
				S. aureus	250 μg mL^{-1}	
			Gram-	***E. coli***	62.5 μg mL^{-1}	
			Fungi	*A. pullulans*	62.5 μg mL^{-1}	
				C. albicans	62.5 μg mL^{-1}	
				Rhodotorula sp.	31.3 μg mL^{-1}	
	StAMP-7	*Synoicum turgens*	Gram+	*B. megaterium*	1 μg mL^{-1}	[310]
				B. subtilis	3.9 μg mL^{-1}	
				C. glutamicum	2 μg mL^{-1}	
				M. luteus	31.3 μg mL^{-1}	
				S. aureus	125 μg mL^{-1}	
			Gram-	***E. coli***	31.3 μg mL^{-1}	
				P. aeruginosa	250 μg mL^{-1}	
			Fungi	*A. pullulans*	15.6 μg mL^{-1}	
				C. albicans	31.3 μg mL^{-1}	
				Rhodotorula sp.	15.6 μg mL^{-1}	
	StAMP-8	*Synoicum turgens*	Gram+	*B. megaterium*	3.9 μg mL^{-1}	[310]
				B. subtilis	7.8 μg mL^{-1}	
				C. glutamicum	7.8 μg mL^{-1}	
				M. luteus	15.6 μg mL^{-1}	
				S. aureus	125 μg mL^{-1}	
			Gram-	***E. coli***	62.5 μg mL^{-1}	
				P. aeruginosa	125 μg mL^{-1}	
			Fungi	*A. pullulans*	7.8 μg mL^{-1}	
				C. albicans	15.6 μg mL^{-1}	
				Rhodotorula sp.	15.6 μg mL^{-1}	
	StAMP-9	*Synoicum turgens*	Gram+	*B. megaterium*	1 μg mL^{-1}	[310]
				B. subtilis	3.9 μg mL^{-1}	
				C. glutamicum	2 μg mL^{-1}	
				M. luteus	3.9 μg mL^{-1}	
				S. aureus	7.8 μg mL^{-1}	
			Gram-	***E. coli***	7.8 μg mL^{-1}	
				P. aeruginosa	31.3 μg mL^{-1}	
			Fungi	*A. pullulans*	31.3 μg mL^{-1}	
				C. albicans	31.3 μg mL^{-1}	
				Rhodotorula sp.	15.6 μg mL^{-1}	
	StAMP-10	*Synoicum turgens*	Gram+	*B. megaterium*	3.9 μg mL^{-1}	[310]
				B. subtilis	7.8 μg mL^{-1}	
				C. glutamicum	7.8 μg mL^{-1}	
				M. luteus	15.6 μg mL^{-1}	
				S. aureus	62.5 μg mL^{-1}	
			Gram-	***E. coli***	15.6 μg mL^{-1}	
				P. aeruginosa	31.3 μg mL^{-1}	
			Fungi	*A. pullulans*	62.5 μg mL^{-1}	
				C. albicans	62.5 μg mL^{-1}	
				Rhodotorula sp.	15.6 μg mL^{-1}	

StAMP-11	*Synoicum turgens*	Gram+	*B. megaterium*	7.8 µg mL^{-1}	[310]
			C. glutamicum	31.3 µg mL^{-1}	
			M. luteus	62.5 µg mL^{-1}	
		Fungi	*A. pullulans*	250 µg mL^{-1}	
			C. albicans	125 µg mL^{-1}	
			Rhodotorula sp.	31.3 µg mL^{-1}	
Turgencin A	*Synoicum turgens*	Gram+	*B. megaterium*	0.5 µg mL^{-1}	[310]
			B. subtilis	1.5 µg mL^{-1}	
			C. glutamicum	1.5 µg mL^{-1}	
			M. luteus	8 µg mL^{-1}	
			S. aureus	23.3 µg mL^{-1}	
		Gram-	***E. coli***	3 µg mL^{-1}	
			P. aeruginosa	5.9 µg mL^{-1}	
		Fungi	*A. pullulans*	92.6 µg mL^{-1}	
			C. albicans	46.3 µg mL^{-1}	
			Rhodotorula sp.	23.2 µg mL^{-1}	
Turgencin A$_{Mox1}$	*Synoicum turgens*	Gram+	***B. subtilis***	0.4 µM	[311]
			C. glutamicum	0.4 µM	
			S. aureus	6.3 µM	
		Gram-	***E. coli***	0.8 µM	
			P. aeruginosa	1.6 µM	
Turgencin B	*Synoicum turgens*	Gram+	***B. subtilis***	1.6 µM	[311]
			C. glutamicum	1.6 µM	
		Gram-	***E. coli***	12.5 µM	
			P. aeruginosa	25 µM	
Turgencin B$_{Mox1}$	*Synoicum turgens*	Gram+	***B. subtilis***	1.6 µM	[311]
			C. glutamicum	3.1 µM	
		Gram-	***E. coli***	25 µM	
Turgencin B$_{Mox2}$	*Synoicum turgens*	Gram+	***B. subtilis***	25 µM	[311]
			C. glutamicum	25 µM	

Concentration ranges may reflect differences in strain susceptibility, peptide origin (native or synthetic), or variations in pH and salinity. Refer to the original source for more details. NV (No value) indicates that MIC or related metrics are not available; antimicrobial activity was confirmed through disk diffusion assay or gene expression analysis following an antimicrobial challenge. *Values represent the IC50.

Table S6. Antimicrobial activities of AMPs from cnidaria against pathogens relevant to aquaculture and human health (Pathogens specific to aquaculture species are shown in bold; those specific to humans are in italic; pathogens affecting both are in bold italic. Non-pathogenic bacteria are shown in regular text).

Protein Family	Compound	Species	Class	Microorganism	MIC	MLC	Reference
Subphylum Anthozoa							
AmAMP1	PT07	*A. millepora*	Gram+	*B. megaterium*	0.1 µM		[312]
				S. aureus	47 µM		
			Gram-	***Acinetobacter sp.***	1.5-2.9 µM		
				E. coli	11.7 µM		
	PT07_G	*A. millepora*	Gram+	*B. megaterium*	0.1 µM		[312]
				S. aureus	47 µM		
			Gram-	***Acinetobacter sp.***	2.9-5.9 µM		
				E. coli	11.7-23.5 µM		
				**V. coralliilyticus**	94 µM		
	PT07_R	*A. millepora*	Gram+	*B. megaterium*	0.1 µM		[312]
				S. aureus	47 µM		
			Gram-	***Acinetobacter sp.***	1.5-11.7 µM		
				**A. fischerii**	5.9-11.7 µM 23.5 µM		
				E. coli	94 µM		
				**V. coralliilyticus**			
Crassicorin	Crassicorin-I	*U. crassicornis*	Gram+	***B. subtilis***	11.49 µg mL^{-1}		[313]
			Gram-	***E. coli***	12.21 µg mL^{-1}		
				S. enterica	11.95 µg mL^{-1}		
Damicornin	Damicornin	*P. damicornis*	Gram+	*B. megaterium*	20 µM	>20 µM	[314]
				C. stationis	10 µM	10 µM	
				M. maritypicum	20 µM	>20 µM	
				M. luteus	1.25 µM	2.5 µM	
				S. aureus	5 µM	>20 µM	
Defensin	BDS-I	*A. sulcata*	Virus	MHV-A59	NV		[315]
			Gram-	***E. coli***	10 µM	20 µM	
Equinin	Equinin B	*A. equina*	Gram+	***M. luteus***	250 µg mL^{-1}		[316]
			Gram-	***E. coli***	250 µg mL^{-1}		
				V. alginolyticus	250 µg mL^{-1}		
Pd-AMP1	Pd-AMP1	*P. dilatata*	Gram+	***S. aureus***	NV		[317]
Subphylum Medusozoa							
Arminin	Arminin 1a	*H. vulgaris*	Gram+	*B. megaterium*	0.01 µM**	0.1 µM	[318]
				E. faecalis	0.8 µM**	1.6 µM	
				E. faecium	0.2 µM**	0.4-0.8 µM	
				S. aureus	0.05-0.2 µM**	0.4-0.8 µM	
			Gram-	***E. coli***	0.05-0.1 µM**	0.2-0.4 µM	
				K. pneumoniae	0.2 µM**	0.4-0.8 µM	
Aurelin	Aurelin	*A. aurita*	Gram+	*L. monocytogenes*	22.64 µg mL^{-1}		[319]
			Gram-	***E. coli***	7.66 µg mL^{-1}		
			Gram+	*B. megaterium*	10 µM		[320]
				M. luteus	40 µM		
Macin	Hydramacin-1	*H. vulgaris*	Gram+	***E. faecalis***	7.1 µM**	>14.3 µM	[321]

				S. haemolyticus	0.9 μM**	1.8 μM	
				S. pyogenes	7.1 μM**	>14.3 μM	
			Gram-	***A. baumannii***	1.8 μM**	7.1 μM	[188]
				C. freundii	0.5-0.9 μM**	0.9-7.1 μM	
				E. cloacae	0.5-0.9 μM**	0.9 μM	
				E. coli	0.2 μM**	0.9 μM	
				K. oxytoca	0.5 μM**	0.9 μM	
				K. pneumoniae	0.5 μM**	0.9 μM	
				P. mirabilis	0.9 μM**	14.3 μM	
				P. vulgaris	3.6 μM**	>14.3 μM	
				P. aeruginosa	14.3 μM**	>14.3 μM	
				S. typhimurium	0.5 μM**	0.9 μM	
				S. marcescens	1.8-14.3 μM**	>14.3 μM	
				Y. enterocolitica	0.2 μM**	0.9 μM	
			Gram+	*B. megaterium*	0.39 μg mL^{-1}		[188]
			Gram-	***E. coli***		3.13 μg mL^{-1}	
Periculin	Periculin-1	*H. vulgaris*	Gram+	*B. megaterium*	0.2-0.4 μM**		[322]

Concentration ranges may reflect differences in strain susceptibility, peptide origin (native or synthetic), or variations in pH and salinity. Refer to the original source for more details. NV (No value) indicates that MIC or related metrics are not available; antimicrobial activity was confirmed through disk diffusion assay or gene expression analysis following an antimicrobial challenge. *Values represent the IC50. **Values represent the LD90.

Table S7. Antimicrobial activities of AMPs from echinoderms against pathogens relevant to aquaculture and human health (Pathogens specific to aquaculture species are shown in bold; those specific to humans are in italic; pathogens affecting both are in bold italic. Non-pathogenic bacteria are shown in regular text).

Protein Family	Compound	Species	Class	Microorganism	MIC	Reference
Class Asteroidea						
PpCrAMP	PpCrAMP-1	*P. pectinifera*	Gram+	**B. subtilis**	33.8 μg mL^{-1}	[323]
				S. aureus	32.1 μg mL^{-1}	
			Gram-	***S. enterica***	8 μg mL^{-1}	
				S. flexneri	29.8 μg mL^{-1}	
	PpCrAMP-2	*P. pectinifera*	Gram+	**B. subtilis**	22.9 μg mL^{-1}	[323]
				M. luteus	153 μg mL^{-1}	
				S. aureus	15.6 μg mL^{-1}	
			Gram-	***A. hydrophila***	107.2 μg mL^{-1}	
				S. enterica	4.5 μg mL^{-1}	
				S. flexneri	12.2 μg mL^{-1}	
	PpCrAMP-3	*P. pectinifera*	Gram+	**B. subtilis**	38.3 μg mL^{-1}	[323]
				S. aureus	41.2 μg mL^{-1}	
			Gram-	***S. enterica***	8.1 μg mL^{-1}	
				S. flexneri	41.2 μg mL^{-1}	
	PpCrAMP$_{red}$	*P. pectinifera*	Gram+	**B. subtilis**	42.3 μg mL^{-1}	[323]
				M. luteus	82.9 μg mL^{-1}	
				S. aureus	91.2 μg mL^{-1}	
			Gram-	***S. enterica***	8.4 μg mL^{-1}	
				S. flexneri	24.2 μg mL^{-1}	
Class Echinoidea						
Centrocin	Centrocin 1	*S. droebachiensis*	Gram+	C. glutamicum	1.3 μM	[324]
				S. aureus	2.5 μM	
			Gram-	***E. coli***	1.3 μM	
				V. anguillarum	2.5 μM	
	Centrocin 1 (heavy chain)	*S. droebachiensis*	Gram+	C. glutamicum	0.4 μM	[324]
				S. aureus	3.1 μM	
			Gram-	***E. coli***	1.6 μM	
				V. anguillarum	0.8 μM	
			Fungi	B. cinerea	50 μM	
				C. albicans	6.3 μM	
				P. roqueforti	6.3 μM	
				S. cerevisiae	6.3 μM	
	Centrocin 2	*S. droebachiensis*	Gram+	C. glutamicum	1.3 μM	[324]
				S. aureus	5 μM	
			Gram-	***E. coli***	2.5 μM	
				V. anguillarum	2.5 μM	
	EeCentrocin 1	*E. esculentus*	Gram+	C. glutamicum	0.78 μM	[325]
				S. aureus	0.78 μM	
			Gram-	***E. coli***	0.1 μM	
				P. aeruginosa	0.78 μM	
	EeCentrocin 1 (heavy chain diBr)	*E. esculentus*	Gram+	**B. subtilis**	1.56 μM	[325]
				C. glutamicum	0.78 μM	
				S. aureus	3.13 μM	
				S. epidermidis	3.13-6.25 μM	
			Gram-	***E. coli***	1.56 μM	
				P. aeruginosa	1.56-3.13 μM	
			Fungi	*A. pullulans*	6.25 μM	
				C. albicans	50 μM	
				Rhodotorula sp.	12.5 μM	
	EeCentrocin 1 (heavy chain)	*E. esculentus*	Gram+	**B. subtilis**	1.56 μM	[325]
				C. glutamicum	0.39 μM	
				S. aureus	3.13 μM	
				S. epidermidis	3.13-6.25 μM	
			Gram-	***E. coli***	0.78-1.56 μM	

				P. aeruginosa	1.56-3.13 μM	
			Fungi	*A. pullulans*	12.5 μM	
				C. albicans	100 μM	
				Rhodotorula sp.	12.5 μM	
				S. cerevisiae	50 μM	
	EeCentrocin 2 (heavy chain)	*E. esculentus*	Gram+	***B. subtilis***	6.25 μM	[325]
				C. glutamicum	0.78 μM	
				S. aureus	6.25 μM	
				S. epidermidis	3.13 μM	
			Gram-	***E. coli***	1.56-6.25 μM	
				P. aeruginosa	0.78-6.25 μM	
			Fungi	*A. pullulans*	25 μM	
				C. albicans	50 μM	
				Cladosporium sp.	12.5 μM	
				Rhodotorula sp.	3.13 μM	
				S. cerevisiae	12.5 μM	
	HC(1-16)	*E. esculentus*	Fungi	*A. pullulans*	50 μM	[326]
				C. albicans	100 μM	
				Rhodotorula sp.	25 μM	
	HC(2-16)A7	*E. esculentus*	Fungi	***C. albicans***	100 μM	[326]
				Rhodotorula sp.	100 μM	
	HC(1-12)A8K12	*E. esculentus*	Fungi	*A. pullulans*	12.5 μM	[326]
				C. albicans	6.3 μM	
				Rhodotorula sp.	3.1 μM	
Paracentrin	Paracentrin 1	*P. lividus*	Gram+	***S. aureus***	6200-12500 μg mL^{-1}	[327]
				S. epidermidis	6200 μg mL^{-1}	
			Gram-	***P. aeruginosa***	12500 μg mL^{-1}	
Strongylocin	EeStrongylocin 2	*E. esculentus*	Gram+	C. glutamicum	1.56 μM	[325]
				S. aureus	3.13 μM	
			Gram-	***E. coli***	0.78 μM	
				P. aeruginosa	1.56 μM	
	SpStrongylocin 1	*S. purpuratus*	Gram+	C. glutamicum	7.5 μM	[328]
				S. aureus	15 μM	
			Gram-	***E. coli***	7.5 μM	
				V. anguillarum	15 μM	
	SpStrongylocin 2	*S. purpuratus*	Gram+	C. glutamicum	3.8 μM	[328]
				S. aureus	15 μM	
			Gram-	***E. coli***	7.5 μM	
				V. anguillarum	15 μM	
	Strongylocin 1	*S. droebachiensis*	Gram+	C. glutamicum	2.5 μM	[329]
				S. aureus	2.5 μM	
			Gram-	***E. coli***	5 μM	
				V. anguillarum	2.5 μM	
	Strongylocin 2	*S. droebachiensis*	Gram+	C. glutamicum	2.5 μM	[329]
				S. aureus	2.5 μM	
			Gram-	***E. coli***	5 μM	
				V. anguillarum	1.3 μM	
Class Holothuroidea						
Holothuroidin	H2d	*H. tubulosa*	Gram-	L. monocytogenes	1200-5000 μg mL^{-1}	[330]
	Holothuroidin 1	*H. tubulosa*	Gram+	***S. aureus***	12500 μg mL^{-1}	[331]
				S. epidermidis	12500 μg mL^{-1}	
			Gram-	***P. aeruginosa***	12500 μg mL^{-1}	
	Holothuroidin 2	*H. tubulosa*	Gram+	E. faecalis	12500 μg mL^{-1}	[331]
				S. aureus	12500 μg mL^{-1}	
				S. epidermidis	12500 μg mL^{-1}	
			Gram-	L. monocytogenes	5000 μg mL^{-1}	[330]
				P. aeruginosa	12500 μg mL^{-1}	

Concentration ranges may reflect differences in strain susceptibility, peptide origin (native or synthetic), or variations in pH and salinity. Refer to the original source for more details.

Table S8. Antimicrobial activity of AMPs from mollusks against pathogens relevant to aquaculture and human health (Pathogens specific to aquaculture species are shown in bold; those specific to humans are in italic; pathogens affecting both are in bold italic. Non-pathogenic bacteria are shown in regular text).

Protein Family	Compound	Species	Class	Microorganism	MIC	MLC	Reference
Class Bivalvia							
Defensin	A1	*C. virginica*	Gram+	***B. subtilis***	14 μg mL^{-1}		[332]
				S. mutans	68 μg mL^{-1}		
			Gram-	***A. hydrophila***	17 μg mL^{-1}		
				E. coli	42 μg mL^{-1}		
				P. aeruginosa	15 μg mL^{-1}		
				S. enterica	24 μg mL^{-1}		
				S. sonnei	40 μg mL^{-1}		
	A2	*C. virginica*	Gram+	***B. subtilis***	3.8 μg mL^{-1}		[332]
				S. epidermidis	24 μg mL^{-1}		
				S. mutans	9.9 μg mL^{-1}		
			Gram-	***A. hydrophila***	14 μg mL^{-1}		
				E. coli	2-13 μg mL^{-1}		
				P. aeruginosa	7.6 μg mL^{-1}		
				S. enterica	20 μg mL^{-1}		
				S. sonnei	18 μg mL^{-1}		

A3	*C. virginica*	Gram+	***B. subtilis***	0.4 µg mL^{-1}		[332]
			C. acnes	53.7 µg mL^{-1}		
			S. epidermidis	4.2 µg mL^{-1}		
			S. mutans	1.5 µg mL^{-1}		
		Gram-	***A. hydrophila***	3.3 µg mL^{-1}		
			E. coli	5-6.8 µg mL^{-1}		
			P. aeruginosa	3 µg mL^{-1}		
			S. enterica	6.5 µg mL^{-1}		
			S. sonnei	5.5 µg mL^{-1}		
		Fungi	***C. albicans***	54 µg mL^{-1}		
A4	*C. virginica*	Gram+	***B. subtilis***	2.5 µg mL^{-1}		[332]
			C. acnes	27.1 µg mL^{-1}		
			S. epidermidis	15.8 µg mL^{-1}		
			S. mutans	10.5 µg mL^{-1}		
		Gram-	***E. coli***	2.2-5.7 µg mL^{-1}		
			P. aeruginosa	2 µg mL^{-1}9.5		
		Fungi	***C. albicans***	17.2 µg mL^{-1}		
ApBD1	*A. purpuratus*	Gram+	***M. luteus***	NV		[333]
			S. aureus	NV		
		Gram-	***V. anguillarum***	NV		
			**V. splendidus**	NV		
AOD	*C. virginica*	Gram+	***L. lactis***	3 µg mL^{-1}		[334]
			S. aureus	2.4 µg mL^{-1}		
		Gram-	***E. coli***	7.6 µg mL^{-1}		
			V. parahaemolyticus	15 µg mL^{-1}		
Cg-Def	*C. gigas*	Fungi	***F. oxysporum***	9 µM		[335]
Cg-Defh1	*C. gigas*	Gram+	*B. megaterium*	0.06 µM		[336]
			C. stationis	4 µM		
			M. maritypicum	2 µM		
			M. luteus	0.03 µM		
			S. aureus	0.5-2 µM		
			S. haemolyticus	6 µM		
		Gram-	***E. coli***	40 µM		
Cg-Defh2	*C. gigas*	Gram+	*B. megaterium*	0.03 µM		[336]
			C. stationis	0.1 µM		
			M. maritypicum	1 µM		
			M. luteus	0.01 µM		
			S. aureus	0.12-0.25 µM		
			S. haemolyticus	2 µM		
		Gram-	***E. coli***	20 µM		
Cg-Defm	*C. gigas*	Gram+	*C. stationis*	0.1-0.2 µM		[335]
			M. luteus	0.005-0.01 µM		
			M. maritypicum	0.5-1 µM		
		Gram-	***E. coli***	35 µM		
		Gram+	*B. megaterium*	0.03 µM		[336]
			C. stationis	0.2 µM		
			M. maritypicum	1 µM		
			M. luteus	0.01 µM		
			S. aureus	0.25-2 µM		
			S. haemolyticus	2 µM		
		Gram-	***E. coli***	20 µM		
MCdef	*R. philippinarum*	Gram+	***S. aureus***	1.25-2.5 µM		[337]
			S. iniae	1.25-2.5 µM		
		Gram-	**A. logei**	10-20 µM		
			**A. salmonicida**	10-20 µM		
MGD-1	*M. galloprovincialis*	Gram+	***M. luteus***	0.08 µg mL^{-1}	0.16 µg mL^{-1}	[338]
		Gram-	***E. coli***	6.4 µg mL^{-1}	>6.4 µg mL^{-1}	
			V. alginolyticus	6.4 µg mL^{-1}	>6.4 µg mL^{-1}	
			**V. splendidus**	1.6 µg mL^{-1}	3.2 µg mL^{-1}	
			Vibrio sp.	6.4 µg mL^{-1}	>6.4 µg mL^{-1}	
MgDefdg	*M. gigas*	Gram+	***S. aureus***	25 µg mL^{-1*}		[339]
		Gram-	***A. hydrophila***	3 µg mL^{-1*}		
N-ter ApBD Q13R	*A. purpuratus*	Gram+	*M. maritypicum*	30 µM		[340]
			M. luteus	6.25 µM		
			S. aureus	15 µM		
		Gram-	***E. coli***	30 µM		
			**V. bivalvicida**	30 µM		
			**V. splendidus**	3.75-7.5 µM		
			**V. tasmaniensis**	3.75 µM		
Pv-Def	*P. viridis*	Gram-	***V. parahaemolyticus***	NV		[341]
RPD-1	*R. philippinarum*	Gram+	***B. subtilis***	76.8 µg mL^{-1}		[342]
			M. tetragenus	38.4 µg mL^{-1}		
			S. aureus	9.6 µg mL^{-1}		
		Gram-	***E. coli***	76.8 µg mL^{-1}		
			V. anguillarum	19.2 µg mL^{-1}		
			V. parahaemolyticus	19.2 µg mL^{-1}		
RpdefB	*R. philippinarum*	Gram+	***M. luteus***	1-2 µM		[343]
			S. aureus	4-8 µM		
		Gram-	***E. cloacae***	2-4 µM		
			E. coli	1-2 µM		
			K. aerogenes	4-8 µM		
			P. mirabilis	1-2 µM		
			V. anguillarum	1-2 µM		

				V. harveyi	1-2 μM	
				V. parahaemolyticus	1-2 μM	
				**V. splendidus**	0.5-1 μM	
	VpBD	*R. philippinarum*	Gram+	***Bacillus sp.***	13.13-26.26 μM	[344]
				S. aureus	1.64-3.28 μM	
			Gram-	***Enterobacter sp.***	13.13-26.26 μM	
				P. putida	1.64-3.28 μM	
				V. anguillarum	13.13-26.26 μM	
				**V. ichthyoenteri**	3.28-6.56 μM	
	VpDef	*R. philippinarum*	Gram+	***M. luteus***	6.25-12.5 μM	[345]
				S. aureus	25-50 μM	
			Gram-	***E. cloacae***	25-50 μM	
				K. aerogenes	12.5-25 μM	
				V. anguillarum	25-50 μM	
				P. mirabilis	50-100 μM	
				P. putida	25-50 μM	
				**V. splendidus**	50-100 μM	
Macin	Hc theromacin	*S. cumingii*	Gram+	B. bifidum	NV	[346]
				S. aureus	NV	
			Gram-	***A. hydrophila***	NV	
				E. coli	NV	
	Mytimacin 1	*M. coruscus*	Gram+	*B. megaterium*	2 μM	[347]
				B. subtilis	100 μM	
				M. luteus	100 μM	
				S. aureus	6 μM	
			Gram-	***E. coli***	50 μM	
				P. aeruginosa	50 μM	
				V. alginolyticus	50 μM	
				V. harveyi	100 μM	
				V. parahaemolyticus	100 μM	
	Mytimacin-4	*M. galloprovincialis*	Gram+	***B. subtilis***	0.315 μM	[348]
				M. luteus	1.24 μM	
			Gram-	***K. aerogenes***	1.24 μM	
				V. parahaemolyticus	0.62 μM	
			Gram+	***B. subtilis***	65 μg mL^{-1}	[349]
				L. monocytogenes	45 μg mL^{-1}	
			Gram-	***E. coli***	15 μg mL^{-1}	
				P. aeruginosa	25 μg mL^{-1}	
				S. typhimurium	20 μg mL^{-1}	
	Rp-hdmc	*R. philippinarum*	Gram-	**V. tapetis**	NV	[350]
	SwTheromacin	*S. woodiana*	Gram-	***A. hydrophila***	NV	[351]
	VpMacin	*R. philippinarum*	Gram+	***M. luteus***	4-8 μM	[352]
				S. aureus	8-16 μM	
			Gram-	***E. cloacae***	4-8 μM	
				E. coli	4-8 μM	
				P. mirabilis	2-4 μM	
				V. alginolyticus	1-2 μM	
				V. anguillarum	2-4 μM	
				V. harveyi	1-2 μM	
				V. parahaemolyticus	0.5-1 μM	
				**V. splendidus**	1-2 μM	
	VpMacin-1	*R. philippinarum*	Gram+	***M. luteus***	3-6 μM	[353]
				S. aureus	12-24 μM	
			Gram-	***E. cloacae***	3-6 μM	
				E. coli	3-6 μM	
				V. anguillarum	2-4 μM	
				V. harveyi	1-2 μM	
				V. parahaemolyticus	2-4 μM	
				**V. splendidus**	1-2 μM	
			Fungi	K. pastoris	2-8 μM	
	VpMacin-2	*R. philippinarum*	Gram+	***M. luteus***	1-2 μM	[353]
				S. aureus	3-6 μM	
			Gram-	***E. cloacae***	3-6 μM	
				E. coli	1-2 μM	
				V. anguillarum	0.5-1 μM	
				V. harveyi	0.5-1 μM	
				V. parahaemolyticus	1-2 μM	
				**V. splendidus**	0.5-1 μM	
			Fungi	K. pastoris	1-4 μM	
Molluscidin	ApMolluscidin	*A. pectinata*	Gram+	***B. subtilis***	2.1 μg mL^{-1}	[354]
			Gram-	***E. coli***	0.5 μg mL^{-1}	
	CgMolluscidin	*C. gigas*	Gram+	***B. subtilis***	1.3 μg mL^{-1}	[355]
				M. luteus	3 μg mL^{-1}	
				S. aureus	31.3 μg mL^{-1}	
			Gram-	***S. enterica***	0.4 μg mL^{-1}	
				V. parahaemolyticus	2.3 μg mL^{-1}	
Myticalin	Myticalin A5	*Mytilus spp.*	Gram+	***B. subtilis***	4 μM	[356]
				S. aureus	8-16 μM	
			Gram-	***A. baumannii***	4 μM	
				E. coli	2 μM	
				P. aeruginosa	4-8 μM	
		M. galloprovincialis	Gram-	***E. coli***	2-4 μM	[357]
	Myticalin A8	*Mytilus spp.*	Gram+	***B. subtilis***	4 μM	[356]

				S. aureus	16 µM		
			Gram-	***A. baumannii***	1 µM		
				E. coli	1 µM		
				P. aeruginosa	8 µM		
		M. galloprovincialis	Gram-	***E. coli***	2 µM		[357]
	Myticalin B1	*Mytilus spp.*	Gram+	***B. subtilis***	32 µM		[356]
			Gram-	***A. baumannii***	8 µM		
				E. coli	8-16 µM		
		M. galloprovincialis	Gram-	***E. coli***	16-32 µM		[357]
	Myticalin C6	*Mytilus spp.*	Gram+	***B. subtilis***	32 µM		[356]
				S. aureus	8 µM		
			Gram-	***A. baumannii***	2 µM		
				E. coli	4 µM		
				P. aeruginosa	32 µM		
		M. galloprovincialis	Gram-	***E. coli***	16-32 µM		[357]
	Myticalin C9	*Mytilus spp.*	Gram+	***B. subtilis***	4 µM		[356]
				S. aureus	2 µM		
			Gram-	***A. baumannii***	2 µM		
				E. coli	4 µM		
				P. aeruginosa	8 µM		
		M. galloprovincialis	Gram-	***E. coli***	4-8 µM		[357]
	Myticalin D2	*Mytilus spp.*	Gram+	***B. subtilis***	2 µM		[356]
				S. aureus	16-32 µM		
			Gram-	***A. baumannii***	2 µM		
				E. coli	4 µM		
				P. aeruginosa	4 µM		
		M. galloprovincialis	Gram-	***E. coli***	4-8 µM		[357]
Mytichitin	Mytichitin-CB	*M. coruscus*	Gram+	*B. megaterium*	3.13-6.25 µM		[358]
				B. subtilis	1.56-3.13 µM		
				M. luteus	1.56-3.13 µM		
				S. aureus	3.13-6.25 µM		
			Gram-	***P. aeruginosa***	50-100 µM		
				V. parahaemolyticus	50-100 µM		
			Fungi	***C. albicans***	6.25-12.5 µM		
	Mytichitin-CBD	*M. coruscus*	Gram+				[359]
			Gram-	***E. coli***	NV		
				P. aeruginosa	NV		
				V. harveyi	NV		
			Fungi	***C. albicans***	NV		
			Parasite	**Scuticociliatia**	NV		
Myticin	MytC[1-40]	*M. galloprovincialis*	Gram+	***E. faecalis***	4 µM	>64 µM	[360]
				S. aureus	64 µM	>64 µM	
			Gram-	***E. coli***	32 µM	>64 µM	
	MytC[9-28]	*M. galloprovincialis*	Gram+	***E. faecalis***	16 µM	>64 µM	[360]
	MytC[19-40]	*M. galloprovincialis*	Gram+	***E. faecalis***	8 µM	>64 µM	[360]
				S. aureus	32 µM	64 µM	
			Gram-	***E. coli***	8 µM	8 µM	
				P. aeruginosa	64 µM	>64 µM	
	MytC[29-40]	*M. galloprovincialis*	Gram+	***E. faecalis***	64 µM	>64 µM	[360]
	MytC[19-40]S	*M. galloprovincialis*	Gram+	***E. faecalis***	16 µM	>64 µM	[360]
				S. aureus	32 µM	>64 µM	
			Gram-	***E. coli***	16 µM	32 µM	
				P. aeruginosa	64 µM	>64 µM	
	MytC[19-40]Sox	*M. galloprovincialis*	Gram+	***E. faecalis***	16 µM	64 µM	[360]
				S. aureus	32 µM	>64 µM	
			Gram-	***E. coli***	8 µM	32 µM	
				P. aeruginosa	64 µM	>64 µM	
	Myticin A	*M. galloprovincialis*	Gram+	***A. viridans***	4.5-9 µM		[361]
				B. megaterium	2.25-4.5 µM		
				M. luteus	2.25-4.5 µM		
	Myticin B	*M. galloprovincialis*	Gram+	***A. viridans***	2-4 µM		[361]
				B. megaterium	1-2 µM		
				M. luteus	1-2 µM		
			Gram-	***E. coli***	120-210 µM		
				S. newport	120-210 µM		
			Fungi	***F. oxysporum***	5-10 µM		
	Myt-Cc	*M. galloprovincialis*	Virus	**OsHV-1**	NV		[362]
			Virus	*HSV-1*	NV		
				HSV-2	NV		
	Myt-Tat	*M. galloprovincialis*	Virus	*HSV-1*	3.03-4.91 µM**		[362]
				HSV-2	2.07-4.11 µM**		
Myticusin	Myticusin-1	*M. coruscus*	Gram+	*B. megaterium*	2.5-5 µM		[363]
				B. subtilis	1.2-2.5 µM		
				M. luteus	1.2-2.5 µM		
				S. aureus	2.5-5 µM		
			Gram-	***E. coli***	6.7-12.5 µM		
				V. harveyi	12.5-25 µM		
	Myticusin-beta	*M. coruscus*	Gram+	***B. cereus***	NV		[364]
				B. subtilis	NV		
				C. perfringens	NV		
				S. mutans	NV		
				S. aureus	NV		
				S. mutans	NV		

			Gram-	***E. coli***	NV		
				K. pneumoniae	NV		
				P. aeruginosa	NV		
				V. alginolyticus	NV		
	Myticusin-beta$_{84-93}$	*M. coruscus*	Gram+	***B. subtilis***	NV		[364]
				S. aureus	NV		
			Fungi	***C. albicans***	NV		
	Myticusin-beta$_{84-93}$ analog	*M. coruscus*	Gram+	***B. cereus***	NV		[364]
				B. subtilis	NV		
				E. faecalis	NV		
				L. garvieae	NV		
				S. mutans	NV		
				S. iniae	NV		
				S. parauberis	NV		
				S. vestibularis	NV		
				S. aureus	NV		
			Gram-	***E. tarda***	NV		
				E. coli	NV		
				E. cloacae	NV		
				K. pneumoniae	NV		
				P. mirabilis	NV		
				P. stuartii	NV		
				P. aeruginosa	NV		
				V. alginolyticus	NV		
				V. parahaemolyticus	NV		
			Fungi	***C. albicans***	NV		
Mytilin	Mytilin A	*M. edulis*	Gram+	***A. viridans***	0.6-1.2 µM		[365]
				B. megaterium	0.6-1.2 µM		
				E. faecalis	2.5-5 µM		
				M. luteus	0.6-1.2 µM		
				S. aureus	5-10 µM		
			Gram-	***E. coli***	2.5-10 µM		
				P. carrageenovora	2.5-5 µM		
	Mytilin B	*M. galloprovincialis*	Gram+	***E. faecalis***		0.17-0.35 µM	[366]
				L. monocytogenes		0.35-0.7 µM	
				M. luteus		0.17-0.35 µM	
				S. aureus			
			Gram-	***K. aerogenes***		1.4-2.8 µM	
				E. coli		0.7-1.4 µM	
				V. alginolyticus		1.4-2.8 µM	
				**V. splendidus**		0.17-0.35 µM	
				V. vulnificus		1.4-2.8 µM	
	Mytilin C	*M. galloprovincialis*	Gram+	***E. faecalis***		2.8-5.6 µM	[366]
				L. monocytogenes		0.7-1.4 µM	
				M. luteus		0.7-1.4 µM	
				S. aureus		0.7-1.4 µM	
			Gram-	***E. coli***		0.35-0.7 µM	
				K. aerogenes		1.4-2.8 µM	
				S. typhimurium		19-38 µM	
				V. alginolyticus		1.4-2.8 µM	
				V. harveyi		1.4-2.8 µM	
				**V. splendidus**		1.4-2.8 µM	
				V. vulnificus		1.4-2.8 µM	
	Mytilin D	*M. galloprovincialis*	Gram+	*L. monocytogenes*		0.7-1.4 µM	[366]
				M. luteus		0.17-0.35 µM	
				S. aureus		2.8-5.6 µM	
			Gram-	***E. coli***		0.35-0.7 µM	
				V. alginolyticus		0.75-1.5 µM	
			Fungi	***F. oxysporum***	0.7-1.4		
	Mytilin G1	*M. galloprovincialis*	Gram+	***E. faecalis***		1.4-2.8 µM	[366]
				L. monocytogenes		1.4-2.8 µM	
				M. luteus		0.7-1.4 µM	
Mytimycin	Mytimycin	*M. edulis*	Fungi	F. culmorum	NV		[365]
				N. crassa	NV		
Proline-rich	Ap	*A. purpuratus*	Fungi	***F. oxysporum***	2.15 µM		[367]
				**S. parasitica**	0.85 µM		
	Ap-S	*A. purpuratus*	Fungi	***F. oxysporum***	0.69 µM		[367]
				N. crassa	0.69 µM		
				**S. parasitica**	0.74 µM		
	Cg-lgPrp	*C. gigas*	Gram+	*B. megaterium*	20 µM		[336]
				M. luteus	80 µM		
	Cg-lgPrp P/Q	*C. gigas*	Gram+	***M. luteus***	80 µM		[336]
	Cg-Prp$_{20-36}$	*C. gigas*	Fungi	B. cinerea	100 µM		[368]
				F. oxysporum	200 µM		
	Cg-Prp$_{22-36}$	*C. gigas*	Gram+	***M. luteus***	100 µM		[368]
			Fungi	B. cinerea	100 µM		
				F. oxysporum	125 µM		
No family	URP20	*C. hongkongensis*	Gram+	***S. aureus***	2.84 µM*		[369]
			Gram-	***E. coli***	1.338 µM*		
				V. alginolyticus	4.21 µM*		
				V. parahaemolyticus	2.572 µM*		
			Fungi	***C. albicans***	3.283 µM*		
Class Cephalopoda							

OctoPartenopin	OctoPartenopin (P0)	*O. vulgaris*	Gram+	**S. aureus**	148-152 μM**		[370]
			Gram-	**P. aeruginosa**	197-203 μM**		
			Fungi	**C. albicans**	197-203 μM**		
	P1	*O. vulgaris*	Gram+	**S. aureus**	147-153 μM**		[370]
			Gram-	**P. aeruginosa**	95-105 μM**		
			Fungi	**C. albicans**	195-205 μM**		
	P2	*O. vulgaris*	Gram+	**S. aureus**	45-55 μM**		[370]
			Gram-	**P. aeruginosa**	48-52 μM**		
			Fungi	**C. albicans**	97-103 μM**		
	P3	*O. vulgaris*	Gram+	**S. aureus**	79-81 μM**		[370]
			Gram-	**P. aeruginosa**	47-53 μM**		
			Fungi	**C. albicans**	96-104 μM**		
	P4	*O. vulgaris*	Gram+	**S. aureus**	75-85 μM**		[370]
			Gram-	**P. aeruginosa**	48-5 μM**		
			Fungi	**C. albicans**	175-185 μM**		
Prohibitin-2	Octoprohibitin	*O. minor*	Gram-	**A. baumannii**	200 μg mL^{-1}	400 μg mL^{-1}	[371]
Protein 3	Octominin	*O. minor*	Fungi	**C. albicans**	50 μg mL^{-1}	200 μg mL^{-1}	[372]
	Octominin II	*O. minor*	Fungi	**C. albicans**	80 μg mL^{-1}	120 μg mL^{-1}	[373]
				C. auris	160 μg mL^{-1}	200 μg mL^{-1}	
				N. glabratus	55 μg mL^{-1}	100 μg mL^{-1}	
Protein 5	Octopromycin	*O. minor*	Gram-	**A. baumannii**	50 μg mL^{-1}	200 μg mL^{-1}	[374]
No family	AV19	*S. officinalis*	Gram-	**V. parahaemolyticus**	10-20 μM	20-50 μM	[375]
				**V. splendidus**	10-20 μM	10-20 μM	
	GK28	*S. officinalis*	Gram-	**E. coli**	≤5 μM	20-50 μM	[375]
				**V. aestuarianus**	10-20 μM	>50 μM	
				**V. anguillarum**	10-20 μM	20-50 μM	
				**V. alginolyticus**	≤5 μM	20-50 μM	
				**V. parahaemolyticus**	≤5 μM	10-20 μM	
				**V. splendidus**	≤5 μM	≤5 μM	
	GR21	*S. officinalis*	Gram+	*B. megaterium*	1-10 μM	1-10 μM	[376]
				E. faecalis	75-100 μM	100-150 μM	
				L. monocytogenes	25-50 μM	25-50 μM	
				**S. aureus**	5-10 μM	10-25 μM	
			Gram-	**A. salmonicida**	≤5 μM	≥50 μM	
				**E. coli**	1-10 μM	1-10 μM	
	KT19	*S. officinalis*	Gram+	*B. megaterium*	1-10 μM	1-10 μM	[376]
				E. faecalis	100-150 μM		
				**S. aureus**	10-25 μM	25-50 μM	
			Gram-	**A. salmonicida**	10-25 μM	10-25 μM	
				**E. coli**	1-10 μM	1-10 μM	
				**S. typhimurium**	75-100 μM	75-100 μM	
				**V. alginolyticus**	25-50 μM	25-50 μM	
	NF19	*S. officinalis*	Gram-	**V. splendidus**	5-10 μM	10-20 μM	[375]
	VA20	*S. officinalis*	Gram-	**E. coli**	25-50 μM	≥100 μM	[376]
Class Gastropoda							
Cecropin	Pom-2	*P. poeyana*	Gram+	*L. monocytogenes*	30 μg mL^{-1}		[377]
			Gram-	**P. aeruginosa**	30 μg mL^{-1}		
Closticin	Pom-1	*P. poeyana*	Gram+	*L. monocytogenes*	<5 μg mL^{-1}		[377]
			Gram-	**K. pneumoniae**	20 μg mL^{-1}		
				**P. aeruginosa**	<5 μg mL^{-1}		
			Virus	**ZIKV**			
Dolabellanin	Dolabellanin B2	*D. auricularia*	Gram+	**B. subtilis**		2.5 μg mL^{-1}	[378]
				**S. aureus**		20 μg mL^{-1}	
			Gram-	**E. coli**		20-40 μg mL^{-1}	
				H. influenzae		5 μg mL^{-1}	
				**V. vulnificus**		5 μg mL^{-1}	
		P. peronii	Gram+	**B. subtilis**	5 μg mL^{-1*}		[379]
				L. monocytogenes	10 μg mL^{-1*}		
				**S. aureus**			
			Gram-	**E. coli**	20 μg mL^{-1*}		
				**K. pneumoniae**	12.5 μg mL^{-1*}		
				**P. mirabilis**	15 μg mL^{-1*}		
				**P. aeruginosa**	25 μg mL^{-1*}		
				**S. marcescens**	10 μg mL^{-1*}		
Macin	HdMac	*H. discus hannai*	Gram-	**E. cloacae**	1-2 μM		[380]
				**E. coli**	1-2 μM		
				**P. mirabilis**	2-4 μM		
				**V. alginolyticus**	1-2 μM		
				**V. anguillarum**	0.5-1 μM		
				**V. parahaemolyticus**	2-4 μM		
	Mytimacin-6	*H. diversicolor*	Gram-	**V. harveyi**	NV		[381]
Molluscidin	HdMolluscidin	*H. discus*	Gram+	**B. subtilis**	0.8-15 μg mL^{-1}		[382]
				**S. aureus**	19 μg mL^{-1}		
			Gram-	**A. hydrophila**	3.3 μg mL^{-1}		
				**E. coli**	1.4-3.6 μg mL^{-1}		
				**P. aeruginosa**	4 μg mL^{-1}		
				**S. enterica**	1 μg mL^{-1}		
				**S. flexneri**	1.5 μg mL^{-1}		
				**V. parahaemolyticus**	2 μg mL^{-1}		
Proline-rich	Peptide 4	*R. venosa*	Gram-	**K. pneumoniae**	NV		[383]
	Peptide 7	*R. venosa*	Gram-	**K. pneumoniae**	NV		[383]
No family	Bb-AMP4	*F. bengalensis*	Gram+	**S. epidermidis**	8 μg mL^{-1}	16 μg mL^{-1}	[384]
	Cm-p1	*C. muricatus*	Fungi	**A. niger**	41 μM		[385]

				B. cinerea	20 μM	
				C. albicans	7-13 μM	
				C. parapsilosis	105 μM	
				C. neoformans	209 μM	
				F. oxysporum	20 μM	
				T. rubrum	3 μM	
			Fungi	***C. albicans***	32-64 μg mL^{-1}	[386]
				C. parapsilosis	256 μg mL^{-1}	
				C. neoformans	128 μg mL^{-1}	
				T. rubrum	32 μg mL^{-1}	
	Cm-p3	*C. muricatus*	Fungi	***C. albicans***	64-128 μg mL^{-1}	[386]
				T. rubrum	128 μg mL^{-1}	
	Cm-p4	*C. muricatus*	Fungi	***C. albicans***	32 μg mL^{-1}	[386]
				C. parapsilosis	256 μg mL^{-1}	
				T. rubrum	32 μg mL^{-1}	
	Cm-p5	*C. muricatus*	Fungi	***C. albicans***	10 μg mL^{-1}	[386]
				C. parapsilosis	32 μg mL^{-1}	
				C. neoformans	64 μg mL^{-1}	
				T. mentagrophytes	128 μg mL^{-1}	
				T. rubrum	10 μg mL^{-1}	

Concentration ranges may reflect differences in strain susceptibility, peptide origin (native or synthetic), or variations in pH and salinity. Refer to the original source for more details. NV (No value) indicates that MIC or related metrics are not available; antimicrobial activity was confirmed through disk diffusion assay or gene expression analysis following an antimicrobial challenge. *Values represent the IC50. **Values represent the IC80.

Table S9. Antimicrobial activity of AMPs from other invertebrates against microorganisms including pathogens relevant to aquaculture and human health (Pathogens specific to aquaculture species are shown in bold; those specific to humans are in italic; pathogens affecting both are in bold italic. Non-pathogenic bacteria are shown in regular text).

Protein Family	Compound	Species	Class	Microorganism	MIC	MLC	Reference
Phylum Nematoda							
Anisaxin	Anisaxin-1	*Anisakis spp.*	Gram+	***S. aureus***	16-32 μM	16-32 μM	[387]
			Gram-	***A. baumannii***	1 μM	1 μM	
				E. coli	1-2 μM	1-2 μM	
				K. pneumoniae	0.5-4 μM	1-4 μM	
				P. aeruginosa	4-8 μM	4-16 μM	
	Anisaxin-2P	*Anisakis spp.*	Gram+	***S. aureus***	32-64 μM	64 μM	[387]
			Gram-	***A. baumannii***	0.5-1 μM	0.5-1 μM	
				E. coli	0.5-2 μM	0.5-2 μM	
				K. pneumoniae	0.5-2 μM	0.5-2 μM	
				P. aeruginosa	4-16 μM	8-32 μM	
	Anisaxin-2S	*Anisakis spp.*	Gram+	***S. aureus***	4 μM	4 μM	[387]
			Gram-	***A. baumannii***	0.25-0.5 μM	0.5 μM	
				E. coli	0.5-1 μM	0.5-1 μM	
				K. pneumoniae	0.25-1 μM	0.5-1 μM	
				P. aeruginosa	1-8 μM	2-8 μM	
	Anisaxin-3	*Anisakis spp.*	Gram-	***A. baumannii***	0.25-0.5 μM	0.25-0.5 μM	[387]
				E. coli	0.25-1 μM	0.5-4 μM	
				K. pneumoniae	0.5-2 μM	4-32 μM	
				P. aeruginosa	4-32 μM		
	Anisaxin-4	*Anisakis spp.*	Gram+	***S. aureus***	2 μM	2 μM	[387]
			Gram-	***A. baumannii***	0.5-2 μM	0.5-2 μM	
				E. coli	0.5-2 μM	0.5-2 μM	
				K. pneumoniae	0.5-2 μM	0.5-2 μM	
				P. aeruginosa	2-4 μM	2-4 μM	
Phylum Placozoa							
Trichoplaxin	Trichoplaxin	*T. adhaerens*	Gram+	***E. faecalis***	12.5 μM		[388]
				L. ivanovii	3 μM		
				S. aureus	3 μM		
			Gram-	***A. baumannii***	3 μM		
				E. coli	3-6 μM		
				K. pneumoniae	6 μM		
				P. aeruginosa	6 μM		
			Fungi	***C. albicans***	25 μM		
				C. parapsilosis	50 μM		
	Trichoplaxin-2	*T. adhaerens*	Gram+	***S. aureus***	1-4 μM		[389]
			Gram-	***A. baumannii***	1-8 μM		
				E. coli	0.5-8 μM		
				K. pneumoniae	4-8 μM		
				P. aeruginosa	4-32 μM		
	Trichoplaxin-2A	*T. adhaerens*	Gram+	***S. aureus***	0.5-4 μM		[389]
			Gram-	***A. baumannii***	2-8 μM		
				E. coli	1-4 μM		
				K. pneumoniae	2-4 μM		
				P. aeruginosa	1-8 μM		
Phylum Platyhelminthes							
Dermaseptin	SmDLP	*S. mansoni*	Gram+	***M. luteus***	0.0746 μM	1.18 μM	[390]
			Fungi	***C. albicans***	0.0746 μM	0.0746 μM	
Phylum Porifera							
ASABF_SUBDO	Defensin	*S. domuncula*	Gram+	***B. subtilis***	4.2-5.4 μg mL^{-1}		[391]
				M. luteus	2.6-4.6 μg mL^{-1}		
				S. aureus	1.2-2.2 μg mL^{-1}		

Gram-	***E. coli***	11.4-23.4 μg mL^{-1}
	P. aeruginosa	6.9-17.9 μg mL^{-1}

Concentration ranges may reflect differences in strain susceptibility, peptide origin (native or synthetic), or variations in pH and salinity. Refer to the original source for more details. NV (No value) indicates that MIC or related metrics are not available; antimicrobial activity was confirmed through disk diffusion assay or gene expression analysis following an antimicrobial challenge. *Values represent the IC50. **Values represent the IC80.

Table S10. Activity of "non classical AMPs" derived from aquatic invertebrates against pathogens relevant to aquaculture and human health (Pathogens specific to aquaculture species are shown in bold; those specific to humans are in italic; pathogens affecting both are in bold italic. Non-pathogenic bacteria are shown in regular text).

Category	Compound	Species	Bacteria		Fungi/Parasite	Ref.
			Gram+	Gram-		
Phylum Arthropoda						
Hemerythrin	MsHemerycin	*M. sanguínea*				[392]
β-thymosin	CgTβ	*C. gigas*	***B. subtilis*** NV	***E. coli*** NV	***C. abicans*** NV	[393]
Histone	MrHis	*M. rosenbergii*				[394]
Lysozyme	Lysozyme C	*P. merguiensis*				[395]
	PmLyzc	*P. monodon*				[396]
	PmLyzi1	*P. monodon*				[396]
	PmLyzi2	*P. monodon*				[396]
Phylum Chordata						
RPS23	BjRPS23	*B. japonicum*	***M. luteus*** 3.6 μM<br>***S. aureus***3.6 μM	***A. hydrophila*** 4.8 μM<br>***E. coli*** 4.8 μM		[397]
	BjRPS23$_{67-84}$	*B. japonicum*	***M. luteus*** 7.2 μM<br>***S. aureus*** 7.2 μM	***A. hydrophila*** 8.4 μM<br>***E. coli*** 8.4 μM		[397]
Plicatamide	PL-101	*S. plicata*	***S. aureus*** 1-125 μg mL^{-1}	***E. coli*** 5-95 μg mL^{-1}<br>*L. monocytogenes* 3-40 μg mL^{-1}<br>***P. aeruginosa*** 10-40 μg mL^{-1}		[398]
	PL-102	*S. plicata*	***S. aureus***50 μg mL^{-1}	***E. coli***130 μg mL^{-1}<br>***P. aeruginosa***140 μg mL^{-1}		[398]
	PL-103	*S. plicata*	***S. aureus***60-140 μg mL^{-1}	***E. coli***230 μg mL^{-1}<br>***P. aeruginosa***250 μg mL^{-1}		[398]
	PL-104	*S. plicata*	***S. aureus***50-60 μg mL^{-1}	***E. coli***130 μg mL^{-1}<br>***P. aeruginosa***250 μg mL^{-1}		[398]
	Plicatamide	*S. plicata*	***S. aureus***1-40 μg mL^{-1}	***E. coli***3-10 μg mL^{-1}<br>*L. monocytogenes*3-11 μg mL^{-1}<br>***P. aeruginosa***3-5 μg mL^{-1}		[398]
Phylum Cnidaria						
Neuropeptide	Hym-357	*H. vulgaris*				[399]
	Hym-370	*H. vulgaris*				[399]
	NDA-1	*H. vulgaris*				[399]
	RFamide III	*H. vulgaris*				[399]
Toxin	A3	*E. japonica*				[400]
	B1	*E. japonica*				[400]
	B4	*E. japonica*				[400]
	Tau-AnmTx Ueq 12-1	*U. eques*				[401]
Phylum Mollusca						
Aplysianin	Aplysianin-A	*A. kurodai*	***B. subtilis*** 4 μg mL^{-1*}			[402]
	Aplysianin-P	*A. kurodai*	*S. aureus* 0.39 μg mL^{-1*}<br>*S. epidermidis* 0.23 μg mL^{-1*}	***E. coli*** 2.8 μg mL^{-1*}<br>***E. cloacae*** 1.1 μg mL^{-1*}<br>***K. pneumoniae*** 0.2 μg mL^{-1*}<br>***S. typhimurium*** 5.8 μg mL^{-1*}<br>***S. marcescens*** 0.21 μg mL^{-1*}		[403]
Dolabellanin	Dolabellanin A	*D. auricularia*	***A. hydrophila*** 0.26 μg mL^{-1*}<br>***S. aureus*** 0.025 μg mL^{-1*}<br>***S. epidermidis*** 0.018 μg mL^{-1*}<br>***Streptococcus sp.*** 0.063 μg mL^{-1*}	***E. coli*** 0.025-0.09 μg mL^{-1*}<br>***E. cloacae*** 0.98 μg mL^{-1*}<br>***K. pneumoniae*** 0.021 μg mL^{-1*}<br>***S. typhimurium*** 0.48 μg mL^{-1*}<br>***S. marcescens*** 0.048 μg mL^{-1*}	***C. albicans*** 0.5 μg mL^{-1}<br>*S. cerevisiae* 1 μg mL^{-1}<br>S. pombe 2 μg mL^{-1}	[404]
Escapin	Escapin	*A. californica*	***B. subtilis*** 2.5 μg mL^{-1}<br>***S. aureus*** 0.31 μg mL^{-1}<br>*S. pyogenes* 0.62 μg mL^{-1}	***E. coli*** 0.62 μg mL^{-1}<br>***P. aeruginosa*** 0.31 μg mL^{-1}<br>***S. typhimurium*** 0.62 μg mL^{-1}<br>***V. harveyi*** 0.25 μg mL^{-1}	***C. sphaerospermum*** 15-62 μg mL^{-1}<br>*P. kudriavzevii* 5 μg mL^{-1}<br>*S. cerevisiae* 5 μg mL^{-1}	[405]
HDH-LGBP	HDH-LGBP-A1	*H. discus hannai*	***B. cereus*** 1.9 μg mL^{-1}<br>***S. aureus*** 1.08 μg mL^{-1}<br>***S. iniae*** 0.57 μg mL^{-1}<br>*S. mutans* 0.008 μg mL^{-1}	***P. aeruginosa*** 2.12 μg mL^{-1}	***C. albicans*** 2.11 μg mL^{-1}	[406]
	HDH-LGBP-A2	*H. discus hannai*	***B. cereus*** 1.8 μg mL^{-1}<br>***S. aureus*** 1.37 μg mL^{-1}<br>***S. iniae*** 1.79 μg mL^{-1}<br>*S. mutans* 1.7 μg mL^{-1}	***P. aeruginosa*** 1.92 μg mL^{-1}	***C. albicans*** 2.16 μg mL^{-1}	[406]
Hemocyanin-derived	Haliotisin P1	*H. tuberculata*		P. carotovorum 3-4 μM		[407]
	Haliotisin P2	*H. tuberculata*		P. carotovorum 4-5 μM		[407]
	Haliotisin P3	*H. tuberculata*	***B. subtilis*** 0.3-1 μM			[407]
	Haliotisin P4	*H. tuberculata*		P. carotovorum 1.6-2.6 μM		[407]

	Haliotisin P5	*H. tuberculata*		P. carotovorum 1.2-1.5 μM		[407]
	Haliotisin P6	*H. tuberculata*	***B. subtilis*** 2-3 μM	P. carotovorum 0.5-0.8 μM		[407]
LBP/BPI	Cg-BPI (short-chain)	*C. gigas*	***E. coli*** 0.3 μM			[408]
	hBPI$_{21}$ (short-chain)	*C. gigas*	***E. coli*** 0.3 μM			[408]
	hBPI$_{21}$ (long-chain)	*C. gigas*	***E. coli*** 5 μM			[408]
Lysozyme	MyLysoG	*M. yessoensis*				[409]
Ubiquitin	CgUbiquitin	*C. gigas*	***B. subtilis*** 3.4 μM<br>***S. aureus*** 40 μM<br>***S. iniae*** 7.8 μM	***A. hydrophila*** 5.8 μM<br>***E. coli*** 1.9-12 μM<br>***P. aeruginosa*** 5 μM<br>***S. flexneri*** 5.6 μM<br>*S. sonnei* 10 μM<br>***V. parahaemolyticus*** 9.8 μM		[410]
Phylum Platyhelminthes						
Schistocin	Schistocin-1	*S. mansoni*	Fungi		***C. albicans*** 250 μM<br>***C. tropicalis***500 μM<br>*P. kudriavzevii* 125 μM	[411]
	Schistocin-2	*S. mansoni*	Fungi		***C. albicans*** 250 μM<br>***C. tropicalis*** 500 μM<br>*P. kudriavzevii* 62.5 μM	[411]
	Schistocin-3	*S. mansoni*	***S. aureus*** 31.2 μM	***E. coli*** 62.5 μM<br>***P. aeruginosa*** 62.5 μM	***C. albicans*** 62.5 μM<br>***C. parapsilosis*** 125 μM<br>***C. tropicalis*** 31.2 μM<br>*N. glabratus*250 μM<br>*P. kudriavzevii* 62.5 μM	[411]
	Schistocin-3.1	*S. mansoni*	***S. aureus*** 8 μM	***E. coli*** 62.5 μM<br>***P. aeruginosa*** 31.2 μM	***C. albicans*** 31.2 μM<br>***C. parapsilosis*** 125 μM<br>***C. tropicalis*** 31.2 μM<br>*N. glabratus* 125 μM<br>*P. kudriavzevii* 62.5 μM	[411]

Concentration ranges may reflect differences in strain susceptibility, peptide origin (native or synthetic), or variations in pH and salinity. Refer to the original source for more details. NV (No value) indicates that MIC or related metrics are not available; antimicrobial activity was confirmed through disk diffusion assay or gene expression analysis following an antimicrobial challenge. *Values represent the IC50.

Supplementary Material References

1. Rao, P.K. Pathological and Pathobiology of Epizootic Ulcerative Syndrome (EUS) Causing Aspergillus fumigatus and Its Im-munological Response in Freshwater Fish of Channa striatus. J. Fish. Sci. 2023, 17, 127.
2. Kwon-Chung, K. J.; Sugui, J. A. Aspergillus Fumigatus—What Makes the Species a Ubiquitous Human Fungal Pathogen? PLoS Pathog. 2013, 9 (12), e1003743. https://doi.org/10.1371/journal.ppat.1003743.
3. Olufemi, B. E.; Agius, C.; Roberts, R. J. Aspergillomycosis in Intensively Cultured Tilapia from Kenya. 1983, 203–204.
4. Erfandoust, R.; Habibipour, R.; Soltani, J. Antifungal Activity of Endophytic Fungi from Cupressaceae against Human Path-ogenic Aspergillus fumigatus and Aspergillus niger. J. Mycol. Med. 2020, 30 (3), 100987. https://doi.org/10.1016/j.mycmed.2020.100987.
5. Mahboub, H. H.; Nada, H. S.; Abdel-Ghany, H. M.; Ghanem, R.; Ahmed Ismail, T.; Abdel Rahman, A. N. Detection, Diagnosis, Koch's Postulate, Hepatorenal and Antioxidant Indicators for Some Systemic Pathogenic Fungi Invading the Liver and Kidneys of African Catfish (Clarias gariepinus) in Egypt with a Histopathological Approach. Aquac. Res. 2022, 53 (7), 2670–2685. https://doi.org/10.1111/are.15783.
6. Moazam, S.; Denning, D. W. Aspergillus Nodules in Chronic Granulomatous Disease Attributable to Aspergillus ochraceus. Med. Mycol. Case Rep. 2017, 17 (June), 31–33. https://doi.org/10.1016/j.mmcr.2017.06.004.
7. Shrivastava, A. Record of Aspergillus terreus (Thorn.) (Fungi) as Fish Pathogen. Indian J. Fish 1996, 43 (2), 203–204.
8. Arabatzis, M.; Velegraki, A. Sexual Reproduction in the Opportunistic Human Pathogen Aspergillus terreus. Mycologia 2013, 105 (1), 71–79. https://doi.org/10.3852/11-426.
9. Eissa, A. E.; Tharwat, N. A.; Zaki, M. M. Field Assessment of the Mid Winter Mass Kills of Trophic Fishes at Mariotteya Stream, Egypt: Chemical and Biological Pollution Synergistic Model. Chemosphere 2013, 90 (3), 1061–1068. https://doi.org/10.1016/j.chemosphere.2012.09.010.
10. Kim, J.; Sudbery, P. Candida albicans, a Major Human Fungal Pathogen. J. Microbiol. 2011, 49 (2), 171–177. https://doi.org/10.1007/s12275-011-1064-7.
11. Silva, S.; Negri, M.; Henriques, M.; Oliveira, R.; Williams, D. W.; Azeredo, J. Candida glabrata, Candida parapsilosis and Candida tropicalis : Biology, Epidemiology, Pathogenicity and Antifungal Resistance. FEMS Microbiol. Rev. 2012, 36 (2), 288–305. https://doi.org/10.1111/j.1574-6976.2011.00278.x.
12. Haridy, M.; Abdo, W.; Hashem, M.; Yanai, T. Candida parapsilosis and Candida tropicalis Infections in an Okhotsk Snailfish (Liparis ochotensis). J. Vet. Med. Sci. 2018, 80 (11), 1676–1680. https://doi.org/10.1292/jvms.18-0133.
13. Blaylock, R. B.; Overstreet, R. M.; Klich, M. A. Mycoses in Red Snapper (Lutjanus campechanus) Caused by Two Deuteromycete Fungi (Penicillium corylophilum and Cladosporium sphaerospermum)*. Hydrobiologia 2001, 460, 221–228.
14. Kaur, H.; Gupta, P.; Ahmad, H.; Shankarnarayan, S. A.; Srivastava, S.; Sahu, S.; Karuna, T.; Narang, T.; Gupta, S.; Ghosh, A.; Rudramurthy, S. M. Cladosporium halotolerans: Exploring an Unheeded Human Pathogen. Mycopathologia 2023, 188 (6), 1027–1040. https://doi.org/10.1007/s11046-023-00801-6.
15. Kulatunga, D. C. M.; Dananjaya, S. H. S.; Park, B. K.; Kim, C.; Lee, J.; De Zoysa, M. First Report of Fusarium oxysporum Species Complex Infection in Zebrafish Culturing System. J. Fish Dis. 2017, 40 (4), 485–494. https://doi.org/10.1111/jfd.12529.
16. Nucci, M.; Anaissie, E. Fusarium Infections in Immunocompromised Patients. Clin. Microbiol. Rev. 2007, 20 (4), 695–704. https://doi.org/10.1128/CMR.00014-07.
17. Yao, L.; Wang, C.; Li, G.; Xie, G.; Jia, Y.; Wang, W.; Liu, S.; Xu, T.; Luo, K.; Zhang, Q.; Kong, J. Identification of Fusarium solani as a Causal Agent of Black Spot Disease (BSD) of Pacific White Shrimp, Penaeus vannamei. Aquaculture 2022, 548 (106), 737602. https://doi.org/10.1016/j.aquaculture.2021.737602.

18. Dabas, Y.; Bakhshi, S.; Xess, I. Fatal Cases of Bloodstream Infection by Fusarium solani and Review of Published Literature. Mycopathologia 2016, 181 (3–4), 291–296. https://doi.org/10.1007/s11046-015-9960-8.
19. Behera, B. K.; Paria, P.; Das, A.; Bhowmick, S.; Sahoo, A. K.; Das, B. K. Molecular Characterization and Pathogenicity of a Virulent Acinetobacter Baumannii Associated with Mortality of Farmed Indian Major Carp Labeo rohita (Hamilton 1822). Aquaculture 2017, 471, 157–162. https://doi.org/10.1016/j.aquaculture.2017.01.018.
20. Harding, C. M.; Hennon, S. W.; Feldman, M. F. Uncovering the Mechanisms of Acinetobacter baumannii Virulence. Nat. Rev. Microbiol. 2018, 16 (2), 91–102. https://doi.org/10.1038/nrmicro.2017.148.
21. Semwal, A.; Kumar, A.; Kumar, N. A Review on Pathogenicity of Aeromonas hydrophila and Their Mitigation through Medicinal Herbs in Aquaculture. Heliyon 2023, 9 (3), e14088. https://doi.org/10.1016/j.heliyon.2023.e14088.
22. Davis, W. A.; Kane, J. G.; Garagusi, V. F. Human Aeromonas Infections: A Review of the Literature and a Case Report of En-docarditis. Medicine (Baltimore). 1978, 57 (3), 267–277.
23. Behera, B. K.; Paria, P.; Das, A.; Das, B. K. Molecular Identification and Pathogenicity Study of Virulent Citrobacter freundii Associated with Mortality of Farmed Labeo rohita (Hamilton 1822), in India. Aquaculture 2022, 547 (August 2021), 737437. https://doi.org/10.1016/j.aquaculture.2021.737437.
24. Liu, L.-H.; Wang, N.-Y.; Wu, A. Y.-J.; Lin, C.-C.; Lee, C.-M.; Liu, C.-P. Citrobacter freundii Bacteremia: Risk Factors of Mortality and Prevalence of Resistance Genes. J. Microbiol. Immunol. Infect. 2018, 51 (4), 565–572. https://doi.org/10.1016/j.jmii.2016.08.016.
25. Loch, T. P.; Hawke, J. P.; Reichley, S. R.; Faisal, M.; Del Piero, F.; Griffin, M. J. Outbreaks of Edwardsiellosis Caused by Ed-wardsiella piscicida and Edwardsiella tarda in Farmed Barramundi (Lates calcarifer). Aquaculture 2017, 481 (August), 202–210. https://doi.org/10.1016/j.aquaculture.2017.09.005.
26. Wang, I.-K.; Kuo, H.-L.; Chen, Y.-M.; Lin, C.-L.; Chang, H.-Y.; Chuang, F.-R.; Lee, M.-H. Extraintestinal Manifestations of Edwardsiella Tarda Infection. Int. J. Clin. Pract. 2005, 59 (8), 917–921. https://doi.org/10.1111/j.1742-1241.2005.00527.x.
27. Gao, X.; Zhang, H.; Jiang, Q.; Chen, N.; Li, X.; Liu, X.; Yang, H.; Wei, W.; Zhang, X. Enterobacter Cloacae Associated with Mass Mortality in Zoea of Giant Freshwater Prawns Macrobrachium Rosenbergii and Control with Specific Chicken Egg Yolk Im-munoglobulins (IgY). Aquaculture 2019, 501 (November 2018), 331–337. https://doi.org/10.1016/j.aquaculture.2018.11.050.
28. Davin-Regli, A.; PagÃ¨s, J.-M. Enterobacter Aerogenes and Enterobacter Cloacae; Versatile Bacterial Pathogens Confronting Antibiotic Treatment. Front. Microbiol. 2015, 6 (MAY), 1–10. https://doi.org/10.3389/fmicb.2015.00392.
29. Dang, S. T. T.; Dalsgaard, A. Escherichia Coli Contamination of Fish Raised in Integrated Pig-Fish Aquaculture Systems in Vietnam. J. Food Prot. 2012, 75 (7), 1317–1319. https://doi.org/10.4315/0362-028X.JFP-11-501.
30. Ferens, W. A.; Hovde, C. J. Escherichia Coli O157:H7: Animal Reservoir and Sources of Human Infection. Foodborne Pathog. Dis. 2011, 8 (4), 465–487. https://doi.org/10.1089/fpd.2010.0673.
31. Cao, H.; An, J.; Ou, R.; Lu, L.; Ai, X.; Yang, Y. Enterobacter Aerogenes: An Emerging Pathogen for Enteritis in Farmed Channel Catfish Ictalurus Punctatus. Isr. J. Aquac. - Bamidgeh 2017, 69 (1). https://doi.org/10.46989/001c.21063.
32. Álvarez-Marín, R.; Lepe, J. A.; Gasch-Blasi, O.; Rodríguez-Martínez, J. M.; Calvo-Montes, J.; Lara-Contreras, R.; Mar-tín-Gandul, C.; Tubau-Quintano, F.; Cano-García, M. E.; Rodríguez-López, F.; Rodríguez-Baño, J.; Pujol-Rojo, M.; Tor-re-Cisneros, J.; Martínez-Martínez, L.; Pascual-Hernández, Á.; Jiménez-Mejías, M. E. Clinical Characteristics and Outcome of Bacteraemia Caused by Enterobacter Cloacae and Klebsiella Aerogenes: More Similarities than Differences. J. Glob. Antimicrob. Resist. 2021, 25, 351–358. https://doi.org/10.1016/j.jgar.2021.04.008.
33. Cai, J.; Wang, Z.; Cai, C.; Zhou, Y. Characterization and Identification of Virulent Klebsiella Oxytoca Isolated from Abalone (Haliotis Diversicolor Supertexta) Postlarvae with Mass Mortality in Fujian, China. J. Invertebr. Pathol. 2008, 97 (1), 70–75. https://doi.org/10.1016/j.jip.2007.07.005.
34. Neog, N.; Phukan, U.; Puzari, M.; Sharma, M.; Chetia, P. Klebsiella Oxytoca and Emerging Nosocomial Infections. Curr. Microbiol. 2021, 78 (4), 1115–1123. https://doi.org/10.1007/s00284-021-02402-2.
35. Das, A.; Behera, B. K.; Acharya, S.; Paria, P.; Chakraborty, H. J.; Parida, P. K.; Das, B. K. Genetic Diversity and Multiple An-tibiotic Resistance Index Study of Bacterial Pathogen, Klebsiella Pneumoniae Strains Isolated from Diseased Indian Major Carps. Folia Microbiol. (Praha). 2019, 64 (6), 875–887. https://doi.org/10.1007/s12223-019-00701-7.
36. Bengoechea, J. A.; Sa Pessoa, J. Klebsiella Pneumoniae Infection Biology: Living to Counteract Host Defences. FEMS Microbiol. Rev. 2019, 43 (2), 123–144. https://doi.org/10.1093/femsre/fuy043.
37. Rivas, A. J.; Lemos, M. L.; Osorio, C. R. Photobacterium Damselae Subsp. Damselae, a Bacterium Pathogenic for Marine An-imals and Humans. Front. Microbiol. 2013, 4 (SEP), 1–6. https://doi.org/10.3389/fmicb.2013.00283.
38. Pattanayak, S.; Kumar, P. R.; Sahoo, M. K.; Paul, A.; Sahoo, P. K. First Field-Based Evidence of Association of Proteus Mirabilis Causing Large Scale Mortality in Indian Major Carp Farming. Aquaculture 2018, 495 (February), 435–442. https://doi.org/10.1016/j.aquaculture.2018.06.006.
39. Armbruster, C. E.; Mobley, H. L. T.; Pearson, M. M. Pathogenesis of Proteus Mirabilis Infection. EcoSal Plus 2018, 8 (1). https://doi.org/10.1128/ecosalplus.esp-0009-2017.
40. Sun, L.; Sun, Y.; Jiang, M.; Luo, L.; Yu, X.; Yao, W.; Wu, Z. Isolation, Identification and Pathogenicity of Proteus Vulgaris from Moribund Common Carp (Cyprinus Carpio) Farmed in China. Aquaculture 2020, 525 (2), 735294. https://doi.org/10.1016/j.aquaculture.2020.735294.
41. Kwil, I.; Kaźmierczak, D.; Różalski, A. Swarming Growth and Resistance of Proteus Penneri and Proteus Vulgaris Strains to Normal Human Serum. Adv. Clin. Exp. Med. 2013, 22 (2), 165–175.
42. Thomas, J.; Thanigaivel, S.; Vijayakumar, S.; Acharya, K.; Shinge, D.; Seelan, T. S. J.; Mukherjee, A.; Chandrasekaran, N. Pathogenecity of Pseudomonas Aeruginosa in Oreochromis Mossambicus and Treatment Using Lime Oil Nanoemulsion. Col-loids Surfaces B Biointerfaces 2014, 116, 372–377. https://doi.org/10.1016/j.colsurfb.2014.01.019.
43. Lyczak, J. B.; Cannon, C. L.; Pier, G. B. Establishment of Pseudomonas Aeruginosa Infection: Lessons from a Versatile Op-portunist. Microbes Infect. 2000, 2 (9), 1051–1060. https://doi.org/10.1016/S1286-4579(00)01259-4.
44. Altinok, I.; Kayis, S.; Capkin, E. Pseudomonas Putida Infection in Rainbow Trout. Aquaculture 2006, 261 (3), 850–855. https://doi.org/10.1016/j.aquaculture.2006.09.009.
45. Yoshino, Y.; Kitazawa, T.; Kamimura, M.; Tatsuno, K.; Yotsuyanagi, H.; Ota, Y. Pseudomonas Putida Bacteremia in Adult Patients: Five Case Reports and a Review of the Literature. J. Infect. Chemother. 2011, 17 (2), 278–282. https://doi.org/10.1007/s10156-010-0114-0.
46. Emam, A. M.; Haridy, M.; Hossam Eldin Ahmed, N. Pathogenicity of Newly Emerged Bacterial Pathogens, Pseudomonas Stutzeri and P. Oleovorans, in the Red Sea Seabream Diplodus Noct. Egypt. J. Aquat. Res. 2022, 48 (2), 169–174. https://doi.org/10.1016/j.ejar.2022.02.001.
47. Alwazzeh, M. J.; Alkuwaiti, F. A.; Alqasim, M.; Alwarthan, S.; El-ghoneimy, Y. Infective Endocarditis Caused by Pseudomonas Stutzeri: A Case Report and Literature Review. Infect. Dis. Rep. 2020, 12 (3), 105–109. https://doi.org/10.3390/idr12030020.
48. Kodama, H.; Nakanishi, Y.; Yamamoto, F.; Mikami, T.; Izawa, H.; Imagawa, T.; Hashimoto, Y.; Kudo, N. Salmonella Arizonae Isolated from a Pirarucu, Arapaima Gigas Cuvier, with Septicaemia. J. Fish Dis. 1987, 10 (6).
49. Chiu, C.-H.; Chuang, C.-H.; Chiu, S.; Su, L.-H.; Lin, T.-Y. Salmonella Enterica Serotype Choleraesuis Infections in Pediatric Patients. Pediatrics 2006, 117 (6), e1193–e1196. https://doi.org/10.1542/peds.2005-2251.
50. dos Santos, R. R.; Xavier, R. G. C.; de Oliveira, T. F.; Leite, R. C.; Figueiredo, H. C. P.; Leal, C. A. G. Occurrence, Genetic Di-versity, and Control of Salmonella Enterica in Native Brazilian Farmed Fish. Aquaculture 2019, 501 (May 2018), 304–312. https://doi.org/10.1016/j.aquaculture.2018.11.034.

51. Calarga, A. P.; Gontijo, M. T. P.; de Almeida, L. G. P.; de Vasconcelos, A. T. R.; Nascimento, L. C.; de Moraes Barbosa, T. M. C.; de Carvalho Perri, T. M.; dos Santos, S. R.; Tiba-Casas, M. R.; Marques, E. G. L.; Ferreira, C. M.; Brocchi, M. Antimicrobial Resistance and Genetic Background of Non-Typhoidal Salmonella Enterica Strains Isolated from Human Infections in São Paulo, Brazil (2000–2019). Brazilian J. Microbiol. 2022, 53 (3), 1249–1262. https://doi.org/10.1007/s42770-022-00748-8.
52. Austin, B.; Austin, D. A. Bacterial Fish Pathogens, 6th editio.; Springer International Publishing: Cham, 2016. https://doi.org/10.1007/978-3-319-32674-0.
53. Helms, M.; Ethelberg, S.; Mølbak, K.; Group, D. S. International Salmonella Typhimurium DT104 Infections, 1992–2001. Emerg. Infect. Dis. 2005, 11 (6), 859–867.
54. Dharmaratnam, A.; Kumar, R.; Basheer, V. S.; Sood, N.; Swaminathan, T. R.; Jena, J. K. Isolation and Characterisation of Virulent Serratia Marcescens Associated with a Disease Outbreak in Farmed Ornamental Fish, Poecilia Reticulata in Kerala, India. Indian J. Fish. 2017, 64 (4), 71–79. https://doi.org/10.21077/ijf.2017.64.4.71261-10.
55. Zivkovic Zaric, R.; Zaric, M.; Sekulic, M.; Zornic, N.; Nesic, J.; Rosic, V.; Vulovic, T.; Spasic, M.; Vuleta, M.; Jovanovic, J.; Jovanovic, D.; Jakovljevic, S.; Canovic, P. Antimicrobial Treatment of Serratia Marcescens Invasive Infections: Systematic Re-view. Antibiotics 2023, 12 (2), 367. https://doi.org/10.3390/antibiotics12020367.
56. Cao, H.; Chen, S.; Lu, L.; An, J. Shewanella Algae: An Emerging Pathogen of Black Spot Disease in Freshwater-Cultured Whiteleg Shrimp (Penaeus Vannamei),. Isr. J. Aquac. - Bamidgeh 2018, 70 (January). https://doi.org/10.46989/001c.20952.
57. Goyal, R.; Kaur, N.; Thakur, R. Human Soft Tissue Infection by the Emerging Pathogen Shewanella Algae. J. Infect. Dev. Ctries. 2011, 5 (04), 310–312. https://doi.org/10.3855/jidc.1436.
58. Kousar, R.; Shafi, N.; Andleeb, S.; Ali, N. M.; Akhtar, T.; Khalid, S. Assessment and Incidence of Fish Associated Bacterial Pathogens at Hatcheries of Azad Kashmir, Pakistan. Brazilian J. Biol. 2020, 80 (3), 607–614. https://doi.org/10.1590/1519-6984.217435.
59. Jennison, A. V.; Verma, N. K. Shigella Flexneri Infection: Pathogenesis and Vaccine Development. FEMS Microbiol. Rev. 2004, 28 (1), 43–58. https://doi.org/10.1016/j.femsre.2003.07.002.
60. Selvin, J.; Lipton, A. Vibrio Alginolyticus Associated with White Spot Disease of Penaeus Monodon. Dis. Aquat. Organ. 2003, 57 (1–2), 147–150. https://doi.org/10.3354/dao057147.
61. Jacobs Slifka, K. M.; Newton, A. E.; Mahon, B. E. Vibrio Alginolyticus Infections in the USA, 1988–2012. Epidemiol. Infect. 2017, 145 (7), 1491–1499. https://doi.org/10.1017/S0950268817000140.
62. Frans, I.; Michiels, C. W.; Bossier, P.; Willems, K. A.; Lievens, B.; Rediers, H. Vibrio Anguillarum as a Fish Pathogen: Virulence Factors, Diagnosis and Prevention. J. Fish Dis. 2011, 34 (9), 643–661. https://doi.org/10.1111/j.1365-2761.2011.01279.x.
63. Sinatra, J. A.; Colby, K. Notes from the Field: Fatal Vibrio Anguillarum Infection in an Immunocompromised Patient — Maine, 2017. MMWR. Morb. Mortal. Wkly. Rep. 2018, 67 (34), 962–963. https://doi.org/10.15585/mmwr.mm6734a5.
64. Sullivan, T. J.; Neigel, J. E. Effects of Temperature and Salinity on Prevalence and Intensity of Infection of Blue Crabs, Callinectes Sapidus, by Vibrio Cholerae, V. Parahaemolyticus, and V. Vulnificus in Louisiana. J. Invertebr. Pathol. 2018, 151 (September 2017), 82–90. https://doi.org/10.1016/j.jip.2017.11.004.
65. Cottingham, K. L.; Chiavelli, D. A.; Taylor, R. K. Environmental Microbe and Human Pathogen: The Ecology and Microbiology of Vibrio Cholerae. Front. Ecol. Environ. 2003, 1 (2), 80. https://doi.org/10.2307/3868034.
66. Kumara, K. R. P. S.; Hettiarachchi, M. White Faeces Syndrome Caused by Vibrio Alginolyticus and Vibrio Fluvialis in Shrimp, Penaeus Monodon (Fabricius 1798) - Multimodal Strategy to Control the Syndrome in Sri Lankan Grow-out Ponds. Asian Fish. Sci. 2017, 30 (4), 245–261. https://doi.org/10.33997/j.afs.2017.30.4.003.
67. Ramamurthy, T.; Chowdhury, G.; Pazhani, G. P.; Shinoda, S. Vibrio Fluvialis: An Emerging Human Pathogen. Front. Microbiol. 2014, 5 (MAR), 1–8. https://doi.org/10.3389/fmicb.2014.00091.
68. Zhang, X.-H.; He, X.; Austin, B. Vibrio Harveyi: A Serious Pathogen of Fish and Invertebrates in Mariculture. Mar. Life Sci. Technol. 2020, 2 (3), 231–245. https://doi.org/10.1007/s42995-020-00037-z.
69. Del Gigia-Aguirre, L.; Sánchez-Yebra-Romera, W.; García-Muñoz, S.; Rodríguez-Maresca, M. First Description of Wound Infection with Vibrio Harveyi in Spain. New Microbes New Infect. 2017, 19 (April), 15–16. https://doi.org/10.1016/j.nmni.2017.05.004.
70. Siddique, A. B.; Moniruzzaman, M.; Ali, S.; Dewan, M. N.; Islam, M. R.; Islam, M. S.; Amin, M. B.; Mondal, D.; Parvez, A. K.; Mahmud, Z. H. Characterization of Pathogenic Vibrio Parahaemolyticus Isolated From Fish Aquaculture of the Southwest Coastal Area of Bangladesh. Front. Microbiol. 2021, 12 (March). https://doi.org/10.3389/fmicb.2021.635539.
71. Wang, R.; Zhong, Y.; Gu, X.; Yuan, J.; Saeed, A. F.; Wang, S. The Pathogenesis, Detection, and Prevention of Vibrio Para-haemolyticus. Front. Microbiol. 2015, 6 (MAR), 1–13. https://doi.org/10.3389/fmicb.2015.00144.
72. Tey, Y. H.; Jong, K.-J.; Fen, S.-Y.; Wong, H.-C. Occurrence of Vibrio Parahaemolyticus, Vibrio Cholerae, and Vibrio Vulnificus in the Aquacultural Environments of Taiwan. J. Food Prot. 2015, 78 (5), 969–976. https://doi.org/10.4315/0362-028X.JFP-14-405.
73. Baker ‐ Austin, C.; Oliver, J. D. Vibrio Vulnificus : New Insights into a Deadly Opportunistic Pathogen. Environ. Microbiol. 2018, 20 (2), 423–430. https://doi.org/10.1111/1462-2920.13955.
74. Zamora, J.; Enriquez, R. Yersinia Enterocolitica, Yersinia Frederiksenii. 1987, 159, 155–159.
75. Fredriksson-Ahomaa, M.; Stolle, A.; Korkeala, H. Molecular Epidemiology of Yersinia Enterocolitica Infections. FEMS Immunol. Med. Microbiol. 2006, 47 (3), 315–329. https://doi.org/10.1111/j.1574-695X.2006.00095.x.
76. Ke, X.; Lu, M.; Ye, X.; Gao, F.; Zhu, H.; Huang, Z. Recovery and Pathogenicity Analysis of Aerococcus Viridans Isolated from Tilapia (Orecohromis Niloticus) Cultured in Southwest of China. Aquaculture 2012, 342–343 (1), 18–23. https://doi.org/10.1016/j.aquaculture.2012.02.012.
77. Mohan, B. Aerococcus Viridans : A Rare Pathogen Causing Urinary Tract Infection. J. Clin. DIAGNOSTIC Res. 2017, 11 (1), DR01–DR03. https://doi.org/10.7860/JCDR/2017/23997.9229.
78. Chandra, G.; Bhattacharjee, I.; Chatterjee, S. Bacillus Cereus Infection in Stinging Catfish, Heteropneustes Fossilis (Siluriformes: Heteropneustidae) and Their Recovery by Argemone Mexicana Seed Extract. Iran. J. Fish. Sci. 2015, 14 (3), 741–753.
79. Bottone, E. J. Bacillus Cereus , a Volatile Human Pathogen. Clin. Microbiol. Rev. 2010, 23 (2), 382–398. https://doi.org/10.1128/CMR.00073-09.
80. Orozova, P.; Sirakov, I.; Austin, D. A.; Austin, B. Recovery of Bacillus Mycoides, B. Pseudomycoides and Aeromonas Hy-drophila from Common Carp (Cyprinus Carpio) and Rainbow Trout (Oncorhynchus Mykiss) with Gill Disease. J. Fish Dis. 2018, 41 (1), 125–129. https://doi.org/10.1111/jfd.12686.
81. Heidt, J.; Papaloukas, N.; Timmerman, C. P. A Rare Bloodstream Infection: Bacillus Mycoides. Neth. J. Med. 2019, 77 (6), 227–230.
82. Pychynski, T.; Malanowska, T.; Kozlowski, M. Bacterial Flora in Branchionecrosis of Carp (Particularly Bacillus Cereus and Bacillus Subtilis). 1981.
83. Tsonis, I.; Karamani, L.; Xaplanteri, P.; Kolonitsiou, F.; Zampakis, P.; Gatzounis, G.; Marangos, M.; Assimakopoulos, S. F. Spontaneous Cerebral Abscess Due to Bacillus Subtilis in an Immunocompetent Male Patient: A Case Report and Review of Literature. World J. Clin. Cases 2018, 6 (16), 1169–1174. https://doi.org/10.12998/wjcc.v6.i16.1169.
84. Sabry, M.; Abd El-Moein, K.; Hamza, E.; Abdel Kader, F. Occurrence of Clostridium Perfringens Types A, E, and C in Fresh Fish and Its Public Health Significance. J. Food Prot. 2016, 79 (6), 994–1000. https://doi.org/10.4315/0362-028X.JFP-15-569.

85. Kiu, R.; Hall, L. J. An Update on the Human and Animal Enteric Pathogen Clostridium Perfringens. Emerg. Microbes Infect. 2018, 7 (1), 1–15. https://doi.org/10.1038/s41426-018-0144-8.
86. Akter, T.; Haque, M. N.; Ehsan, R.; Paul, S. I.; Foysal, M. J.; Tay, A. C. Y.; Islam, M. T.; Rahman, M. M. Virulence and Antibi-otic-Resistance Genes in Enterococcus Faecalis Associated with Streptococcosis Disease in Fish. Sci. Rep. 2023, 13 (1), 1551. https://doi.org/10.1038/s41598-022-25968-8.
87. Abat, C.; Huart, M.; Garcia, V.; Dubourg, G.; Raoult, D. Enterococcus Faecalis Urinary-Tract Infections: Do They Have a Zoonotic Origin? J. Infect. 2016, 73 (4), 305–313. https://doi.org/10.1016/j.jinf.2016.07.012.
88. Chen, S.; Chen, T.; Wang, P.; Chen, Y.; Huang, J.; Lin, Y.; Chaung, H. Metschnikowia Bicuspidata and Enterococcus Faecium Co-Infection in the Giant Freshwater Prawn Macrobrachium Rosenbergii. Dis. Aquat. Organ. 2003, 55, 161–167. https://doi.org/10.3354/dao055161.
89. Zhou, X.; Willems, R. J. L.; Friedrich, A. W.; Rossen, J. W. A.; Bathoorn, E. Enterococcus Faecium: From Microbiological Insights to Practical Recommendations for Infection Control and Diagnostics. Antimicrob. Resist. Infect. Control 2020, 9 (1), 130. https://doi.org/10.1186/s13756-020-00770-1.
90. Pękala, A.; Paździor, E.; Antychowicz, J.; Bernad, A.; Głowacka, H.; Więcek, B.; Niemczuk, W. Kocuria Rhizophila and Mi-crococcus Luteus as Emerging Opportunist Pathogens in Brown Trout (Salmo Trutta Linnaeus, 1758) and Rainbow Trout (Oncorhynchus Mykiss Walbaum, 1792). Aquaculture 2018, 486 (December 2017), 285–289. https://doi.org/10.1016/j.aquaculture.2017.12.028.
91. Moissenet, D.; Becker, K.; Mérens, A.; Ferroni, A.; Dubern, B.; Vu-Thien, H. Persistent Bloodstream Infection with Kocuria Rhizophila Related to a Damaged Central Catheter. J. Clin. Microbiol. 2012, 50 (4), 1495–1498. https://doi.org/10.1128/JCM.06038-11.
92. Meyburgh, C.; Bragg, R.; Boucher, C. Lactococcus Garvieae: An Emerging Bacterial Pathogen of Fish. Dis. Aquat. Organ. 2017, 123 (1), 67–79. https://doi.org/10.3354/dao03083.
93. Li, W.-K.; Chen, Y.-S.; Wann, S.-R.; Liu, Y.-C.; Tsai, H.-C. Lactococcus Garvieae Endocarditis with Initial Presentation of Acute Cerebral Infarction in a Healthy Immunocompetent Man. Intern. Med. 2008, 47 (12), 1143–1146. https://doi.org/10.2169/internalmedicine.47.0795.
94. Wang, P.; Lin, Y.; Liaw, L.; Chern, R.; Chen, S. Lactococcus Lactis Subspecies Lactis Also Causes White Muscle Disease in Farmed Giant Freshwater Prawns Macrobrachium Rosenbergii. Dis. Aquat. Organ. 2008, 79 (1), 9–17. https://doi.org/10.3354/dao01868.
95. Lahlou, W.; Bourial, A.; Maaouni, T.; Bensaad, A.; Bensahi, I.; Sabry, M.; Miguil, M. Lactococcus Lactis Endocarditis and Liver Abscess in an Immunocompetent Patient: A Case Report and Review of the Literature. J. Med. Case Rep. 2023, 17 (1), 115. https://doi.org/10.1186/s13256-022-03676-1.
96. Seifert, H.; Kaltheuner, M.; Perdreau-Remington, F. Micrococcus Luteus Endocarditis: Case Report and Review of the Litera-ture. Zentralblatt für Bakteriol. 1995, 282 (4), 431–435. https://doi.org/10.1016/S0934-8840(11)80715-2.
97. Talaat, A. M.; Trucksis, M.; Kane, A. S.; Reimschuessel, R. Pathogenicity of Mycobacterium Fortuitum and Mycobacterium Smegmatis to Goldfish, Carassius Auratus. Vet. Microbiol. 1999, 66 (2), 151–164. https://doi.org/10.1016/S0378-1135(99)00002-4.
98. Wallace, R. J.; Nash, D. R.; Tsukamura, M.; Blacklock, Z. M.; Silcox, V. A. Human Disease Due to Mycobacterium Smegmatis. J. Infect. Dis. 1988, 158 (1), 52–59. https://doi.org/10.1093/infdis/158.1.52.
99. Avendaño-Herrera, R.; Balboa, S.; Doce, A.; Ilardi, P.; Lovera, P.; Toranzo, A. E.; Romalde, J. L. Pseudo-Membranes on Internal Organs Associated with Rhodococcus Qingshengii Infection in Atlantic Salmon (Salmo Salar). Vet. Microbiol. 2011, 147 (1–2), 200–204. https://doi.org/10.1016/j.vetmic.2010.06.003.
100. Kitamura, Y.; Sawabe, E.; Ohkusu, K.; Tojo, N.; Tohda, S. First Report of Sepsis Caused by Rhodococcus Corynebacterioides in a Patient with Myelodysplastic Syndrome. J. Clin. Microbiol. 2012, 50 (3), 1089–1091. https://doi.org/10.1128/JCM.06279-11.
101. Çanak, Ö.; Timur, G. An Initial Survey on the Occurrence of Staphylococcal Infections in Turkish Marine Aquaculture (2013–1014). J. Appl. Ichthyol. 2020, 36 (6), 932–941. https://doi.org/10.1111/jai.14141.
102. Bouiller, K.; Bertrand, X.; Hocquet, D.; Chirouze, C. Human Infection of Methicillin-Susceptible Staphylococcus Aureus CC398: A Review. Microorganisms 2020, 8 (11), 1737. https://doi.org/10.3390/microorganisms8111737.
103. Patel, J. K.; Zwetlana, A.; Ghosh, D.; Rajan, V.; Sivaraman, G. K.; Vivekanandan, P.; Elangovan, R. Whole Genome Charac-terization of Methicillin-Resistant Staphylococcus Spp. Isolates from Aquaculture-Cultivated Shrimps. Aquaculture 2023, 575 (June), 739704. https://doi.org/10.1016/j.aquaculture.2023.739704.
104. Nguyen, T. H.; Park, M. D.; Otto, M. Host Response to Staphylococcus Epidermidis Colonization and Infections. Front. Cell. Infect. Microbiol. 2017, 7 (MAR), 1–7. https://doi.org/10.3389/fcimb.2017.00090.
105. Medina-Félix, D.; Vargas-Albores, F.; Garibay-Valdez, E.; Martínez-Córdova, L. R.; Martínez-Porchas, M. Oreochromis Nilot-icus Gastrointestinal Microbiota Affected by the Infection with Staphylococcus Haemolyticus and Providencia Vermicola, Two Emerging Pathogens in Fish Aquaculture. Aquaculture 2024, 582 (June 2023), 740529. https://doi.org/10.1016/j.aquaculture.2023.740529.
106. Eltwisy, H. O.; Twisy, H. O.; Hafez, M. H. R.; Sayed, I. M.; El-Mokhtar, M. A. Clinical Infections, Antibiotic Resistance, and Pathogenesis of Staphylococcus Haemolyticus. Microorganisms 2022, 10 (6), 1130. https://doi.org/10.3390/microorganisms10061130.
107. Wu, Y.; Feng, Y.; Xue, M.; Xiao, Z.; Jin, L.; Gao, R.; Chen, Y.; Liang, T.; Zhou, Y. Isolation and Identification of Staphylococcus Saprophyticus from Diseased Hybrid Sturgeon. Fishes 2023, 8 (5), 250. https://doi.org/10.3390/fishes8050250.
108. Lawal, O. U.; Fraqueza, M. J.; Bouchami, O.; Worning, P.; Bartels, M. D.; Gonçalves, M. L.; Paixão, P.; Gonçalves, E.; Toscano, C.; Empel, J.; Urbaś, M.; Domínguez, M. A.; Westh, H.; de Lencastre, H.; Miragaia, M. Foodborne Origin and Local and Global Spread of Staphylococcus Saprophyticus Causing Human Urinary Tract Infections. Emerg. Infect. Dis. 2021, 27 (3), 880–893. https://doi.org/10.3201/eid2703.200852.
109. Tavares, G. C.; de Queiroz, G. A.; Assis, G. B. N.; Leibowitz, M. P.; Teixeira, J. P.; Figueiredo, H. C. P.; Leal, C. A. G. Disease Outbreaks in Farmed Amazon Catfish (Leiarius Marmoratus x Pseudoplatystoma Corruscans) Caused by Streptococcus Agalactiae, S. Iniae, and S. Dysgalactiae. Aquaculture 2018, 495 (April), 384–392. https://doi.org/10.1016/j.aquaculture.2018.06.027.
110. Paveenkittiporn, W.; Ungcharoen, R.; Kerdsin, A. Streptococcus Agalactiae Infections and Clinical Relevance in Adults, Thailand. Diagn. Microbiol. Infect. Dis. 2020, 97 (1), 115005. https://doi.org/10.1016/j.diagmicrobio.2020.115005.
111. Miller, J. D.; Neely, M. N. Large-Scale Screen Highlights the Importance of Capsule for Virulence in the Zoonotic Pathogen Streptococcus Iniae. Infect. Immun. 2005, 73 (2), 921–934. https://doi.org/10.1128/IAI.73.2.921-934.2005.
112. Nho, S.-W.; Shin, G.-W.; Park, S.-B.; Jang, H.-B.; Cha, I.-S.; Ha, M.-A.; Kim, Y.-R.; Park, Y.-K.; Dalvi, R. S.; Kang, B.-J.; Joh, S.-J.; Jung, T.-S. Phenotypic Characteristics of Streptococcus Iniae and Streptococcus Parauberis Isolated from Olive Flounder (Paralichthys Olivaceus). FEMS Microbiol. Lett. 2009, 293 (1), 20–27. https://doi.org/10.1111/j.1574-6968.2009.01491.x.
113. Huan, S. J. K. W.; Tan, J. S. W.; Chin, A. Y. H. Streptococcus Parauberis Infection of the Hand. J. Hand Surg. (European Vol. 2021, 46 (1), 83–84. https://doi.org/10.1177/1753193420938504.
114. Dinh-Hung, N.; Dong, H. T.; Taengphu, S.; Soontara, C.; Rodkhum, C.; Senapin, S.; Chatchaiphan, S. Streptococcus Suis Is a Lethal Pathogen in Snakeskin Gourami, Trichopodus Pectoralis. Aquaculture 2023, 566 (December 2022), 739173. https://doi.org/10.1016/j.aquaculture.2022.739173.
115. Rayanakorn, A.; Goh, B.-H.; Lee, L.-H.; Khan, T. M.; Saokaew, S. Risk Factors for Streptococcus Suis Infection: A Systematic Review and Meta-Analysis. Sci. Rep. 2018, 8 (1), 13358. https://doi.org/10.1038/s41598-018-31598-w.
116. Pavić, D.; Grbin, D.; Hudina, S.; Prosenc Zmrzljak, U.; Miljanović, A.; Košir, R.; Varga, F.; Ćurko, J.; Marčić, Z.; Bielen, A. Tracing the Oomycete Pathogen Saprolegnia Parasitica in Aquaculture and the Environment. Sci. Rep. 2022, 12 (1), 16646. https://doi.org/10.1038/s41598-022-16553-0.
117. Dallaire-Dufresne, S.; Tanaka, K. H.; Trudel, M. V.; Lafaille, A.; Charette, S. J. Virulence, Genomic Features, and Plasticity of Aeromonas Salmonicida Subsp. Salmonicida, the Causative Agent of Fish Furunculosis. Vet. Microbiol. 2014, 169 (1–2), 1–7. https://doi.org/10.1016/j.vetmic.2013.06.025.

118. López, J.; Lorenzo, L.; Alcantara, R.; Navas, J. Characterization of Aliivibrio Fischeri Strains Associated with Disease Outbreak in Brill Scophthalmus Rhombus. Dis. Aquat. Organ. 2017, 124 (3), 215–222. https://doi.org/10.3354/dao03123.
119. Benediktsdóttir; Helgason; Sigurjónsdóttir. Vibrio Spp. Isolated from Salmonids with Shallow Skin Lesions and Reared at Low Temperature. J. Fish Dis. 1998, 21 (1), 19–28. https://doi.org/10.1046/j.1365-2761.1998.00065.x.
120. Attia, M. M.; Abdelsalam, M.; Elgendy, M. Y.; Sherif, A. H. Dactylogyrus Extensus and Pseudomonas Fluorescens Dual Infection in Farmed Common Carp (Cyprinus Carpio). Microb. Pathog. 2022, 173 (PA), 105867. https://doi.org/10.1016/j.micpath.2022.105867.
121. Garnier, M.; Labreuche, Y.; Nicolas, J.-L. Molecular and Phenotypic Characterization of Vibrio Aestuarianus Subsp. Francensis Subsp. Nov., a Pathogen of the Oyster Crassostrea Gigas. Syst. Appl. Microbiol. 2008, 31 (5), 358–365. https://doi.org/10.1016/j.syapm.2008.06.003.
122. Rojas, R.; Miranda, C. D.; Romero, J.; Barja, J. L.; Dubert, J. Isolation and Pathogenic Characterization of Vibrio Bivalvicida Associated With a Massive Larval Mortality Event in a Commercial Hatchery of Scallop Argopecten Purpuratus in Chile. Front. Microbiol. 2019, 10 (MAY), 1–13. https://doi.org/10.3389/fmicb.2019.00855.
123. Wang, L.; Chen, Y.; Huang, H.; Huang, Z.; Chen, H.; Shao, Z. Isolation and Identification of Vibrio Campbellii as a Bacterial Pathogen for Luminous Vibriosis of Litopenaeus Vannamei. Aquac. Res. 2015, 46 (2), 395–404. https://doi.org/10.1111/are.12191.
124. Li, R.; Dang, H.; Huang, Y.; Quan, Z.; Jiang, H.; Zhang, W.; Ding, J. Vibrio Coralliilyticus as an Agent of Red Spotting Disease in the Sea Urchin Strongylocentrotus Intermedius. Aquac. Reports 2020, 16 (October 2019), 100244. https://doi.org/10.1016/j.aqrep.2019.100244.
125. Ishimaru, K.; Akagawa-Matsushita, M.; Muroga, K. Vibrio Ichthyoenteri Sp. Nov., a Pathogen of Japanese Flounder (Paralichthys Olivaceus) Larvae. Int. J. Syst. Bacteriol. 1996, 46 (1), 155–159. https://doi.org/10.1099/00207713-46-1-155.
126. Goarant, C.; Ansquer, D.; Herlin, J.; Domalain, D.; Imbert, F.; De Decker, S. "Summer Syndrome" in Litopenaeus Stylirostris in New Caledonia: Pathology and Epidemiology of the Etiological Agent, Vibrio Nigripulchritudo. Aquaculture 2006, 253 (1–4), 105–113. https://doi.org/10.1016/j.aquaculture.2005.07.031.
127. Schiewe, M. H.; Trust, T. J.; Crosa, J. H. Vibrio Ordalii Sp. Nov.: A Causative Agent of Vibriosis in Fish. Curr. Microbiol. 1981, 6 (6), 343–348. https://doi.org/10.1007/BF01567009.
128. Liu, L.; Xiao, J.; Zhang, M.; Zhu, W.; Xia, X.; Dai, X.; Pan, Y.; Yan, S.; Wang, Y. A Vibrio Owensii Strain as the Causative Agent of AHPND in Cultured Shrimp, Litopenaeus Vannamei. J. Invertebr. Pathol. 2018, 153 (October 2017), 156–164. https://doi.org/10.1016/j.jip.2018.02.005.
129. Ishimaru, K.; Akagawa-Matsushita, M.; Muroga, K. Vibrio Penaeicida Sp. Nov., a Pathogen of Kuruma Prawns (Penaeus Japonicus). Int. J. Syst. Bacteriol. 1995, 45 (1), 134–138. https://doi.org/10.1099/00207713-45-1-134.
130. Rojas, R.; Miranda, C. D.; Opazo, R.; Romero, J. Characterization and Pathogenicity of Vibrio Splendidus Strains Associated with Massive Mortalities of Commercial Hatchery-Reared Larvae of Scallop Argopecten Purpuratus (Lamarck, 1819). J. In-vertebr. Pathol. 2015, 124, 61–69. https://doi.org/10.1016/j.jip.2014.10.009.
131. Allam, B.; Paillard, C.; Ford, S. Pathogenicity of Vibrio Tapetis, the Etiological Agent of Brown Ring Disease in Clams. Dis. Aquat. Organ. 2002, 48 (3), 221–231. https://doi.org/10.3354/dao048221.
132. Islam, S. S.; Zhang, S.; Eggermont, M.; Bruto, M.; Le Roux, F.; Defoirdt, T. The Impact of the Multichannel Quorum Sensing Systems of Vibrio Tasmaniensis and Vibrio Crassostreae on Virulence towards Blue Mussel (Mytilus Edulis) Larvae. Aqua-culture 2022, 547 (August 2021), 737414. https://doi.org/10.1016/j.aquaculture.2021.737414.
133. Pajdak‐Czaus, J.; Platt‐Samoraj, A.; Szweda, W.; Siwicki, A. K.; Terech‐Majewska, E. Yersinia Ruckeri —A Threat Not Only to Rainbow Trout. Aquac. Res. 2019, 50 (11), 3083–3096. https://doi.org/10.1111/are.14274.
134. Ramia, N. E.; El Kheir, S. M.; Taha, S.; Mangavel, C.; Revol-Junelles, A. M.; Borges, F. Multilocus Sequence Typing of Carno-bacterium Maltaromaticum Strains Associated with Fish Disease and Dairy Products. J. Appl. Microbiol. 2019, 126 (2), 377–387. https://doi.org/10.1111/jam.14127.
135. Preena, P. G.; Arathi, D.; Raj, N. S.; Arun Kumar, T. V.; Arun Raja, S.; Reshma, R. N.; Raja Swaminathan, T. Diversity of Antimicrobial‐resistant Pathogens from a Freshwater Ornamental Fish Farm. Lett. Appl. Microbiol. 2020, 71 (1), 108–116. https://doi.org/10.1111/lam.13231.
136. Harikrishnan, R.; Balasundaram, C.; Heo, M.-S. Scuticociliatosis and Its Recent Prophylactic Measures in Aquaculture with Special Reference to South Korea. Fish Shellfish Immunol. 2010, 29 (1), 15–31. https://doi.org/10.1016/j.fsi.2010.02.026.
137. Ugalde, S. C.; Preston, J.; Ogier, E.; Crawford, C. Analysis of Farm Management Strategies Following Herpesvirus (OsHV-1) Disease Outbreaks in Pacific Oysters in Tasmania, Australia. Aquaculture 2018, 495 (March), 179–186. https://doi.org/10.1016/j.aquaculture.2018.05.019.
138. Xue, M.; Jiang, N.; Fan, Y.; Yang, T.; Li, M.; Liu, W.; Li, Y.; Li, B.; Zeng, L.; Zhou, Y. White Spot Syndrome Virus (WSSV) Infection Alters Gut Histopathology and Microbiota Composition in Crayfish (Procambarus Clarkii). Aquac. Reports 2022, 22. https://doi.org/10.1016/j.aqrep.2022.101006.
139. Chan, G. F.; Puad, M. S. A.; Chin, C. F.; Rashid, N. A. A. Emergence of Aureobasidium Pullulans as Human Fungal Pathogen and Molecular Assay for Future Medical Diagnosis. Folia Microbiol. (Praha). 2011, 56 (5), 459–467. https://doi.org/10.1007/s12223-011-0070-9.
140. Sharma, C.; Kadosh, D. Perspective on the Origin, Resistance, and Spread of the Emerging Human Fungal Pathogen Candida Auris. PLOS Pathog. 2023, 19 (3), e1011190. https://doi.org/10.1371/journal.ppat.1011190.
141. Rojas, O. C.; Montoya, A. M.; Treviño-Rangel, R. de J. Clavispora Lusitaniae: From a Saprophytic Yeast to an Emergent Pathogen. Fungal Biol. 2024, 128 (5), 1933–1938. https://doi.org/10.1016/j.funbio.2024.05.013.
142. Sabiiti, W.; May, R. C. Mechanisms of Infection by the Human Fungal Pathogen Cryptococcus Neoformans. Future Microbiol. 2012, 7 (11), 1297–1313. https://doi.org/10.2217/fmb.12.102.
143. Pottier, I.; Gente, S.; Vernoux, J.; Gueguen, M. Safety Assessment of Dairy Microorganisms: Geotrichum Candidum☆. Int. J. Food Microbiol. 2008, 126 (3), 327–332. https://doi.org/10.1016/j.ijfoodmicro.2007.08.021.
144. Rodríguez‐Cerdeira, C.; Pinto‐Almazán, R.; Saunte, D. M. L.; Hay, R.; Szepietowski, J. C.; Moreno‐Coutiño, G.; Skerlev, M.; Prohic, A.; Martínez‐Herrera, E. Virulence and Resistance Factors of Nakaseomyces Glabratus (Formerly Known as Candida Glabrata) in Europe: A Systematic Review. J. Eur. Acad. Dermatology Venereol. 2024, No. July 2023, 1–12. https://doi.org/10.1111/jdv.20273.
145. Guitard, J.; Angoulvant, A.; Letscher-Bru, V.; L'Ollivier, C.; Cornet, M.; Dalle, F.; Grenouillet, F.; Lacroix, C.; Vekhoff, A.; Maury, E.; Caillot, D.; Charles, P. E.; Pili-Floury, S.; Herbrecht, R.; Raffoux, E.; Brethon, B.; Hennequin, C. Invasive Infections Due to Candida Norvegensis and Candida Inconspicua : Report of 12 Cases and Review of the Literature. Med. Mycol. 2013, 51 (8), 795–799. https://doi.org/10.3109/13693786.2013.807444.
146. Nguyen, T. A.; Kim, H. Y.; Stocker, S.; Kidd, S.; Alastruey-Izquierdo, A.; Dao, A.; Harrison, T.; Wahyuningsih, R.; Rickerts, V.; Perfect, J.; Denning, D. W.; Nucci, M.; Cassini, A.; Beardsley, J.; Gigante, V.; Sati, H.; Morrissey, C. O.; Alffenaar, J.-W. Pichia Kudriavzevii (Candida Krusei): A Systematic Review to Inform the World Health Organisation Priority List of Fungal Path-ogens. Med. Mycol. 2024, 62 (6). https://doi.org/10.1093/mmy/myad132.
147. de Almeida Júnior, J. N.; Ibrahim, K. Y.; Del Negro, G. M. B.; Bezerra, E. D.; Duarte Neto, A. N.; Batista, M. V.; Siciliano, R. F.; Giudice, M. C.; Motta, A. L.; Rossi, F.; Pierrotti, L. C.; Freire, M. P.; Bellesso, M.; Pereira, J.; Abdala, E.; Benard, G. Rhizopus Arrhizus and Fusarium Solani Concomitant Infection in an Immunocompromised Host. Mycopathologia 2016, 181 (1–2), 125–129. https://doi.org/10.1007/s11046-015-9936-8.
148. Ioannou, P.; Vamvoukaki, R.; Samonis, G. Rhodotorula Species Infections in Humans: A Systematic Review. Mycoses 2019, 62 (2), 90–100. https://doi.org/10.1111/myc.12856.
149. Pérez-Torrado, R.; Querol, A. Opportunistic Strains of Saccharomyces Cerevisiae: A Potential Risk Sold in Food Products. Front. Microbiol. 2016, 6 (JAN), 1–5. https://doi.org/10.3389/fmicb.2015.01522.
150. Hatvani, L.; Manczinger, L.; Vágvölgyi, C.; Kredics, L. Trichoderma as a Human Pathogen. In Trichoderma: biology and applications; CABI Wallingford UK, 2013; pp 292–313.

151. Gnat, S.; Łagowski, D.; Nowakiewicz, A.; Osińska, M.; Kopiński, Ł. Population Differentiation, Antifungal Susceptibility, and Host Range of Trichophyton Mentagrophytes Isolates Causing Recalcitrant Infections in Humans and Animals. Eur. J. Clin. Microbiol. Infect. Dis. 2020, 39 (11), 2099–2113. https://doi.org/10.1007/s10096-020-03952-2.
152. Blechert, O.; Xiong, S.; Chen, J.; Brand, A. C.; Zhan, P. Nutritional Requirements of the Human Pathogenic Fungus, Tri-chophyton Rubrum, and Nutritional Immunity of the Human Skin as Barrier against Colonization. Fungal Biol. Rev. 2023, 45, 100330. https://doi.org/10.1016/j.fbr.2023.100330.
153. Clementi, C. F.; Murphy, T. F. Non-Typeable Haemophilus Influenzae Invasion and Persistence in the Human Respiratory Tract. Front. Cell. Infect. Microbiol. 2011, 1 (November), 1. https://doi.org/10.3389/fcimb.2011.00001.
154. Quillin, S. J.; Seifert, H. S. Neisseria Gonorrhoeae Host Adaptation and Pathogenesis. Nat. Rev. Microbiol. 2018, 16 (4), 226–240. https://doi.org/10.1038/nrmicro.2017.169.
155. Guidone, G. H. M.; Cardozo, J. G.; Silva, L. C.; Sanches, M. S.; Galhardi, L. C. F.; Kobayashi, R. K. T.; Vespero, E. C.; Rocha, S. P. D. Epidemiology and Characterization of Providencia Stuartii Isolated from Hospitalized Patients in Southern Brazil: A Possible Emerging Pathogen. Access Microbiol. 2023, 5 (10), 1–18. https://doi.org/10.1099/acmi.0.000652.v4.
156. Patrick, M. E.; Adcock, P. M.; Gomez, T. M.; Altekruse, S. F.; Holland, B. H.; Tauxe, R. V.; Swerdlow, D. L. Salmonella Enteritidis Infections, United States, 1985–1999. Emerg. Infect. Dis. 2004, 10 (1), 1–7. https://doi.org/10.3201/eid1001.020572.
157. Crim, S. M.; Chai, S. J.; Karp, B. E.; Judd, M. C.; Reynolds, J.; Swanson, K. C.; Nisler, A.; McCullough, A.; Gould, L. H. Sal-monella Enterica Serotype Newport Infections in the United States, 2004–2013: Increased Incidence Investigated Through Four Surveillance Systems. Foodborne Pathog. Dis. 2018, 15 (10), 612–620. https://doi.org/10.1089/fpd.2018.2450.
158. Shad, A. A.; Shad, W. A. Shigella Sonnei: Virulence and Antibiotic Resistance. Arch. Microbiol. 2021, 203 (1), 45–58. https://doi.org/10.1007/s00203-020-02034-3.
159. Mages, I. S.; Frodl, R.; Bernard, K. A.; Funke, G. Identities of Arthrobacter Spp. and Arthrobacter -Like Bacteria Encountered in Human Clinical Specimens. J. Clin. Microbiol. 2008, 46 (9), 2980–2986. https://doi.org/10.1128/JCM.00658-08.
160. Haydushka, I.; Markova, N.; Kirina, V.; Atanassova, M. Recurrent Sepsis Due to Bacillus Licheniformis. J. Glob. Infect. Dis. 2012, 4 (1), 82. https://doi.org/10.4103/0974-777X.93768.
161. Bocchi, M. B.; Perna, A.; Cianni, L.; Vitiello, R.; Greco, T.; Maccauro, G.; Perisano, C. A Rare Case of Bacillus Megaterium Soft Tissues Infection. Acta Bio Medica Atenei Parm. 2020, 91, 1–5. https://doi.org/10.23750/abm.v91i14-S.10849.
162. Helgason, E.; Caugant, D. A.; Olsen, I.; Kolstø, A.-B. Genetic Structure of Population of Bacillus Cereus and B. Thuringiensis Isolates Associated with Periodontitis and Other Human Infections. J. Clin. Microbiol. 2000, 38 (4), 1615–1622. https://doi.org/10.1128/JCM.38.4.1615-1622.2000.
163. Gabr, E.; Awadalla, M.; Yasin, M. Antibacterial Activity of Some Essential Plant Oils Against Clinical Strain of Corynebacterium Stationis. Egypt. Acad. J. Biol. Sci. G. Microbiol. 2022, 14 (2), 205–213. https://doi.org/10.21608/eajbsg.2022.278873.
164. Eishi, Y. Potential Association of Cutibacterium Acnes with Sarcoidosis as an Endogenous Hypersensitivity Infection. Micro-organisms 2023, 11 (2). https://doi.org/10.3390/microorganisms11020289.
165. Joron, C.; Roméo, B.; Le Flèche-Matéos, A.; Rames, C.; El Samad, Y.; Hamdad, F. Dermacoccus Nishinomiyaensis as a Cause of Persistent Paediatric Catheter-Related Bacteraemia. Clin. Microbiol. Infect. 2019, 25 (8), 1054–1055. https://doi.org/10.1016/j.cmi.2019.02.023.
166. Keynan, Y.; Weber, G.; Sprecher, H. Molecular Identification of Exiguobacterium Acetylicum as the Aetiological Agent of Bacteraemia. J. Med. Microbiol. 2007, 56 (4), 563–564. https://doi.org/10.1099/jmm.0.46866-0.
167. Rossi, F.; Giaccone, V.; Colavita, G.; Amadoro, C.; Pomilio, F.; Catellani, P. Virulence Characteristics and Distribution of the Pathogen Listeria Ivanovii in the Environment and in Food. Microorganisms 2022, 10 (8), 1–19. https://doi.org/10.3390/microorganisms10081679.
168. Drevets, D. A.; Bronze, M. S. Listeria Monocytogenes: Epidemiology, Human Disease, and Mechanisms of Brain Invasion. FEMS Immunol. Med. Microbiol. 2008, 53 (2), 151–165. https://doi.org/10.1111/j.1574-695X.2008.00404.x.
169. Yeung, E. Y. H.; Chowdhury, M. F.; Slinger, R. W. First Case Report of Infective Endocarditis Associated with Microbacterium Maritypicum. IDCases 2020, 22, e00952. https://doi.org/10.1016/j.idcr.2020.e00952.
170. Leach, J. E.; Medinger, F. G. Micrococcus Tetragenus Meningitis; Report of a Case and Review of the Literature. Ann. Intern. Med. 1941, 15 (3), 609–616. https://doi.org/10.7326/0003-4819-15-3-609.
171. Tanaka, S.; Hoshino, Y.; Sakagami, T.; Fukano, H.; Matsui, Y.; Hiranuma, O. Pathogenicity of Mycolicibacterium Phlei, a Non-Pathogenic Nontuberculous Mycobacterium in an Immunocompetent Host Carrying Anti-Interferon Gamma Autoanti-bodies: A Case Report. BMC Infect. Dis. 2019, 19 (1), 4–9. https://doi.org/10.1186/s12879-019-4050-z.
172. Daboor, S. M.; Syed, F.; Masood, S.; Al-Azab, M. S.; Nori, E. A Review on Streptococcus Mutans with Its Diseases Dental Caries, Dental Plaque and Endocarditis. Teach. Assist. Microbiol. 2015, 2 (2), 4.
173. Bessen, D. E. Population Biology of the Human Restricted Pathogen, Streptococcus Pyogenes. Infect. Genet. Evol. 2009, 9 (4), 581–593. https://doi.org/10.1016/j.meegid.2009.03.002.
174. Simsek, A. D.; Sezer, S.; Ozdemir, N. F.; Mehmet, H. Streptococcus Vestibularis Bacteremia Following Dental Extraction in a Patient on Long-Term Hemodialysis: A Case Report. Clin. Kidney J. 2008, 1 (4), 276–277. https://doi.org/10.1093/ndtplus/sfn071.
175. Wright, M. H.; Paape, D.; Storck, E. M.; Serwa, R. A.; Smith, D. F.; Tate, E. W. Global Analysis of Protein N-Myristoylation and Exploration of N-Myristoyltransferase as a Drug Target in the Neglected Human Pathogen Leishmania Donovani. Chem. Biol. 2015, 22 (3), 342–354. https://doi.org/10.1016/j.chembiol.2015.01.003.
176. Marsland, B. J.; Kurrer, M.; Reissmann, R.; Harris, N. L.; Kopf, M. Nippostrongylus Brasiliensis Infection Leads to the Devel-opment of Emphysema Associated with the Induction of Alternatively Activated Macrophages. 2008, 479–488. https://doi.org/10.1002/eji.200737827.
177. Kissinger, P. Trichomonas Vaginalis: A Review of Epidemiologic, Clinical and Treatment Issues. BMC Infect. Dis. 2015, 15 (1), 1–8. https://doi.org/10.1186/s12879-015-1055-0.
178. Machado, F. S.; Dutra, W. O.; Esper, L.; Gollob, K. J.; Teixeira, M. M.; Factor, S. M.; Weiss, L. M.; Nagajyothi, F.; Tanowitz, H. B.; Garg, N. J. Current Understanding of Immunity to Trypanosoma Cruzi Infection and Pathogenesis of Chagas Disease. Semin. Immunopathol. 2012, 34 (6), 753–770. https://doi.org/10.1007/s00281-012-0351-7.
179. Hollingsworth, T. D.; Anderson, R. M.; Fraser, C. HIV-1 Transmission, by Stage of Infection. J. Infect. Dis. 2008, 198 (5), 687–693. https://doi.org/10.1086/590501.
180. Rice, S. A. Release of HSV-1 Cell-Free Virions: Mechanisms, Regulation, and Likely Role in Human-Human Transmission. Viruses 2021, 13 (12), 2395. https://doi.org/10.3390/v13122395.
181. Johnston, C.; Koelle, D. M.; Wald, A. HSV-2: In Pursuit of a Vaccine. J. Clin. Invest. 2011, 121 (12), 4600–4609. https://doi.org/10.1172/JCI57148.
182. He, J.; Tao, H.; Yan, Y.; Huang, S. Y.; Xiao, Y. Molecular Mechanism of Evolution and Human Infection with SARS-CoV-2. Viruses 2020, 12 (4). https://doi.org/10.3390/v12040428.
183. Aziz, H.; Zia, A.; Anwer, A.; Aziz, M.; Fatima, S.; Faheem, M. Zika Virus: Global Health Challenge, Threat and Current Situation. J. Med. Virol. 2017, 89 (6), 943–951. https://doi.org/10.1002/jmv.24731.
184. Leong, C.; Chan, J. W. K.; Lee, S. M.; Lam, Y. I.; Goh, J. P. Z.; Ianiri, G.; Dawson, T. L. Azole Resistance Mechanisms in Pathogenic Malassezia Furfur. Antimicrob. Agents Chemother. 2021, 65 (5). https://doi.org/10.1128/AAC.01975-20.
185. Schmidt, A. Malassezia Furfur: A Fungus Belonging to the Physiological Skin Flora and Its Relevance in Skin Disorders. Cutis 1997, 59 (1), 21—24

186. Schikorski, D.; Cuvillier-Hot, V.; Leippe, M.; Boidin-Wichlacz, C.; Slomianny, C.; Macagno, E.; Salzet, M.; Tasiemski, A. Mi-crobial Challenge Promotes the Regenerative Process of the Injured Central Nervous System of the Medicinal Leech by Inducing the Synthesis of Antimicrobial Peptides in Neurons and Microglia. J. Immunol. 2008, 181 (2), 1083–1095. https://doi.org/10.4049/jimmunol.181.2.1083.
187. Ding, A.; Shi, H.; Guo, Q.; Liu, F.; Wang, J.; Cheng, B.; Wei, W.; Xu, C. Gene Cloning and Expression of a Partial Sequence of Hirudomacin, an Antimicrobial Protein That Is Increased in Leech (Hirudo Nipponica Whitman) after a Blood Meal. Comp. Biochem. Physiol. Part B Biochem. Mol. Biol. 2019, 231 (1), 75–86. https://doi.org/10.1016/j.cbpb.2019.02.005.
188. Jung, S.; Sönnichsen, F. D.; Hung, C.-W.; Tholey, A.; Boidin-Wichlacz, C.; Haeusgen, W.; Gelhaus, C.; Desel, C.; Podschun, R.; Waetzig, V.; Tasiemski, A.; Leippe, M.; Grötzinger, J. Macin Family of Antimicrobial Proteins Combines Antimicrobial and Nerve Repair Activities. J. Biol. Chem. 2012, 287 (17), 14246–14258. https://doi.org/10.1074/jbc.M111.336495.
189. Tasiemski, A.; Vandenbulcke, F.; Mitta, G.; Lemoine, J.; Lefebvre, C.; Sautière, P. E.; Salzet, M. Molecular Characterization of Two Novel Antibacterial Peptides Inducible upon Bacterial Challenge in an Annelid, the Leech Theromyzon Tessulatum. J. Biol. Chem. 2004, 279 (30), 30973–30982. https://doi.org/10.1074/jbc.M312156200.
190. Elliott, A. G.; Huang, J. X.; Neve, S.; Zuegg, J.; Edwards, I. A.; Cain, A. K.; Boinett, C. J.; Barquist, L.; Lundberg, C. V.; Steen, J.; Butler, M. S.; Mobli, M.; Porter, K. M.; Blaskovich, M. A. T.; Lociuro, S.; Strandh, M.; Cooper, M. A. An Amphipathic Peptide with Antibiotic Activity against Multidrug-Resistant Gram-Negative Bacteria. Nat. Commun. 2020, 11 (1). https://doi.org/10.1038/s41467-020-16950-x.
191. Safronova, V. N.; Bolosov, I. A.; Kruglikov, R. N.; Korobova, O. V.; Pereskokova, E. S.; Borzilov, A. I.; Panteleev, P. V.; Ovchinnikova, T. V. Novel β-Hairpin Peptide from Marine Polychaeta with a High Efficacy against Gram-Negative Pathogens. Mar. Drugs 2022, 20 (8), 2–5. https://doi.org/10.3390/md20080517.
192. Tasiemski, A.; Jung, S.; Boidin-Wichlacz, C.; Jollivet, D.; Cuvillier-Hot, V.; Pradillon, F.; Vetriani, C.; Hecht, O.; Sönnichsen, F. D.; Gelhaus, C.; Hung, C.-W.; Tholey, A.; Leippe, M.; Grötzinger, J.; Gaill, F. Characterization and Function of the First Anti-biotic Isolated from a Vent Organism: The Extremophile Metazoan Alvinella Pompejana. PLoS One 2014, 9 (4), e95737. https://doi.org/10.1371/journal.pone.0095737.
193. Bruno, R.; Boidin-Wichlacz, C.; Melnyk, O.; Zeppilli, D.; Landon, C.; Thomas, F.; Cambon, M. A.; Lafond, M.; Mabrouk, K.; Massol, F.; Hourdez, S.; Maresca, M.; Jollivet, D.; Tasiemski, A. The Diversification of the Antimicrobial Peptides from Marine Worms Is Driven by Environmental Conditions. Sci. Total Environ. 2023, 879, 1–5. https://doi.org/10.1016/j.scitotenv.2023.162875.
194. Safronova, V. N.; Panteleev, P. V.; Kruglikov, R. N.; Bolosov, I. A.; Finkina, E. I.; Ovchinnikova, T. V. Novel BRICHOS-Related Defensin-like Antimicrobial Peptide from the Marine Polychaeta Arenicola Marina. Russ. J. Bioorganic Chem. 2024, 50 (3), 629–643. https://doi.org/10.1134/S1068162024030087.
195. Ovchinnikova, T. V.; Aleshina, G. M.; Balandin, S. V.; Krasnosdembskaya, A. D.; Markelov, M. L.; Frolova, E. I.; Leonova, Y. F.; Tagaev, A. A.; Krasnodembsky, E. G.; Kokryakov, V. N. Purification and Primary Structure of Two Isoforms of Arenicin, a Novel Antimicrobial Peptide from Marine Polychaeta Arenicola Marina. FEBS Lett. 2004, 577 (1–2), 209–214. https://doi.org/10.1016/j.febslet.2004.10.012.
196. Lee, J.-U.; Kang, D.-I.; Zhu, W. L.; Shin, S. Y.; Hahm, K.-S.; Kim, Y. Solution Structures and Biological Functions of the Anti-microbial Peptide, Arenicin-1, and Its Linear Derivative. Biopolymers 2007, 88 (2), 208–216. https://doi.org/10.1002/bip.20700.
197. Edwards, I. A.; Elliott, A. G.; Kavanagh, A. M.; Zuegg, J.; Blaskovich, M. A. T.; Cooper, M. A. Contribution of Amphipathicity and Hydrophobicity to the Antimicrobial Activity and Cytotoxicity of β-Hairpin Peptides. ACS Infect. Dis. 2016, 2 (6), 442–450. https://doi.org/10.1021/acsinfecdis.6b00045.
198. Panteleev, P. V.; Safronova, V. N.; Duan, S.; Komlev, A. S.; Bolosov, I. A.; Kruglikov, R. N.; Kombarova, T. I.; Korobova, O. V.; Pereskokova, E. S.; Borzilov, A. I.; Dyachenko, I. A.; Shamova, O. V.; Huang, Y.; Shi, Q.; Ovchinnikova, T. V. Novel BRICHOS-Related Antimicrobial Peptides from the Marine Worm Heteromastus Filiformis: Transcriptome Mining, Synthesis, Biological Activities, and Therapeutic Potential. Mar. Drugs 2023, 21 (12), 639. https://doi.org/10.3390/md21120639.
199. Yang, D.; Wang, Q.; Chen, L.; Liu, Y.; Cao, R.; Wu, H.; Li, F.; Ji, C.; Cong, M.; Zhao, J. Molecular Characterization and Anti-bacterial Activity of a Phage-Type Lysozyme from the Manila Clam, Ruditapes Philippinarum. Fish Shellfish Immunol. 2017, 65, 17–24. https://doi.org/10.1016/j.fsi.2017.03.051.
200. Panteleev, P. V.; Tsarev, A. V.; Bolosov, I. A.; Paramonov, A. S.; Marggraf, M. B.; Sychev, S. V.; Shenkarev, Z. O.; Ovchinnikova, T. V. Novel Antimicrobial Peptides from the Arctic Polychaeta Nicomache Minor Provide New Molecular Insight into Biological Role of the BRICHOS Domain. Mar. Drugs 2018, 16 (11), 401. https://doi.org/10.3390/md16110401.
201. Tasiemski, A.; Schikorski, D.; Le Marrec-Croq, F.; Pontoire-Van Camp, C.; Boidin-Wichlacz, C.; Sautière, P.-E. Hedistin: A Novel Antimicrobial Peptide Containing Bromotryptophan Constitutively Expressed in the NK Cells-like of the Marine Annelid, Nereis Diversicolor. Dev. Comp. Immunol. 2007, 31 (8), 749–762. https://doi.org/10.1016/j.dci.2006.11.003.
202. Joo, M.-S.; Choi, K.-M.; Cho, D.-H.; Choi, H.-S.; Min, E. Y.; Han, H.-J.; Cho, M. Y.; Bae, J.-S.; Park, C.-I. The Molecular Char-acterization, Expression Analysis and Antimicrobial Activity of Theromacin from Asian Polychaeta (Perinereis Linea). Dev. Comp. Immunol. 2020, 112 (May), 103773. https://doi.org/10.1016/j.dci.2020.103773.
203. Pan, W.; Liu, X.; Ge, F.; Han, J.; Zheng, T. Perinerin, a Novel Antimicrobial Peptide Purified from the Clamworm Perinereis Aibuhitensis Grube and Its Partial Characterization. J. Biochem. 2004, 135 (3), 297–304. https://doi.org/10.1093/jb/mvh036.
204. Imjongjirak, C.; Amparyup, P.; Tassanakajon, A.; Sittipraneed, S. Antilipopolysaccharide Factor (ALF) of Mud Crab Scylla Paramamosain: Molecular Cloning, Genomic Organization and the Antimicrobial Activity of Its Synthetic LPS Binding Domain. Mol. Immunol. 2007, 44 (12), 3195–3203. https://doi.org/10.1016/j.molimm.2007.01.028.
205. Imjongjirak, C.; Amparyup, P.; Tassanakajon, A. Molecular Cloning, Genomic Organization and Antibacterial Activity of a Second Isoform of Antilipopolysaccharide Factor (ALF) from the Mud Crab, Scylla Paramamosain. Fish Shellfish Immunol. 2011, 30 (1), 58–66. https://doi.org/10.1016/j.fsi.2010.09.011.
206. Morita, T.; Ohtsubo, S.; Nakamura, T.; Tanaka, S.; Iwanaga, S.; Ohashi, K.; Niwa, M. Isolation and Biological Activities of Limulus Anticoagulant (Anti-LPS Factor) Which Interacts with Lipopolysaccharide (LPS). J. Biochem. 1985, 97 (6), 1611–1620. https://doi.org/10.1093/oxfordjournals.jbchem.a135218.
207. Liu, Y.; Cui, Z.; Li, X.; Song, C.; Li, Q.; Wang, S. A New Anti-Lipopolysaccharide Factor Isoform (PtALF4) from the Swimming Crab Portunus Trituberculatus Exhibited Structural and Functional Diversity of ALFs. Fish Shellfish Immunol. 2012, 32 (5), 724–731. https://doi.org/10.1016/j.fsi.2012.01.021.
208. Liu, Y.; Cui, Z.; Li, X.; Song, C.; Li, Q.; Wang, S. Molecular Cloning, Expression Pattern and Antimicrobial Activity of a New Isoform of Anti-Lipopolysaccharide Factor from the Swimming Crab Portunus Trituberculatus. Fish Shellfish Immunol. 2012, 33 (1), 85–91. https://doi.org/10.1016/j.fsi.2012.04.004.
209. Liu, Y.; Cui, Z.; Li, X.; Song, C.; Shi, G. A Newly Identified Anti-Lipopolysaccharide Factor from the Swimming Crab Portunus Trituberculatus with Broad Spectrum Antimicrobial Activity. Fish Shellfish Immunol. 2013, 34 (2), 463–470. https://doi.org/10.1016/j.fsi.2012.11.050.
210. Liu, Y.; Cui, Z.; Li, X.; Song, C.; Shi, G.; Wang, C. Molecular Cloning, Genomic Structure and Antimicrobial Activity of PtALF7, a Unique Isoform of Anti-Lipopolysaccharide Factor from the Swimming Crab Portunus Trituberculatus. Fish Shellfish Im-munol. 2013, 34 (2), 652–659. https://doi.org/10.1016/j.fsi.2012.12.002.
211. Liu, H.; Chen, R.; Zhang, Q.; Wang, Q.; Li, C.; Peng, H.; Cai, L.; Zheng, C.; Wang, K. Characterization of Two Isoforms of Antiliopolysacchride Factors (Sp-ALFs) from the Mud Crab Scylla Paramamosain. Fish Shellfish Immunol. 2012, 33 (1), 1–10. https://doi.org/10.1016/j.fsi.2012.03.014.

212. Yedery, R. D.; Reddy, K. V. R. Identification, Cloning, Characterization and Recombinant Expression of an An-ti-Lipopolysaccharide Factor from the Hemocytes of Indian Mud Crab, Scylla Serrata. Fish Shellfish Immunol. 2009, 27 (2), 275–284. https://doi.org/10.1016/j.fsi.2009.05.009.
213. Saito, T.; Kawabata, S.; Shigenaga, T.; Takayenoki, Y.; Cho, J.; Nakajima, H.; Hirata, M.; Iwanaga, S. A Novel Big Defensin Identified in Horseshoe Crab Hemocytes: Isolation, Amino Acid Sequence, and Antibacterial Activity1. J. Biochem. 1995, 117 (5), 1131–1137. https://doi.org/10.1093/oxfordjournals.jbchem.a124818.
214. Osaki, T.; Omotezako, M.; Nagayama, R.; Hirata, M.; Iwanaga, S.; Kasahara, J.; Hattori, J.; Ito, I.; Sugiyama, H.; Kawabata, S. Horseshoe Crab Hemocyte-Derived Antimicrobial Polypeptides, Tachystatins, with Sequence Similarity to Spider Neurotoxins. J. Biol. Chem. 1999, 274 (37), 26172–26178. https://doi.org/10.1074/jbc.274.37.26172.
215. Miyata, T.; Tokunaga, F.; Yoneya, T.; Yoshikawa, K.; Iwanaga, S.; Niwa, M.; Takao, T.; Shimonishi, Y. Antimicrobial Peptides, Isolated from Horseshoe Crab Hemocytes, Tachyplesin II, and Polyphemusins I and II: Chemical Structures and Biological Activity1. J. Biochem. 1989, 106 (4), 663–668. https://doi.org/10.1093/oxfordjournals.jbchem.a122913.
216. Zhang, L.; Scott, M. G.; Yan, H.; Mayer, L. D.; Hancock, R. E. W. Interaction of Polyphemusin I and Structural Analogs with Bacterial Membranes, Lipopolysaccharide, and Lipid Monolayers. Biochemistry 2000, 39 (47), 14504–14514. https://doi.org/10.1021/bi0011173.
217. Marggraf, M. B.; Panteleev, P. V.; Emelianova, A. A.; Sorokin, M. I.; Bolosov, I. A.; Buzdin, A. A.; Kuzmin, D. V.; Ovchinnikova, T. V. Cytotoxic Potential of the Novel Horseshoe Crab Peptide Polyphemusin III. Mar. Drugs 2018, 16 (12), 466. https://doi.org/10.3390/md16120466.
218. Kawabata, S.; Nagayama, R.; Hirata, M.; Shigenaga, T.; Agarwala, K. L.; Saito, T.; Cho, J.; Nakajima, H.; Takagi, T.; Iwanaga, S. Tachycitin, a Small Granular Component in Horseshoe Crab Hemocytes, Is an Antimicrobial Protein with Chitin-Binding Activity. J. Biochem. 1996, 120 (6), 1253–1260. https://doi.org/10.1093/oxfordjournals.jbchem.a021549.
219. Nakamura, T.; Furunaka, H.; Miyata, T.; Tokunaga, F.; Muta, T.; Iwanaga, S.; Niwa, M.; Takao, T.; Shimonishi, Y. Tachyplesin, a Class of Antimicrobial Peptide from the Hemocytes of the Horseshoe Crab (Tachypleus Tridentatus). Isolation and Chemical Structure. J. Biol. Chem. 1988, 263 (32), 16709–16713. https://doi.org/10.1016/s0021-9258(18)37448-9.
220. Löfgren, S. E.; Miletti, L. C.; Steindel, M.; Bachère, E.; Barracco, M. A. Trypanocidal and Leishmanicidal Activities of Different Antimicrobial Peptides (AMPs) Isolated from Aquatic Animals. Exp. Parasitol. 2008, 118 (2), 197–202. https://doi.org/10.1016/j.exppara.2007.07.011.
221. Kumar, V.; Chugh, A. Peptide-Mediated Leishmaniasis Management Strategy: Tachyplesin Emerges as an Effective An-ti-Leishmanial Peptide against Leishmania Donovani. Biochim. Biophys. Acta - Biomembr. 2021, 1863 (8), 183629. https://doi.org/10.1016/j.bbamem.2021.183629.
222. Kasturi, M. M. M.; Kamaruding, N. A.; Ismail, N. Characterization of Purified Tachystatin-A2 Isolated from Amoebocytes of Asian Horseshoe Crab, Tachypleus Gigas as Potential Antibacterial Peptide. Appl. Biochem. Microbiol. 2021, 57 (3), 311–318. https://doi.org/10.1134/S0003683821030054.
223. Wang, W.-F.; Cheng, C.-X.; Liu, H.; Chen, X.-L.; Wang, H.-L. 6His-Tatritin Promotes Antimicrobial Defense via Regulating Immune Ability and Intestinal Microbial Community in Grass Carp (Ctenopharyngodon Idella). Fish Shellfish Immunol. 2023, 133 (January), 108532. https://doi.org/10.1016/j.fsi.2023.108532.
224. Wang, W.-F.; Xie, X.-Y.; Huang, Y.; Li, Y.-K.; Liu, H.; Chen, X.-L.; Wang, H.-L. Identification of a Novel Antimicrobial Peptide From the Ancient Marine Arthropod Chinese Horseshoe Crab, Tachypleus Tridentatus. Front. Immunol. 2022, 13 (March), 1–15. https://doi.org/10.3389/fimmu.2022.794779.
225. Li, S.; Guo, S.; Li, F.; Xiang, J. Functional Diversity of Anti-Lipopolysaccharide Factor Isoforms in Shrimp and Their Characters Related to Antiviral Activity. Mar. Drugs 2015, 13 (5), 2602–2616. https://doi.org/10.3390/md13052602.
226. Somboonwiwat, K.; Marcos, M.; Tassanakajon, A.; Klinbunga, S.; Aumelas, A.; Romestand, B.; Gueguen, Y.; Boze, H.; Moulin, G.; Bachère, E. Recombinant Expression and Anti-Microbial Activity of Anti- Lipopolysaccharide Factor (ALF) from the Black Tiger Shrimp Penaeus Monodon. Dev. Comp. Immunol. 2005, 29 (10), 841–851. https://doi.org/10.1016/j.dci.2005.02.004.
227. Suraprasit, S.; Methatham, T.; Jaree, P.; Phiwsaiya, K.; Senapin, S.; Hirono, I.; Lo, C. F.; Tassanakajon, A.; Somboonwiwat, K. Anti-Lipopolysaccharide Factor Isoform 3 from Penaeus Monodon (ALFPm3) Exhibits Antiviral Activity by Interacting with WSSV Structural Proteins. Antiviral Res. 2014, 110, 142–150. https://doi.org/10.1016/j.antiviral.2014.08.005.
228. Tharntada, S.; Ponprateep, S.; Somboonwiwat, K.; Liu, H.; Söderhäll, I.; Söderhäll, K.; Tassanakajon, A. Role of An-ti-Lipopolysaccharide Factor from the Black Tiger Shrimp, Penaeus Monodon, in Protection from White Spot Syndrome Virus Infection. J. Gen. Virol. 2009, 90 (6), 1491–1498. https://doi.org/10.1099/vir.0.009621-0.
229. Zhou, L.; Li, G.; Li, A.; Jiao, Y.; Li, S.; Huang, J.; Yang, L.; Wang, C. Characterization of a Group D Anti-Lipopolysaccharide Factor (ALF) Involved in Anti-Vibrio Response in Penaeus Monodon. Fish Shellfish Immunol. 2019, 89 (March), 384–392. https://doi.org/10.1016/j.fsi.2019.03.049.
230. Kamsaeng, P.; Tassanakajon, A.; Somboonwiwat, K. Regulation of Antilipopolysaccharide Factors, ALFPm3 and ALFPm6, in Penaeus Monodon. Sci. Rep. 2017, 7 (1), 12694. https://doi.org/10.1038/s41598-017-12137-5.
231. Sruthy, K. S.; Philip, R. Anti-Lipopolysaccharide Factor from Crucifix Crab Charybdis Feriatus, Cf-ALF2: Molecular Cloning and Functional Characterization of the Recombinant Peptide. Probiotics Antimicrob. Proteins 2021, 13 (3), 885–898. https://doi.org/10.1007/s12602-020-09716-w.
232. Pan, C. Y.; Chen, J. Y.; Lin, T. L.; Lin, C. H. In Vitro Activities of Three Synthetic Peptides Derived from Epinecidin-1 and an Anti-Lipopolysaccharide Factor against Propionibacterium Acnes, Candida Albicans, and Trichomonas Vaginalis. Peptides 2009, 30 (6), 1058–1068. https://doi.org/10.1016/j.peptides.2009.02.006.
233. Lv, X.; Li, S.; Liu, F.; Li, F.; Xiang, J. Identification and Function Analysis of an Anti-Lipopolysaccharide Factor from the Ridgetail Prawn Exopalaemon Carinicauda. Dev. Comp. Immunol. 2017, 70, 128–134. https://doi.org/10.1016/j.dci.2017.01.010.
234. Lv, X.; Li, S.; Zhang, C.; Xiang, J.; Li, F. Multiple Isoforms of Anti-Lipopolysaccharide Factors and Their Antimicrobial Functions in the Ridgetail Prawn Exopalaemon Carinicauda. Mar. Drugs 2018, 16 (5). https://doi.org/10.3390/md16050145.
235. Wang, L.; Zhang, Y.; Wang, L.; Yang, J.; Zhou, Z.; Gai, Y.; Qiu, L.; Song, L. A New Anti-Lipopolysaccharide Factor (EsALF-3) from Eriocheir Sinensis with Antimicrobial Activity. African J. Biotechnol. 2011, 10 (77), 17678–17689. https://doi.org/10.5897/AJB11.2017.
236. Li, S.; Guo, S.; Li, F.; Xiang, J. Characterization and Function Analysis of an Anti-Lipopolysaccharide Factor (ALF) from the Chinese Shrimp Fenneropenaeus Chinensis. Dev. Comp. Immunol. 2014, 46 (2), 349–355. https://doi.org/10.1016/j.dci.2014.05.013.
237. Li, S.; Lv, X.; Li, F.; Xiang, J. Characterization of a Lymphoid Organ Specific Anti-Lipopolysaccharide Factor from Shrimp Reveals Structure-Activity Relationship of the LPS-Binding Domain. Front. Immunol. 2019, 10 (APR). https://doi.org/10.3389/fimmu.2019.00872.
238. Rosa, R. D.; Vergnes, A.; de Lorgeril, J.; Goncalves, P.; Perazzolo, L. M.; Sauné, L.; Romestand, B.; Fievet, J.; Gueguen, Y.; Bachère, E.; Destoumieux-Garzón, D. Functional Divergence in Shrimp Anti-Lipopolysaccharide Factors (ALFs): From Recognition of Cell Wall Components to Antimicrobial Activity. PLoS One 2013, 8 (7), e67937. https://doi.org/10.1371/journal.pone.0067937.
239. Matos, G. M.; Schmitt, P.; Barreto, C.; Farias, N. D.; Toledo-Silva, G.; Guzmán, F.; Destoumieux-Garzón, D.; Perazzolo, L. M.; Rosa, R. D. Massive Gene Expansion and Sequence Diversification Is Associated with Diverse Tissue Distribution, Regulation and Antimicrobial Properties of Anti-Lipopolysaccharide Factors in Shrimp. Mar. Drugs 2018, 16 (10), 1–17. https://doi.org/10.3390/md16100381.
240. de la Vega, E.; O'Leary, N. A.; Shockey, J. E.; Robalino, J.; Payne, C.; Browdy, C. L.; Warr, G. W.; Gross, P. S. An-ti-Lipopolysaccharide Factor in Litopenaeus Vannamei (LvALF): A Broad Spectrum Antimicrobial Peptide Essential for Shrimp Immunity against Bacterial and Fungal Infection. Mol. Immunol. 2008, 45 (7), 1916–1925. https://doi.org/10.1016/j.molimm.2007.10.039.
241. Sun, M.; Li, S.; Lv, X.; Xiang, J.; Lu, Y.; Li, F. A Lymphoid Organ Specific Anti-Lipopolysaccharide Factor from Litopenaeus Vannamei Exhibits Strong Antimicrobial Activities. Mar. Drugs 2021, 19 (5). https://doi.org/10.3390/md19050250.
242. Jiang, H. S.; Lv, L. X.; Wang, J. X. Anti-Lipopolysaccharide Factor D from Kuruma Shrimp Exhibits Antiviral Activity. Mar. Life Sci. Technol. 2022, 4 (1), 52–61. https://doi.org/10.1007/s42995-021-00113-y.

243. Stensvåg, K.; Haug, T.; Sperstad, S. V; Rekdal, Ø.; Indrevoll, B.; Styrvold, O. B. Arasin 1, a Proline–Arginine-Rich Antimicrobial Peptide Isolated from the Spider Crab, Hyas Araneus. Dev. Comp. Immunol. 2008, 32 (3), 275–285. https://doi.org/10.1016/j.dci.2007.06.002.
244. Imjongjirak, C.; Amparyup, P.; Tassanakajon, A. Two Novel Antimicrobial Peptides, Arasin-LikeSp and GRPSp, from the Mud Crab Scylla Paramamosain, Exhibit the Activity against Some Crustacean Pathogenic Bacteria. Fish Shellfish Immunol. 2011, 30 (2), 706–712. https://doi.org/10.1016/j.fsi.2010.12.031.
245. Khoo, L.; Robinette, D. W.; Noga, E. J. Callinectin, an Antibacterial Peptide from Blue Crab, Callinectes Sapidus, Hemocytes. Mar. Biotechnol. 1999, 1 (1), 44–51. https://doi.org/10.1007/PL00011750.
246. Zhang, S.; Hou, C.; Xiao, B.; Yao, Y.; Xiao, W.; Li, C.; Shi, L. Identification and Function of an Arasin-like Peptide from Li-topenaeus Vannamei. Dev. Comp. Immunol. 2021, 125 (July), 104174. https://doi.org/10.1016/j.dci.2021.104174.
247. Chai, L.-Q. Q.; Li, W.-W. W.; Wang, X.-W. W. Identification and Characterization of Two Arasin-like Peptides in Red Swamp Crayfish Procambarus Clarkii. Fish Shellfish Immunol. 2017, 70, 673–681. https://doi.org/10.1016/j.fsi.2017.09.059.
248. Anju, A.; Smitha, C. K.; Preetha, K.; Boobal, R.; Rosamma, P. Molecular Characterization, Recombinant Expression and Bio-activity Profile of an Antimicrobial Peptide, Ss-Arasin from the Indian Mud Crab, Scylla Serrata. Fish Shellfish Immunol. 2019, 88 (November 2018), 352–358. https://doi.org/10.1016/j.fsi.2019.03.007.
249. Lee, S. Y.; Lee, B. L.; Söderhäll, K. Processing of an Antibacterial Peptide from Hemocyanin of the Freshwater Crayfish Pacifastacus Leniusculus. J. Biol. Chem. 2003, 278 (10), 7927–7933. https://doi.org/10.1074/jbc.M209239200.
250. Choi, H.; Lee, D. G. Antifungal Activity and Pore-Forming Mechanism of Astacidin 1 against Candida Albicans. Biochimie 2014, 105, 58–63. https://doi.org/10.1016/j.biochi.2014.06.014.
251. Jiravanichpaisal, P.; Lee, S. Y.; Kim, Y. A.; Andrén, T.; Söderhäll, I. Antibacterial Peptides in Hemocytes and Hematopoietic Tissue from Freshwater Crayfish Pacifastacus Leniusculus: Characterization and Expression Pattern. Dev. Comp. Immunol. 2007, 31 (5), 441–455. https://doi.org/10.1016/j.dci.2006.08.002.
252. Rončević, T.; Čikeš-Čulić, V.; Maravić, A.; Capanni, F.; Gerdol, M.; Pacor, S.; Tossi, A.; Giulianini, P. G.; Pallavicini, A.; Manfrin, C. Identification and Functional Characterization of the Astacidin Family of Proline-Rich Host Defence Peptides (PcAst) from the Red Swamp Crayfish (Procambarus Clarkii, Girard 1852). Dev. Comp. Immunol. 2020, 105 (December 2019), 103574. https://doi.org/10.1016/j.dci.2019.103574.
253. Tonganunt, M.; Wongmanee, K.; Sartthai, S.; Chotigeat, W.; Phongdara, A. Crustin Protein Amk1 from Black Tiger Shrimp (Penaeus Monodon) Inhibits Vibrio Harveyi and Staphylococcus Aureus. Songklanakarin J. Sci. Technol. 2008, 30 (3), 291–296.
254. Zhang, J.; Li, F.; Wang, Z.; Xiang, J. Cloning and Recombinant Expression of a Crustin-like Gene from Chinese Shrimp, Fen-neropenaeus Chinensis. J. Biotechnol. 2007, 127 (4), 605–614. https://doi.org/10.1016/j.jbiotec.2006.08.013.
255. Mu, C.; Zheng, P.; Zhao, J.; Wang, L.; Zhang, H.; Qiu, L.; Gai, Y.; Song, L. Molecular Characterization and Expression of a Crustin-like Gene from Chinese Mitten Crab, Eriocheir Sinensis. Dev. Comp. Immunol. 2010, 34 (7), 734–740. https://doi.org/10.1016/j.dci.2010.02.001.
256. Amparyup, P.; Kondo, H.; Hirono, I.; Aoki, T.; Tassanakajon, A. Molecular Cloning, Genomic Organization and Recombinant Expression of a Crustin-like Antimicrobial Peptide from Black Tiger Shrimp Penaeus Monodon. Mol. Immunol. 2008, 45 (4), 1085–1093. https://doi.org/10.1016/j.molimm.2007.07.031.
257. Antony, S. P.; Philip, R.; Joseph, V.; Singh, I. S. B. Anti-Lipopolysaccharide Factor and Crustin-III, the Anti-White Spot Virus Peptides in Penaeus Monodon: Control of Viral Infection by up-Regulation. Aquaculture 2011, 319 (1–2), 11–17. https://doi.org/10.1016/j.aquaculture.2011.06.022.
258. Wang, H.; Zhang, J. X.; Wang, Y.; Fang, W. H.; Wang, Y.; Zhou, J. F.; Zhao, S.; Li, X. C. Newly Identified Type II Crustin (SpCrus2) in Scylla Paramamosain Contains a Distinct Cysteine Distribution Pattern Exhibiting Broad Antimicrobial Activity. Dev. Comp. Immunol. 2018, 84, 1–13. https://doi.org/10.1016/j.dci.2018.01.021.
259. Wang, Y.; Zhang, X. W.; Wang, H.; Fang, W. H.; Ma, H.; Zhang, F.; Wang, Y.; Li, X. C. SpCrus3 and SpCrus4 Share High Similarity in Mud Crab (Scylla Paramamosain) Exhibiting Different Antibacterial Activities. Dev. Comp. Immunol. 2018, 82, 139–151. https://doi.org/10.1016/j.dci.2018.01.006.
260. Wang, Y.; Zhang, C.; Wang, H.; Ma, H.; Huang, Y.-Q.; Lu, J.-X.; Li, X.-C.; Zhang, X.-W. Involvement of a Newly Identified Atypical Type II Crustin (SpCrus5) in the Antibacterial Immunity of Mud Crab Scylla Paramamosain. Fish Shellfish Immunol. 2018, 75, 346–356. https://doi.org/10.1016/j.fsi.2018.02.026.
261. Du, Z.; Wang, Y.; Ma, H.; Shen, X.; Wang, K.; Du, J.; Yu, X.; Fang, W.; Li, X. A New Crustin Homologue (SpCrus6) Involved in the Antimicrobial and Antiviral Innate Immunity in Mud Crab, Scylla Paramamosain. Fish Shellfish Immunol. 2019, 84 (July 2018), 733–743. https://doi.org/10.1016/j.fsi.2018.10.072.
262. Amparyup, P.; Donpudsa, S.; Tassanakajon, A. Shrimp Single WAP Domain (SWD)-Containing Protein Exhibits Proteinase Inhibitory and Antimicrobial Activities. Dev. Comp. Immunol. 2008, 32 (12), 1497–1509. https://doi.org/10.1016/j.dci.2008.06.005.
263. Xiao, B.; Wang, Y.; Xian, D.; Fan, T.; He, J.; Li, C. Identification of a Double-β-Defensin with Multiple Antimicrobial Activities in a Marine Invertebrate. J. Immunol. 2023, 210 (9), 1324–1337. https://doi.org/10.4049/jimmunol.2200817.
264. Montero-Alejo, V.; Corzo, G.; Porro-Suardíaz, J.; Pardo-Ruiz, Z.; Perera, E.; Rodríguez-Viera, L.; Sánchez-Díaz, G.; Hernán-dez-Rodríguez, E. W.; Álvarez, C.; Peigneur, S.; Tytgat, J.; Perdomo-Morales, R. Panusin Represents a New Family of β-Defensin-like Peptides in Invertebrates. Dev. Comp. Immunol. 2017, 67, 310–321. https://doi.org/10.1016/j.dci.2016.09.002.
265. Xie, Y.; Wan, H.; Zeng, X.; Zhang, Z.; Wang, Y. Characterization and Antimicrobial Evaluation of a New Spgly-AMP, Gly-cine-Rich Antimicrobial Peptide from the Mud Crab Scylla Paramamosain. Fish Shellfish Immunol. 2020, 106 (July), 384–392. https://doi.org/10.1016/j.fsi.2020.08.009.
266. Sperstad, S. V.; Haug, T.; Vasskog, T.; Stensvåg, K. Hyastatin, a Glycine-Rich Multi-Domain Antimicrobial Peptide Isolated from the Spider Crab (Hyas Araneus) Hemocytes. Mol. Immunol. 2009, 46 (13), 2604–2612. https://doi.org/10.1016/j.molimm.2009.05.002.
267. Shan, Z.; Zhu, K.; Peng, H.; Chen, B.; Liu, J.; Chen, F.; Ma, X.; Wang, S.; Qiao, K.; Wang, K. The New Antimicrobial Peptide SpHyastatin from the Mud Crab Scylla Paramamosain with Multiple Antimicrobial Mechanisms and High Effect on Bacterial Infection. Front. Microbiol. 2016, 7 (JUL), 1–14. https://doi.org/10.3389/fmicb.2016.01140.
268. Moe, M. K.; Haug, T.; Sydnes, M. O.; Sperstad, S. V.; Li, C.; Vaagsfjord, L. C.; de la Vega, E.; Stensvåg, K. Paralithocins, An-timicrobial Peptides with Unusual Disulfide Connectivity from the Red King Crab, Paralithodes Camtschaticus. J. Nat. Prod. 2018, 81 (1), 140–150. https://doi.org/10.1021/acs.jnatprod.7b00780.
269. Kang, C.-J.; Xue, J.-F.; Liu, N.; Zhao, X.-F.; Wang, J.-X. Characterization and Expression of a New Subfamily Member of Pe-naeidin Antimicrobial Peptides (Penaeidin 5) from Fenneropenaeus Chinensis. Mol. Immunol. 2007, 44 (7), 1535–1543. https://doi.org/10.1016/j.molimm.2006.08.025.
270. Wu, B.; Zhang, C.; Qin, X.; Shi, L.; Zhao, M. Identification and Function of Penaeidin 3 and Penaeidin 5 in Fenneropenaeus Merguiensis. Fish Shellfish Immunol. 2019, 89 (April), 623–631. https://doi.org/10.1016/j.fsi.2019.04.032.
271. Cuthbertson, B. J.; Büllesbach, E. E.; Gross, P. S. Discovery of Synthetic Penaeidin Activity against Antibiotic‐resistant Fungi. Chem. Biol. Drug Des. 2006, 68 (2), 120‐127. https://doi.org/10.1111/j.1747-0285.2006.00417.x.
272. Cuthbertson, B. J.; Deterding, L. J.; Williams, J. G.; Tomer, K. B.; Etienne, K.; Blackshear, P. J.; Büllesbach, E. E.; Gross, P. S. Diversity in Penaeidin Antimicrobial Peptide Form and Function. Dev. Comp. Immunol. 2008, 32 (3), 167–181. https://doi.org/10.1016/j.dci.2007.06.009.
273. Cuthbertson, B. J.; Büllesbach, E. E.; Fievet, J.; Bachère, E.; Gross, P. S. A New Class (Penaeidin Class 4) of Antimicrobial Peptides from the Atlantic White Shrimp (Litopenaeus Setiferus) Exhibits Target Specificity and an Independent Proline-Rich-Domain Function. Biochem. J. 2004, 381 (1), 79–86. https://doi.org/10.1042/BJ20040330.

274. Xiao, B.; Fu, Q.; Niu, S.; Zhu, P.; He, J.; Li, C. Penaeidins Restrict White Spot Syndrome Virus Infection by Antagonizing the Envelope Proteins to Block Viral Entry. Emerg. Microbes Infect. 2020, 9 (1), 390–412. https://doi.org/10.1080/22221751.2020.1729068.
275. Xiao, B.; Liao, X.; Wang, H.; He, J.; Li, C. BigPEN, an Antimicrobial Peptide of Penaeidin Family from Shrimp Litopenaeus Vannamei with Membrane Permeable and DNA Binding Activity. Fish Shellfish Immunol. Reports 2021, 2, 100034. https://doi.org/10.1016/j.fsirep.2021.100034.
276. An, M.-Y.; Gao, J.; Zhao, X.-F.; Wang, J.-X. A New Subfamily of Penaeidin with an Additional Serine-Rich Region from Kuruma Shrimp (Marsupenaeus Japonicus) Contributes to Antimicrobial and Phagocytic Activities. Dev. Comp. Immunol. 2016, 59, 186–198. https://doi.org/10.1016/j.dci.2016.02.001.
277. Destoumieux, D.; Bulet, P.; Strub, J.-M.; van Dorsselaer, A.; Bachère, E. Recombinant Expression and Range of Activity of Penaeidins, Antimicrobial Peptides from Penaeid Shrimp. Eur. J. Biochem. 1999, 266 (2), 335–346. https://doi.org/10.1046/j.1432-1327.1999.00855.x.
278. Destoumieux, D.; Bulet, P.; Loew, D.; Van Dorsselaer, A.; Rodriguez, J.; Bachère, E. Penaeidins, a New Family of Antimicrobial Peptides Isolated from the Shrimp Penaeus Vannamei (Decapoda). J. Biol. Chem. 1997, 272 (45), 28398–28406. https://doi.org/10.1074/jbc.272.45.28398.
279. Imjongjirak, C.; Amphaiphan, P.; Charoensapsri, W.; Amparyup, P. Characterization and Antimicrobial Evaluation of Sp PR-AMP1, a Proline-Rich Antimicrobial Peptide from the Mud Crab Scylla Paramamosain. Dev. Comp. Immunol. 2017, 74, 209–216. https://doi.org/10.1016/j.dci.2017.05.003.
280. Huang, W. S.; Wang, K. J.; Yang, M.; Cai, J. J.; Li, S. J.; Wang, G. Z. Purification and Part Characterization of a Novel Anti-bacterial Protein Scygonadin, Isolated from the Seminal Plasma of Mud Crab, Scylla Serrata (Forskål, 1775). J. Exp. Mar. Bio. Ecol. 2006, 339 (1), 37–42. https://doi.org/10.1016/j.jembe.2006.06.029.
281. Peng, H.; Yang, M.; Huang, W.-S.; Ding, J.; Qu, H.-D.; Cai, J.-J.; Zhang, N.; Wang, K.-J. Soluble Expression and Purification of a Crab Antimicrobial Peptide Scygonadin in Different Expression Plasmids and Analysis of Its Antimicrobial Activity. Protein Expr. Purif. 2010, 70 (1), 109–115. https://doi.org/10.1016/j.pep.2009.09.008.
282. Peng, H.; Liu, H. P.; Chen, B.; Hao, H.; Wang, K. J. Optimized Production of Scygonadin in Pichia Pastoris and Analysis of Its Antimicrobial and Antiviral Activities. Protein Expr. Purif. 2012, 82 (1), 37–44. https://doi.org/10.1016/j.pep.2011.11.008.
283. Yedery, R. D.; Reddy, K. V. R. Purification and Characterization of Antibacterial Proteins from Granular Hemocytes of Indian Mud Crab , Scylla Serrata. Acta Biochim. Pol. 2009, 56 (1), 71–82.
284. Yang, Y.; Chen, F.; Chen, H.-Y.; Peng, H.; Hao, H.; Wang, K.-J. A Novel Antimicrobial Peptide Scyreprocin From Mud Crab Scylla Paramamosain Showing Potent Antifungal and Anti-Biofilm Activity. Front. Microbiol. 2020, 11 (July). https://doi.org/10.3389/fmicb.2020.01589.
285. Zhu, X.; Chen, F.; Li, S.; Peng, H.; Wang, K.-J. A Novel Antimicrobial Peptide Sparanegtin Identified in Scylla Paramamosain Showing Antimicrobial Activity and Immunoprotective Role In Vitro and Vivo. Int. J. Mol. Sci. 2021, 23 (1), 15. https://doi.org/10.3390/ijms23010015.
286. Rolland, J. L.; Abdelouahab, M.; Dupont, J.; Lefevre, F.; Bachère, E.; Romestand, B. Stylicins, a New Family of Antimicrobial Peptides from the Pacific Blue Shrimp Litopenaeus Stylirostris. Mol. Immunol. 2010, 47 (6), 1269–1277. https://doi.org/10.1016/j.molimm.2009.12.007.
287. Liu, H.; Wang, J.; Mao, Y.; Liu, M.; Niu, S.; Qiao, Y.; Su, Y.; Wang, C.; Zheng, Z. Identification and Expression Analysis of a Novel Stylicin Antimicrobial Peptide from Kuruma Shrimp (Marsupenaeus Japonicus). Fish Shellfish Immunol. 2015, 47 (2), 817–823. https://doi.org/10.1016/j.fsi.2015.09.044.
288. Park, J.; Kang, H. K.; Choi, M.-C.; Chae, J. D.; Son, B. K.; Chong, Y. P.; Seo, C. H.; Park, Y. Antibacterial Activity and Mechanism of Action of Analogues Derived from the Antimicrobial Peptide MBjAMP1 Isolated from Branchiostoma Japonicum. J. Anti-microb. Chemother. 2018, 73 (8), 2054–2063. https://doi.org/10.1093/jac/dky144.
289. Nam, J.; Yun, H.; Rajasekaran, G.; Kumar, S. D.; Kim, J. Il; Min, H. J.; Shin, S. Y.; Lee, C. W. Structural and Functional As-sessment of MBjAMP1, an Antimicrobial Peptide from Branchiostoma Japonicum , Revealed a Novel α-Hairpinin-like Scaffold with Membrane Permeable and DNA Binding Activity. J. Med. Chem. 2018, 61 (24), 11101–11113. https://doi.org/10.1021/acs.jmedchem.8b01135.
290. Teng, L.; Gao, B.; Zhang, S. The First Chordate Big Defensin: Identification, Expression and Bioactivity. Fish Shellfish Immunol. 2012, 32 (4), 572–577. https://doi.org/10.1016/j.fsi.2012.01.007.
291. Lee, I. H.; Cho, Y.; Lehrer, R. I. Effects of PH and Salinity on the Antimicrobial Properties of Clavanins. Infect. Immun. 1997, 65 (7), 2898–2903. https://doi.org/10.1128/iai.65.7.2898-2903.1997.
292. Silva, O. N.; Fensterseifer, I. C. M.; Rodrigues, E. A.; Holanda, H. H. S.; Novaes, N. R. F.; Cunha, J. P. A.; Rezende, T. M. B.; Magalhães, K. G.; Moreno, S. E.; Jerônimo, M. S.; Bocca, A. L.; Franco, O. L. Clavanin a Improves Outcome of Complications from Different Bacterial Infections. Antimicrob. Agents Chemother. 2015, 59 (3), 1620–1626. https://doi.org/10.1128/AAC.03732-14.
293. Silva, O. N.; de la Fuente-Núñez, C.; Haney, E. F.; Fensterseifer, I. C. M.; Ribeiro, S. M.; Porto, W. F.; Brown, P.; Faria-Junior, C.; Rezende, T. M. B.; Moreno, S. E.; Lu, T. K.; Hancock, R. E. W.; Franco, O. L. An Anti-Infective Synthetic Peptide with Dual Antimicrobial and Immunomodulatory Activities. Sci. Rep. 2016, 6 (1), 35465. https://doi.org/10.1038/srep35465.
294. Miller, A.; Matera-Witkiewicz, A.; Mikołajczyk, A.; Wieczorek, R.; Rowińska-Żyrek, M. Chemical “Butterfly Effect” Explaining the Coordination Chemistry and Antimicrobial Properties of Clavanin Complexes. Inorg. Chem. 2021, 60 (17), 12730–12734. https://doi.org/10.1021/acs.inorgchem.1c02101.
295. Peng, Z.; Wei, C.; Chen, M.; Wang, H.; Zou, Z.; Li, B.; Chen, J. Clavf Derived from Clavanins as a Promising Candidate for Fighting Infections from Aeromonas Hydrophila. 2024. https://doi.org/10.2139/ssrn.4974131.
296. Lee, I. H.; Zhao, C.; Nguyen, T.; Menzel, L.; Waring, A. J.; Sherman, M. A.; Lehrer, R. I. Clavaspirin, an Antibacterial and Haemolytic Peptide from Styela Clava. J. Pept. Res. 2001, 58 (6), 445–456. https://doi.org/10.1034/j.1399-3011.2001.10975.x.
297. Lee, J.-K.; Luchian, T.; Park, Y. New Antimicrobial Peptide Kills Drug-Resistant Pathogens without Detectable Resistance. Oncotarget 2018, 9 (21), 15616–15634. https://doi.org/10.18632/oncotarget.24582.
298. Lee, I. H.; Lee, Y. S.; Kim, C. H.; Kim, C. R.; Hong, T.; Menzel, L.; Boo, L. M.; Pohl, J.; Sherman, M. A.; Waring, A.; Lehrer, R. I. Dicynthaurin: An Antimicrobial Peptide from Hemocytes of the Solitary Tunicate, Halocynthia Aurantium. Biochim. Biophys. Acta - Gen. Subj. 2001, 1527 (3), 141–148. https://doi.org/10.1016/S0304-4165(01)00156-8.
299. Jang, W. S.; Kim, C. H.; Kim, K. N.; Park, S. Y.; Lee, J. H.; Son, S. M.; Lee, I. H. Biological Activities of Synthetic Analogs of Halocidin, an Antimicrobial Peptide from the Tunicate Halocynthia Aurantium. Antimicrob. Agents Chemother. 2003, 47 (8), 2481–2486. https://doi.org/10.1128/AAC.47.8.2481-2486.2003.
300. Jang, W. S.; Kim, H. K.; Lee, K. Y.; Kim, S. A.; Han, Y. S.; Lee, I. H. Antifungal Activity of Synthetic Peptide Derived from Halocidin, Antimicrobial Peptide from the Tunicate, Halocynthia Aurantium. FEBS Lett. 2006, 580 (5), 1490–1496. https://doi.org/10.1016/j.febslet.2006.01.041.
301. Jang, W. S.; Lee, S.-C.; Lee, Y. S.; Shin, Y. P.; Shin, K. H.; Sung, B. H.; Kim, B. S.; Lee, S. H.; Lee, I. H. Antimicrobial Effect of Halocidin-Derived Peptide in a Mouse Model of Listeria Infection. Antimicrob. Agents Chemother. 2007, 51 (11), 4148–4156. https://doi.org/10.1128/AAC.00635-07.
302. Han, J.; Jyoti, M. A.; Song, H. Y.; Jang, W. S. Antifungal Activity and Action Mechanism of Histatin 5-Halocidin Hybrid Peptides against Candida Ssp. PLoS One 2016, 11 (2), 1–18. https://doi.org/10.1371/journal.pone.0150196.
303. Jang, W. S.; Kim, K. N.; Lee, Y. S.; Nam, M. H.; Lee, I. H. Halocidin: A New Antimicrobial Peptide from Hemocytes of the Solitary Tunicate, Halocynthia Aurantium. FEBS Lett. 2002, 521 (1–3), 81–86. https://doi.org/10.1016/S0014-5793(02)02827-2.
304. Galinier, R.; Roger, E.; Sautiere, P. E.; Aumelas, A.; Banaigs, B.; Mitta, G. Halocyntin and Papillosin, Two New Antimicrobial Peptides Isolated from Hemocytes of the Solitary Tunicate, Halocynthia Papillosa. J. Pept. Sci. 2009, 15 (1), 48–55. https://doi.org/10.1002/psc.1101.
305. Fedders, H.; Michalek, M.; Grötzinger, J.; Leippe, M. An Exceptional Salt-Tolerant Antimicrobial Peptide Derived from a Novel Gene Family of Haemocytes of the Marine Invertebrate Ciona Intestinalis. Biochem. J. 2008, 416 (1), 65–75. https://doi.org/10.1042/BJ20080398.

306. Fedders, H.; Leippe, M. A Reverse Search for Antimicrobial Peptides in Ciona Intestinalis: Identification of a Gene Family Expressed in Hemocytes and Evaluation of Activity. Dev. Comp. Immunol. 2008, 32 (3), 286–298. https://doi.org/10.1016/j.dci.2007.06.003.
307. Lu, Y.; Zhuang, Y.; Liu, J. Mining Antimicrobial Peptides from Small Open Reading Frames in Ciona Intestinalis. J. Pept. Sci. 2014, 20 (1), 25–29. https://doi.org/10.1002/psc.2584.
308. Lee, I. H.; Cho, Y.; Lehrer, R. I. Styelins, Broad-Spectrum Antimicrobial Peptides from the Solitary Tunicate, Styela Clava. Comp. Biochem. Physiol. Part B Biochem. Mol. Biol. 1997, 118 (3), 515–521. https://doi.org/10.1016/S0305-0491(97)00109-0.
309. Taylor, S. W.; Craig, A. G.; Fischer, W. H.; Park, M.; Lehrer, R. I. Styelin D, an Extensively Modified Antimicrobial Peptide from Ascidian Hemocytes. J. Biol. Chem. 2000, 275 (49), 38417–38426. https://doi.org/10.1074/jbc.M006762200.
310. Hansen, I. K. Ø.; Lövdahl, T.; Simonovic, D.; Hansen, K. Ø.; Andersen, A. J. C.; Devold, H.; Richard, C. S. M.; Andersen, J. H.; Strøm, M. B.; Haug, T. Antimicrobial Activity of Small Synthetic Peptides Based on the Marine Peptide Turgencin A: Prediction of Antimicrobial Peptide Sequences in a Natural Peptide and Strategy for Optimization of Potency. Int. J. Mol. Sci. 2020, 21 (15), 5460. https://doi.org/10.3390/ijms21155460.
311. Hansen, I. K. Ø.; Isaksson, J.; Poth, A. G.; Hansen, K. Ø.; Andersen, A. J. C. C.; Richard, C. S. M. M.; Blencke, H.-M.; Stensvåg, K.; Craik, D. J.; Haug, T. Isolation and Characterization of Antimicrobial Peptides with Unusual Disulfide Connectivity from the Colonial Ascidian Synoicum Turgens. Mar. Drugs 2020, 18 (1), 51. https://doi.org/10.3390/md18010051.
312. Mason, B.; Cooke, I.; Moya, A.; Augustin, R.; Lin, M.-F.; Satoh, N.; Bosch, T. C. G.; Bourne, D. G.; Hayward, D. C.; Andrade, N.; Forêt, S.; Ying, H.; Ball, E. E.; Miller, D. J. AmAMP1 from Acropora Millepora and Damicornin Define a Family of Coral-Specific Antimicrobial Peptides Related to the Shk Toxins of Sea Anemones. Dev. Comp. Immunol. 2021, 114 (September 2020), 103866. https://doi.org/10.1016/j.dci.2020.103866.
313. Kim, C.-H. H.; Lee, Y. J.; Go, H.-J. J.; Oh, H. Y.; Lee, T. K.; Park, J. B.; Park, N. G. Defensin-Neurotoxin Dyad in a Basally Branching Metazoan Sea Anemone. FEBS J. 2017, 284 (19), 3320–3338. https://doi.org/10.1111/febs.14194.
314. Vidal-Dupiol, J.; Ladrière, O.; Destoumieux-Garzón, D.; Sautière, P.-E.; Meistertzheim, A.-L.; Tambutté, E.; Tambutté, S.; Duval, D.; Fouré, L.; Adjeroud, M.; Mitta, G. Innate Immune Responses of a Scleractinian Coral to Vibriosis. J. Biol. Chem. 2011, 286 (25), 22688–22698. https://doi.org/10.1074/jbc.M110.216358.
315. Driscoll, P. C.; Gronenborn, A. M.; Beress, L.; Clore, G. M.; Clare, G. M. Determination of the Three-Dimensional Solution Structure of the Antihypertensive and Antiviral Protein BDS-I from the Sea Anemone Anemonia Sulcata: A Study Using Nu-clear Magnetic Resonance and Hybrid Distance Geometry–Dynamical Simulated Annealing. Biochemistry 1989, 28 (5), 2188–2198. https://doi.org/10.1021/bi00431a033.
316. La Corte, C.; Catania, V.; Dara, M.; Parrinello, D.; Staropoli, M.; Trapani, M. R.; Cammarata, M.; Parisi, M. G. Equinins as Novel Broad-Spectrum Antimicrobial Peptides Isolated from the Cnidarian Actinia Equina (Linnaeus, 1758). Mar. Drugs 2024, 22 (4), 172. https://doi.org/10.3390/md22040172.
317. Lima, L.; Migliolo, L.; Castro, C.; Pires, D.; Lopez-Abarrategui, C.; Goncalves, E.; Vasconcelos, I.; Oliveira, J.; Otero-Gonzalez, A.; Franco, O.; Dias, S. Identification of a Novel Antimicrobial Peptide from Brazilian Coast Coral Phyllogorgia Dilatata. Protein Pept. Lett. 2013, 20 (10), 1153–1158. https://doi.org/10.2174/0929866511320100010.
318. Augustin, R.; Anton-Erxleben, F.; Jungnickel, S.; Hemmrich, G.; Spudy, B.; Podschun, R.; Bosch, T. C. G. Activity of the Novel Peptide Arminin against Multiresistant Human Pathogens Shows the Considerable Potential of Phylogenetically Ancient Or-ganisms as Drug Sources. Antimicrob. Agents Chemother. 2009, 53 (12), 5245–5250. https://doi.org/10.1128/AAC.00826-09.
319. Ovchinnikova, T. V.; Balandin, S. V.; Aleshina, G. M.; Tagaev, A. A.; Leonova, Y. F.; Krasnodembsky, E. D.; Men'shenin, A. V.; Kokryakov, V. N. Aurelin, a Novel Antimicrobial Peptide from Jellyfish Aurelia Aurita with Structural Features of Defensins and Channel-Blocking Toxins. Biochem. Biophys. Res. Commun. 2006, 348 (2), 514–523. https://doi.org/10.1016/j.bbrc.2006.07.078.
320. Shenkarev, Z. O.; Panteleev, P. V.; Balandin, S. V.; Gizatullina, A. K.; Altukhov, D. A.; Finkina, E. I.; Kokryakov, V. N.; Arseniev, A. S.; Ovchinnikova, T. V. Recombinant Expression and Solution Structure of Antimicrobial Peptide Aurelin from Jellyfish Aurelia Aurita. Biochem. Biophys. Res. Commun. 2012, 429 (1–2), 63–69. https://doi.org/10.1016/j.bbrc.2012.10.092.
321. Jung, S.; Dingley, A. J.; Augustin, R.; Anton-Erxleben, F.; Stanisak, M.; Gelhaus, C.; Gutsmann, T.; Hammer, M. U.; Podschun, R.; Bonvin, A. M. J. J.; Leippe, M.; Bosch, T. C. G.; Grötzinger, J. Hydramacin-1, Structure and Antibacterial Activity of a Protein from the Basal Metazoan Hydra. J. Biol. Chem. 2009, 284 (3), 1896–1905. https://doi.org/10.1074/jbc.M804713200.
322. Bosch, T. C. G.; Augustin, R.; Anton-Erxleben, F.; Fraune, S.; Hemmrich, G.; Zill, H.; Rosenstiel, P.; Jacobs, G.; Schreiber, S.; Leippe, M.; Stanisak, M.; Grötzinger, J.; Jung, S.; Podschun, R.; Bartels, J.; Harder, J.; Schröder, J.-M. M. Uncovering the Evo-lutionary History of Innate Immunity: The Simple Metazoan Hydra Uses Epithelial Cells for Host Defence. Dev. Comp. Im-munol. 2009, 33 (4), 559–569. https://doi.org/10.1016/j.dci.2008.10.004.
323. Kim, C.-H.; Go, H.-J.; Oh, H. Y.; Park, J. B.; Lee, T. K.; Seo, J.-K.; Elphick, M. R.; Park, N. G. Identification of a Novel Antimi-crobial Peptide from the Sea Star Patiria Pectinifera. Dev. Comp. Immunol. 2018, 86, 203–213. https://doi.org/10.1016/j.dci.2018.05.002.
324. Li, C.; Haug, T.; Moe, M. K.; Styrvold, O. B.; Stensvåg, K. Centrocins: Isolation and Characterization of Novel Dimeric Anti-microbial Peptides from the Green Sea Urchin, Strongylocentrotus Droebachiensis. Dev. Comp. Immunol. 2010, 34 (9), 959–968. https://doi.org/10.1016/j.dci.2010.04.004.
325. Solstad, R. G.; Li, C.; Isaksson, J.; Johansen, J.; Svenson, J.; Stensvåg, K.; Haug, T. Novel Antimicrobial Peptides EeCentrocins 1, 2 and EeStrongylocin 2 from the Edible Sea Urchin Echinus Esculentus Have 6-Br-Trp Post-Translational Modifications. PLoS One 2016, 11 (3), e0151820. https://doi.org/10.1371/journal.pone.0151820.
326. Solstad, R. G.; Johansen, C.; Stensvåg, K.; Strøm, M. B.; Haug, T. Structure-Activity Relationship Studies of Shortened Ana-logues of the Antimicrobial Peptide EeCentrocin 1 from the Sea Urchin Echinus Esculentus. J. Pept. Sci. 2020, 26 (2), 1–10. https://doi.org/10.1002/psc.3233.
327. Schillaci, D.; Cusimano, M. G.; Spinello, A.; Barone, G.; Russo, D.; Vitale, M.; Parrinello, D.; Arizza, V. Paracentrin 1, a Synthetic Antimicrobial Peptide from the Sea-Urchin Paracentrotus Lividus, Interferes with Staphylococcal and Pseudomonas Aeru-ginosa Biofilm Formation. AMB Express 2014, 4 (1), 78. https://doi.org/10.1186/s13568-014-0078-z.
328. Li, C.; Blencke, H. M.; Smith, L. C.; Karp, M. T.; Stensvåg, K. Two Recombinant Peptides, SpStrongylocins 1 and 2, from Strongylocentrotus Purpuratus, Show Antimicrobial Activity against Gram-Positive and Gram-Negative Bacteria. Dev. Comp. Immunol. 2010, 34 (3), 286–292. https://doi.org/10.1016/j.dci.2009.10.006.
329. Li, C.; Haug, T.; Styrvold, O. B.; Jørgensen, T. O.; Stensvåg, K. Strongylocins, Novel Antimicrobial Peptides from the Green Sea Urchin, Strongylocentrotus Droebachiensis. Dev. Comp. Immunol. 2008, 32 (12), 1430–1440. https://doi.org/10.1016/j.dci.2008.06.013.
330. Cusimano, M. G.; Spinello, A.; Barone, G.; Schillaci, D.; Cascioferro, S.; Magistrato, A.; Parrino, B.; Arizza, V.; Vitale, M. A Synthetic Derivative of Antimicrobial Peptide Holothuroidin 2 from Mediterranean Sea Cucumber (Holothuria Tubulosa) in the Control of Listeria Monocytogenes. Mar. Drugs 2019, 17 (3), 1–11. https://doi.org/10.3390/md17030159.
331. Schillaci, D.; Cusimano, M.; Cunsolo, V.; Saletti, R.; Russo, D.; Vazzana, M.; Vitale, M.; Arizza, V. Immune Mediators of Sea-Cucumber Holothuria Tubulosa (Echinodermata) as Source of Novel Antimicrobial and Anti-Staphylococcal Biofilm Agents. AMB Express 2013, 3 (1), 35. https://doi.org/10.1186/2191-0855-3-35.
332. Seo, J.-K.; Kim, D.-G.; Lee, J.-E.; Park, K.-S.; Lee, I.-A.; Lee, K.-Y.; Kim, Y.-O.; Nam, B.-H. Antimicrobial Activity and Action Mechanisms of Arg-Rich Short Analog Peptides Designed from the C-Terminal Loop Region of American Oyster Defensin (AOD). Mar. Drugs 2021, 19 (8), 451. https://doi.org/10.3390/md19080451.
333. Zhao, J.; Song, L.; Li, C.; Ni, D.; Wu, L.; Zhu, L.; Wang, H.; Xu, W. Molecular Cloning, Expression of a Big Defensin Gene from Bay Scallop Argopecten Irradians and the Antimicrobial Activity of Its Recombinant Protein. Mol. Immunol. 2007, 44 (4), 360–368. https://doi.org/10.1016/j.molimm.2006.02.025.

334. Seo, J.-K.; Crawford, J. M.; Stone, K. L.; Noga, E. J. Purification of a Novel Arthropod Defensin from the American Oyster, Crassostrea Virginica. Biochem. Biophys. Res. Commun. 2005, 338 (4), 1998–2004. https://doi.org/10.1016/j.bbrc.2005.11.013.
335. Gueguen, Y.; Herpin, A.; Aumelas, A.; Garnier, J.; Fievet, J.; Escoubas, J.-M.; Bulet, P.; Gonzalez, M.; Lelong, C.; Favrel, P.; Bachère, E. Characterization of a Defensin from the Oyster Crassostrea Gigas. J. Biol. Chem. 2006, 281 (1), 313–323. https://doi.org/10.1074/jbc.M510850200.
336. Schmitt, P.; Lorgeril, J. de; Gueguen, Y.; Destoumieux-Garzón, D.; Bachère, E. Expression, Tissue Localization and Synergy of Antimicrobial Peptides and Proteins in the Immune Response of the Oyster Crassostrea Gigas. Dev. Comp. Immunol. 2012, 37 (3–4), 363–370. https://doi.org/10.1016/j.dci.2012.01.004.
337. Adhya, M.; Jeung, H.-D.; Kang, H.-S.; Choi, K.-S.; Lee, D. S.; Cho, M. Cloning and Localization of MCdef, a Defensin from Manila Clams (Ruditapes Philippinarum). Comp. Biochem. Physiol. Part B Biochem. Mol. Biol. 2012, 161 (1), 25–31. https://doi.org/10.1016/j.cbpb.2011.09.003.
338. Hubert, F.; Noël, T.; Roch, P. A Member of the Arthropod Defensin Family from Edible Mediterranean Mussels (Mytilus Gal-loprovincialis). Eur. J. Biochem. 1996, 240 (1), 302–306. https://doi.org/10.1111/j.1432-1033.1996.0302h.x.
339. Zhang, Y.; Cui, P.; Wang, Y.; Zhang, S. Identification and Bioactivity Analysis of a Newly Identified Defensin from the Oyster Magallana Gigas. Dev. Comp. Immunol. 2018, 85, 177–187. https://doi.org/10.1016/j.dci.2018.04.014.
340. Revilla, J.; Stambuk, F.; Hurtado, L.; Rojas, R.; Aróstica, M.; Guzmán, F.; Cárdenas, C.; Álvarez, C. A.; Brokordt, K.; Schmitt, P. Unveiling Novel Scallop-Derived Antimicrobial Peptides Targeting Host-Associated Vibrios for Sustainable Pathogen Control in Aquaculture. Aquaculture 2024, 592 (June), 741238. https://doi.org/10.1016/j.aquaculture.2024.741238.
341. Wang, Y.; Zeng, Z.; Zhang, X.; Shi, Q.; Wang, C.; Hu, Z.; Li, H. Identification and Characterization of a Novel Defensin from Asian Green Mussel Perna Viridis. Fish Shellfish Immunol. 2018, 74 (August 2017), 242–249. https://doi.org/10.1016/j.fsi.2017.12.029.
342. Wei, Y.-X.; Guo, D.-S.; Li, R.-G.; Chen, H.-W.; Chen, P.-X. [Purification of a Big Defensin from Ruditapes Philippinesis and Its Antibacterial Activity]. Sheng Wu Hua Xue Yu Sheng Wu Wu Li Xue Bao (Shanghai). 2003, 35 (12), 1145–1148.
343. Yang, D.; Zhang, Q.; Wang, Q.; Chen, L.; Liu, Y.; Cong, M.; Wu, H.; Li, F.; Ji, C.; Zhao, J. A Defensin-like Antimicrobial Peptide from the Manila Clam Ruditapes Philippinarum : Investigation of the Antibacterial Activities and Mode of Action. Fish Shellfish Immunol. 2018, 80 (December 2017), 274–280. https://doi.org/10.1016/j.fsi.2018.06.019.
344. Zhao, J.; Li, C.; Chen, A.; Li, L.; Su, X.; Li, T. Molecular Characterization of a Novel Big Defensin from Clam Venerupis Phil-ippinarum. PLoS One 2010, 5 (10), e13480. https://doi.org/10.1371/journal.pone.0013480.
345. Zhang, L.; Yang, D.; Wang, Q.; Yuan, Z.; Wu, H.; Pei, D.; Cong, M.; Li, F.; Ji, C.; Zhao, J. A Defensin from Clam Venerupis Philippinarum: Molecular Characterization, Localization, Antibacterial Activity, and Mechanism of Action. Dev. Comp. Immunol. 2015, 51 (1), 29–38. https://doi.org/10.1016/j.dci.2015.02.009.
346. Xu, Q.; Wang, G.; Yuan, H.; Chai, Y.; Xiao, Z. CDNA Sequence and Expression Analysis of an Antimicrobial Peptide, The-romacin, in the Triangle-Shell Pearl Mussel Hyriopsis Cumingii. Comp. Biochem. Physiol. Part B Biochem. Mol. Biol. 2010, 157 (1), 119 – 126. https://doi.org/10.1016/j.cbpb.2010.05.010.
347. Liu, L.; Yang, J. Y.; Yang, Z. X.; He, J. Y.; Zhang, X. L.; He, M. L.; Yan, X. J.; Liao, Z. Molecular Characterization of Two Novel Antimicrobial Peptides Myticalin and Mytimacin From Mytilus Coruscus. Acta Hydrobiol. Sin. 2022, 46 (12), 1888–1899. https://doi.org/https://doi.org/10.7541/2022.2021.0392.
348. Lv, J.; Zhao, J.; Yang, D.; Wu, H.; Cong, M. Tissue Distribution and Functional Characterization of Mytimacin-4 in Mytilus Galloprovincialis. J. Invertebr. Pathol. 2019, 166 (March), 107215. https://doi.org/10.1016/j.jip.2019.107215.
349. Dong, B.; Wang, Y.; Cui, G.; Wang, Y.; Lin, Y.; Su, Z.; Zhao, G. In Vitro Antimicrobial Activity of the Novel Antimicrobial Peptide Mytimacin-4 and Its Influence on the Microbial Community and Quality of Pork during Refrigerated Storage. Food Control 2024, 163 (December 2023), 110486. https://doi.org/10.1016/j.foodcont.2024.110486.
350. Lee, Y.; Lee, S.; Whang, I.; Oh, C. First Molluscan Antimicrobial Peptide Hydramacin in Manila Clam: Molecular Characteri-zation and Expression Analysis. J. Coast. Life Med. 2014, No. June. https://doi.org/10.12980/JCLM.2.201414J6.
351. Zhang, G.; Wang, G.; Guo, S.; Bai, Z.; Li, J. Full-Length CDNA Cloning and Expression Analysis of Theromacin Gene in An-odonta Woodiana. J. Fish. China 2014, 38 (5), 662–670.
352. Yang, D.; Han, Y.; Chen, L.; Cao, R.; Wang, Q.; Dong, Z.; Liu, H.; Zhang, X.; Zhang, Q.; Zhao, J. A Macin Identified from Venerupis Philippinarum: Investigation on Antibacterial Activities and Action Mode. Fish Shellfish Immunol. 2019, 92 (July), 897–904. https://doi.org/10.1016/j.fsi.2019.07.031.
353. Xue, R.; Han, Y.; Li, F.; Chen, L.; Yang, D.; Zhao, J. Identification, Antibacterial Activities and Action Mode of Two Macins from Manila Clam Venerupis Philippinarum. Fish Shellfish Immunol. 2021, 118 (July), 411–420. https://doi.org/10.1016/j.fsi.2021.09.031.
354. Hong, S.-Y.; Kim, D.-G.; Kim, Y.-O.; Park, J. Y.; Seo, J.-K.; Nam, B.-H.; Hong, Y.-K. Purification and CDNA Cloning of the Antimicrobial Peptide ApMolluscidin from the Pen Shell, Atrina Pectinata. Fish Shellfish Immunol. 2018, 81 (May), 408–415. https://doi.org/10.1016/j.fsi.2018.07.044.
355. Seo, J.-K.; Lee, M. J.; Nam, B.-H.; Park, N. G. CgMolluscidin, a Novel Dibasic Residue Repeat Rich Antimicrobial Peptide, Purified from the Gill of the Pacific Oyster, Crassostrea Gigas. Fish Shellfish Immunol. 2013, 35 (2), 480–488. https://doi.org/10.1016/j.fsi.2013.05.010.
356. Leoni, G.; De Poli, A.; Mardirossian, M.; Gambato, S.; Florian, F.; Venier, P.; Wilson, D.; Tossi, A.; Pallavicini, A.; Gerdol, M. Myticalins: A Novel Multigenic Family of Linear, Cationic Antimicrobial Peptides from Marine Mussels (Mytilus Spp.). Mar. Drugs 2017, 15 (8), 261. https://doi.org/10.3390/md15080261.
357. Pacor, S.; Benincasa, M.; Musso, M. V.; Krce, L.; Aviani, I.; Pallavicini, A.; Scocchi, M.; Gerdol, M.; Mardirossian, M. The Proline-Rich Myticalins from Mytilus Galloprovincialis Display a Membrane-Permeabilizing Antimicrobial Mode of Action. Peptides 2021, 143 (May), 170594. https://doi.org/10.1016/j.peptides.2021.170594.
358. Qin, C.; Huang, W.; Zhou, S.; Wang, X.; Liu, H.; Fan, M.; Wang, R.; Gao, P.; Liao, Z. Characterization of a Novel Antimicrobial Peptide with Chiting-Biding Domain from Mytilus Coruscus. Fish Shellfish Immunol. 2014, 41 (2), 362–370. https://doi.org/10.1016/j.fsi.2014.09.019.
359. Oh, R.; Lee, M. J.; Kim, Y.-O.; Nam, B.-H.; Kong, H. J.; Kim, J.-W.; Park, J. Y.; Seo, J.-K.; Kim, D.-G. Purification and Charac-terization of an Antimicrobial Peptide Mytichitin-Chitin Binding Domain from the Hard-Shelled Mussel, Mytilus Coruscus. Fish Shellfish Immunol. 2018, 83, 425–435. https://doi.org/10.1016/j.fsi.2018.09.009.
360. Domeneghetti, S.; Franzoi, M.; Damiano, N.; Norante, R.; M. El Halfawy, N.; Mammi, S.; Marin, O.; Bellanda, M.; Venier, P. Structural and Antimicrobial Features of Peptides Related to Myticin C, a Special Defense Molecule from the Mediterranean Mussel Mytilus Galloprovincialis. J. Agric. Food Chem. 2015, 63 (42), 9251–9259. https://doi.org/10.1021/acs.jafc.5b03491.
361. Mitta, G.; Hubert, F.; Noël, T.; Roch, P. Myticin, a Novel Cysteine - rich Antimicrobial Peptide Isolated from Haemocytes and Plasma of the Mussel Mytilus Galloprovincialis. Eur. J. Biochem. 1999, 265 (1), 71 – 78. https://doi.org/10.1046/j.1432-1327.1999.00654.x.
362. Novoa, B.; Romero, A.; Álvarez, Á. L.; Moreira, R.; Pereiro, P.; Costa, M. M.; Dios, S.; Estepa, A.; Parra, F.; Figueras, A. Antiviral Activity of Myticin C Peptide from Mussel: An Ancient Defense against Herpesviruses. J. Virol. 2016, 90 (17), 7692–7702. https://doi.org/10.1128/JVI.00591-16.
363. Liao, Z.; Wang, X.; Liu, H.; Fan, M.; Sun, J.; Shen, W. Molecular Characterization of a Novel Antimicrobial Peptide from Mytilus Coruscus. Fish Shellfish Immunol. 2013, 34 (2), 610–616. https://doi.org/10.1016/j.fsi.2012.11.030.
364. Oh, R.; Lee, M. J.; Kim, Y.-O.; Nam, B.-H.; Kong, H. J.; Kim, J.-W.; Park, J.; Seo, J.-K.; Kim, D.-G. Myticusin-Beta, Antimicrobial Peptide from the Marine Bivalve, Mytilus Coruscus. Fish Shellfish Immunol. 2020, 99 (February), 342–352. https://doi.org/10.1016/j.fsi.2020.02.020.

365. Charlet, M.; Chernysh, S.; Philippe, H.; Hetru, C.; Hoffmann, J. A.; Bulet, P. Innate Immunity. Isolation of Several Cysteine-Rich Antimicrobial Peptides from the Blood of a Mollusc, Mytilus Edulis. J. Biol. Chem. 1996, 271 (36), 21808–21813. https://doi.org/10.1074/jbc.271.36.21808.
366. Mitta, G.; Vandenbulcke, F.; Hubert, F.; Salzet, M.; Roch, P. Involvement of Mytilins in Mussel Antimicrobial Defense. J. Biol. Chem. 2000, 275 (17), 12954–12962. https://doi.org/10.1074/jbc.275.17.12954.
367. Arenas, G.; Guzmán, F.; Cárdenas, C.; Mercado, L.; Marshall, S. H. A Novel Antifungal Peptide Designed from the Primary Structure of a Natural Antimicrobial Peptide Purified from Argopecten Purpuratus Hemocytes. Peptides 2009, 30 (8), 1405–1411. https://doi.org/10.1016/j.peptides.2009.05.019.
368. Gueguen, Y.; Bernard, R.; Julie, F.; Paulina, S.; Delphine, D.-G.; Franck, V.; Philippe, B.; Evelyne, B. Oyster Hemocytes Express a Proline-Rich Peptide Displaying Synergistic Antimicrobial Activity with a Defensin. Mol. Immunol. 2009, 46 (4), 516–522. https://doi.org/10.1016/j.molimm.2008.07.021.
369. Mao, F.; Bao, Y.; Wong, N.-K.; Huang, M.; Liu, K.; Zhang, X.; Yang, Z.; Yi, W.; Shu, X.; Xiang, Z.; Yu, Z.; Zhang, Y. Large-Scale Plasma Peptidomic Profiling Reveals a Novel, Nontoxic, Crassostrea Hongkongensis-Derived Antimicrobial Peptide against Foodborne Pathogens. Mar. Drugs 2021, 19 (8), 420. https://doi.org/10.3390/md19080420.
370. Maselli, V.; Galdiero, E.; Salzano, A. M.; Scaloni, A.; Maione, A.; Falanga, A.; Naviglio, D.; Guida, M.; Di Cosmo, A.; Galdiero, S. OctoPartenopin: Identification and Preliminary Characterization of a Novel Antimicrobial Peptide from the Suckers of Oc-topus Vulgaris. Mar. Drugs 2020, 18 (8), 380. https://doi.org/10.3390/md18080380.
371. Jayathilaka, E. H. T. T.; Rajapaksha, D. C.; Nikapitiya, C.; Lee, J.; De Zoysa, M.; Whang, I. Novel Antimicrobial Peptide "Oc-toprohibitin" against Multidrug Resistant Acinetobacter Baumannii. Pharmaceuticals 2022, 15 (8), 928. https://doi.org/10.3390/ph15080928.
372. Nikapitiya, C.; Dananjaya, S. H. S.; Chandrarathna, H. P. S. U.; De Zoysa, M.; Whang, I. Octominin: A Novel Synthetic An-ticandidal Peptide Derived from Defense Protein of Octopus Minor. Mar. Drugs 2020, 18 (1), 56. https://doi.org/10.3390/md18010056.
373. Jayasinghe, J. N. C.; Whang, I.; De Zoysa, M. Antifungal Efficacy of Antimicrobial Peptide Octominin II against Candida Albicans. Int. J. Mol. Sci. 2023, 24 (18), 14053. https://doi.org/10.3390/ijms241814053.
374. Rajapaksha, D. C.; Jayathilaka, E. H. T. T.; Edirisinghe, S. L.; Nikapitiya, C.; Lee, J.; Whang, I.; De Zoysa, M. Octopromycin: Antibacterial and Antibiofilm Functions of a Novel Peptide Derived from Octopus Minor against Multidrug-Resistant Aci-netobacter Baumannii. Fish Shellfish Immunol. 2021, 117 (April), 82–94. https://doi.org/10.1016/j.fsi.2021.07.019.
375. Benoist, L.; Houyvet, B.; Henry, J.; Corre, E.; Zanuttini, B.; Zatylny-Gaudin, C. In-Depth In Silico Search for Cuttlefish (Sepia Officinalis) Antimicrobial Peptides Following Bacterial Challenge of Haemocytes. Mar. Drugs 2020, 18 (9), 439. https://doi.org/10.3390/md18090439.
376. Houyvet, B.; Zanuttini, B.; Corre, E.; Le Corguillé, G.; Henry, J.; Zatylny-Gaudin, C. Design of Antimicrobial Peptides from a Cuttlefish Database. Amino Acids 2018, 50 (11), 1573–1582. https://doi.org/10.1007/s00726-018-2633-4.
377. González García, M.; Rodríguez, A.; Alba, A.; Vázquez, A. A.; Morales Vicente, F. E.; Pérez-Erviti, J.; Spellerberg, B.; Stenger, S.; Grieshober, M.; Conzelmann, C.; Münch, J.; Raber, H.; Kubiczek, D.; Rosenau, F.; Wiese, S.; Ständker, L.; Otero-González, A. New Antibacterial Peptides from the Freshwater Mollusk Pomacea Poeyana (Pilsbry, 1927). Biomolecules 2020, 10 (11), 1473. https://doi.org/10.3390/biom10111473.
378. Iijima, R.; Kisugi, J.; Yamazaki, M. A Novel Antimicrobial Peptide from the Sea Hare Dolabella Auricularia. Dev. Comp. Im-munol. 2003, 27 (4), 305–311. https://doi.org/10.1016/S0145-305X(02)00105-2.
379. Bitaab, M. A.; Siadat, S. O.; Pazooki, J.; Sefidbakht, Y. Antibacterial and Molecular Dynamics Study of the Dolabellanin B2 Isolated from Sea Slug, Peronia Peronii. Biosci. Biotechnol. Res. Asia 2015, 12 (3), 2023–2035. https://doi.org/10.13005/bbra/1870.
380. Jiao, C.; Ruan, J.; Sun, W.; Zhang, X.; Liu, X.; Sun, G.; Liu, C.; Sun, C.; Tian, X.; Yang, D.; Chen, L.; Wang, Z. Molecular Characterization, Expression and Antibacterial Function of a Macin, HdMac, from Haliotis Discus Hannai. J. Invertebr. Pathol. 2024, 204 (November 2023), 108113. https://doi.org/10.1016/j.jip.2024.108113.
381. Xie, Z.; Yao, T.; Ye, L.; Wang, J. Molecular Characterization and Expression Analysis of an Antimicrobial Peptide, Mytimacin-6, in the Small Abalone, Haliotis Diversicolor. Isr. J. Aquac. - Bamidgeh 2021, 73, 1–11. https://doi.org/10.46989/001c.25815.
382. Seo, J.-K.; Go, H.-J.; Kim, C.-H.; Nam, B.-H.; Park, N. G. Antimicrobial Peptide, HdMolluscidin, Purified from the Gill of the Abalone, Haliotis Discus. Fish Shellfish Immunol. 2016, 52, 289–297. https://doi.org/10.1016/j.fsi.2016.03.150.
383. Dolashka, P.; Moshtanska, V.; Borisova, V.; Dolashki, A.; Stevanovic, S.; Dimanov, T.; Voelter, W. Antimicrobial Proline-Rich Peptides from the Hemolymph of Marine Snail Rapana Venosa. Peptides 2011, 32 (7), 1477–1483. https://doi.org/10.1016/j.peptides.2011.05.001.
384. Gauri, S. S.; Mandal, S. M.; Pati, B. R.; Dey, S. Purification and Structural Characterization of a Novel Antibacterial Peptide from Bellamya Bengalensis: Activity against Ampicillin and Chloramphenicol Resistant Staphylococcus Epidermidis. Peptides 2011, 32 (4), 691–696. https://doi.org/10.1016/j.peptides.2011.01.014.
385. López-Abarrategui, C.; Alba, A.; Silva, O. N.; Reyes-Acosta, O.; Vasconcelos, I. M.; Oliveira, J. T. A.; Migliolo, L.; Costa, M. P.; Costa, C. R.; Silva, M. R. R.; Garay, H. E.; Dias, S. C.; Franco, O. L.; Otero-González, A. J. Functional Characterization of a Synthetic Hydrophilic Antifungal Peptide Derived from the Marine Snail Cenchritis Muricatus. Biochimie 2012, 94 (4), 968–974. https://doi.org/10.1016/j.biochi.2011.12.016.
386. López-Abarrategui, C.; McBeth, C.; Mandal, S. M.; Sun, Z. J.; Heffron, G.; Alba-Menéndez, A.; Migliolo, L.; Reyes-Acosta, O.; García-Villarino, M.; Nolasco, D. O.; Falcão, R.; Cherobim, M. D.; Dias, S. C.; Brandt, W.; Wessjohann, L.; Starnbach, M.; Franco, O. L.; Otero-González, A. J. Cm-P5: An Antifungal Hydrophilic Peptide Derived from the Coastal Mollusk Cenchritis Muricatus (Gastropoda: Littorinidae). FASEB J. 2015, 29 (8), 3315–3325. https://doi.org/10.1096/fj.14-269860.
387. Rončević, T.; Gerdol, M.; Mardirossian, M.; Maleš, M.; Cvjetan, S.; Benincasa, M.; Maravić, A.; Gajski, G.; Krce, L.; Aviani, I.; Hrabar, J.; Trumbić, Ž.; Derks, M.; Pallavicini, A.; Weingarth, M.; Zoranić, L.; Tossi, A.; Mladineo, I. Anisaxins, Helical Anti-microbial Peptides from Marine Parasites, Kill Resistant Bacteria by Lipid Extraction and Membrane Disruption. Acta Biomater. 2022, 146, 131–144. https://doi.org/10.1016/j.actbio.2022.04.025.
388. Simunić, J.; Petrov, D.; Bouceba, T.; Kamech, N.; Benincasa, M.; Juretić, D. Trichoplaxin — A New Membrane-Active Anti-microbial Peptide from Placozoan CDNA. Biochim. Biophys. Acta - Biomembr. 2014, 1838 (5), 1430–1438. https://doi.org/10.1016/j.bbamem.2014.02.003.
389. Juretić, D.; Golemac, A.; Strand, D. E.; Chung, K.; Ilić, N.; Goić-Barišić, I.; Pellay, F.-X. The Spectrum of Design Solutions for Improving the Activity-Selectivity Product of Peptide Antibiotics against Multidrug-Resistant Bacteria and Prostate Cancer PC-3 Cells. Molecules 2020, 25 (15), 3526. https://doi.org/10.3390/molecules25153526.
390. Quinn, G. A. P.; Heymans, R.; Rondaj, F.; Shaw, C.; de Jong-Brink, M. Schistosoma Mansoni Dermaseptin-like Peptide: Structural and Functional Characterization. J. Parasitol. 2005, 91 (6), 1340–1351. https://doi.org/10.1645/GE-540R.1.
391. Wiens, M.; Schröder, H. C.; Korzhev, M.; Wang, X.-H.; Batel, R.; Müller, W. E. G. Inducible ASABF-Type Antimicrobial Peptide from the Sponge Suberites Domuncula: Microbicidal and Hemolytic Activity in Vitro and Toxic Effect on Molluscs in Vivo. Mar. Drugs 2011, 9 (10), 1969–1994. https://doi.org/10.3390/md9101969.
392. Seo, J.-K.; Nam, B.-H.; Go, H.-J.; Jeong, M.; Lee, K.-Y.; Cho, S.-M.; Lee, I.-A.; Park, N. G. Hemerythrin-Related Antimicrobial Peptide, MsHemerycin, Purified from the Body of the Lugworm, Marphysa Sanguinea. Fish Shellfish Immunol. 2016, 57, 49–59. https://doi.org/10.1016/j.fsi.2016.08.018.
393. Nam, B.-H. H.; Seo, J.-K. K.; Lee, M. J.; Kim, Y.-O. O.; Kim, D.-G. G.; An, C. M.; Park, N. G. Functional Analysis of Pacific Oyster (Crassostrea Gigas) β-Thymosin: Focus on Antimicrobial Activity. Fish Shellfish Immunol. 2015, 45 (1), 167–174. https://doi.org/10.1016/j.fsi.2015.03.035.
394. Arockiaraj, J.; Gnanam, A. J.; Kumaresan, V.; Palanisamy, R.; Bhatt, P.; Thirumalai, M. K.; Roy, A.; Pasupuleti, M.; Kasi, M. An Unconventional Antimicrobial Protein Histone from Freshwater Prawn Macrobrachium Rosenbergii: Analysis of Immune Properties. Fish Shellfish Immunol. 2013, 35 (5), 1511–1522. https://doi.org/10.1016/j.fsi.2013.08.018.

395. Mai, W.; Hu, C. CDNA Cloning, Expression and Antibacterial Activity of Lysozyme C in the Blue Shrimp (Litopenaeus Styli-rostris). Prog. Nat. Sci. 2009, 19 (7), 837–844. https://doi.org/10.1016/j.pnsc.2008.10.008.

396. Supungul, P.; Rimphanitchayakit, V.; Aoki, T.; Hirono, I.; Tassanakajon, A. Molecular Characterization and Expression Analysis of a C-Type and Two Novel Muramidase-Deficient i-Type Lysozymes from Penaeus Monodon. Fish Shellfish Immunol. 2010, 28 (3), 490–498. https://doi.org/10.1016/j.fsi.2010.01.003.

397. Ma, Z.; Qu, B.; Yao, L.; Gao, Z.; Zhang, S. Identification and Functional Characterization of Ribosomal Protein S23 as a New Member of Antimicrobial Protein. Dev. Comp. Immunol. 2020, 110 (May), 103730. https://doi.org/10.1016/j.dci.2020.103730.

398. Tincu, J. A.; Menzel, L. P.; Azimov, R.; Sands, J.; Hong, T.; Waring, A. J.; Taylor, S. W.; Lehrer, R. I. Plicatamide, an Antimi-crobial Octapeptide from Styela Plicata Hemocytes. J. Biol. Chem. 2003, 278 (15), 13546–13553. https://doi.org/10.1074/jbc.M211332200.

399. Augustin, R.; Schröder, K.; Murillo Rincón, A. P.; Fraune, S.; Anton-Erxleben, F.; Herbst, E.-M. M.; Wittlieb, J.; Schwentner, M.; Grötzinger, J.; Wassenaar, T. M.; Bosch, T. C. G. G.; Rincón, A. P. M.; Fraune, S.; Anton-Erxleben, F.; Herbst, E.-M. M.; Wittlieb, J.; Schwentner, M.; Grötzinger, J.; Wassenaar, T. M.; Bosch, T. C. G. G. A Secreted Antibacterial Neuropeptide Shapes the Microbiome of Hydra. Nat. Commun. 2017, 8 (1), 698. https://doi.org/10.1038/s41467-017-00625-1.

400. Grafskaia, E. N.; Polina, N. F.; Babenko, V. V.; Kharlampieva, D. D.; Bobrovsky, P. A.; Manuvera, V. A.; Farafonova, T. E.; Anikanov, N. A.; Lazarev, V. N. Discovery of Novel Antimicrobial Peptides: A Transcriptomic Study of the Sea Anemone Cnidopus Japonicus. J. Bioinform. Comput. Biol. 2018, 16 (02), 1840006. https://doi.org/10.1142/S0219720018400061.

401. Logashina, Y. A.; Solstad, R. G.; Mineev, K. S.; Korolkova, Y. V.; Mosharova, I. V.; Dyachenko, I. A.; Palikov, V. A.; Palikova, Y. A.; Murashev, A. N.; Arseniev, A. S.; Kozlov, S. A.; Stensvåg, K.; Haug, T.; Andreev, Y. A. New Disulfide-Stabilized Fold Provides Sea Anemone Peptide to Exhibit Both Antimicrobial and TRPA1 Potentiating Properties. Toxins (Basel). 2017, 9 (5), 154. https://doi.org/10.3390/toxins9050154.

402. Takamatsu, N.; Shiba, T.; Muramoto, K.; Kamiya, H. Molecular Cloning of the Defense Factor in the Albumen Gland of the Sea Hare Aplysia Kurodai. FEBS Lett. 1995, 377 (3), 373–376. https://doi.org/10.1016/0014-5793(95)01375-X.

403. Yamazaki, M.; Ohye, H.; Kisugi, J.; Kamiya, H. Bacteriostatic and Cytolytic Activity of Purple Fluid from the Sea Hare. Dev. Comp. Immunol. 1990, 14 (4), 379–383. https://doi.org/10.1016/0145-305X(90)90030-I.

404. Kisugi, J.; Ohye, H.; Kamiya, H.; Yamazaki, M. Biopolymers from Marine Invertebrates. XIII. Characterization of an Antibac-terial Protein, Dolabellanin A, from the Albumen Gland of the Sea Hare, Dolabella Auricularia. Chem. Pharm. Bull. 1992, 40 (6), 1537–1539.

405. Yang, H.; Johnson, P. M.; Ko, K.-C.; Kamio, M.; Germann, M. W.; Derby, C. D.; Tai, P. C. Cloning, Characterization and Expression of Escapin, a Broadly Antimicrobial FAD-Containing <scp>l</Scp> -Amino Acid Oxidase from Ink of the Sea Hare Aplysia Californica. J. Exp. Biol. 2005, 208 (18), 3609–3622. https://doi.org/10.1242/jeb.01795.

406. Nam, B.-H.; Moon, J.; Park, E.; Kong, H.; Kim, Y.-O.; Kim, D.-G.; Kim, W.-J.; An, C.; Seo, J.-K. Antimicrobial and Antitumor Activities of Novel Peptides Derived from the Lipopolysaccharide- and β-1,3-Glucan Binding Protein of the Pacific Abalone Haliotis Discus Hannai. Mar. Drugs 2016, 14 (12), 227. https://doi.org/10.3390/md14120227.

407. Zhuang, J.; Coates, C. J.; Zhu, H.; Zhu, P.; Wu, Z.; Xie, L. Identification of Candidate Antimicrobial Peptides Derived from Abalone Hemocyanin. Dev. Comp. Immunol. 2015, 49 (1), 96–102. https://doi.org/10.1016/j.dci.2014.11.008.

408. Gonzalez, M.; Gueguen, Y.; Destoumieux-Garzón, D.; Romestand, B.; Fievet, J.; Pugnière, M.; Roquet, F.; Escoubas, J.-M.; Vandenbulcke, F.; Levy, O.; Sauné, L.; Bulet, P.; Bachère, E. Evidence of a Bactericidal Permeability Increasing Protein in an Invertebrate, the Crassostrea Gigas Cg -BPI. Proc. Natl. Acad. Sci. 2007, 104 (45), 17759–17764. https://doi.org/10.1073/pnas.0702281104.

409. He, C.; Yu, H.; Liu, W.; Su, H.; Shan, Z.; Bao, X.; Li, Y.; Fu, L.; Gao, X. A Goose-Type Lysozyme Gene in Japanese Scallop (Mizuhopecten Yessoensis): CDNA Cloning, MRNA Expression and Promoter Sequence Analysis. Comp. Biochem. Physiol. - B Biochem. Mol. Biol. 2012, 162 (1–3), 34–43. https://doi.org/10.1016/j.cbpb.2012.02.002.

410. Seo, J.-K.; Lee, M. J.; Go, H.-J.; Kim, G. Do; Jeong, H. Do; Nam, B.-H.; Park, N. G. Purification and Antimicrobial Function of Ubiquitin Isolated from the Gill of Pacific Oyster, Crassostrea Gigas. Mol. Immunol. 2013, 53 (1–2), 88–98. https://doi.org/10.1016/j.molimm.2012.07.003.

411. Santos, B. P. O.; Alves, E. S. F.; Ferreira, C. S.; Ferreira-Silva, A.; Góes-Neto, A.; Verly, R. M.; Lião, L. M.; Oliveira, S. C.; de Magalhães, M. T. Q. Schistocins: Novel Antimicrobial Peptides Encrypted in the Schistosoma Mansoni Kunitz Inhibitor SmKI-1. Biochim. Biophys. Acta - Gen. Subj. 2021, 1865 (11), 129989. https://doi.org/10.1016/j.bbagen.2021.129989.
